# Heterotrimetallic Carbon Dioxide Copolymerization and Switchable Catalysts: Sodium is the Key to High Activity and Unusual Selectivity

**DOI:** 10.1002/anie.202101180

**Published:** 2021-05-10

**Authors:** Alex J. Plajer, Charlotte K. Williams

**Affiliations:** ^1^ Oxford Chemistry Chemical Research Laboratory 12 Mansfield Road Oxford OX1 3TA UK

**Keywords:** CO_2_ copolymerization, multimetallic catalysis, ring-opening copolymerization, ring-opening polymerization, switchable catalysis

## Abstract

A challenge in polymer synthesis using CO_2_ is to precisely control CO_2_ placement in the backbone and chain end groups. Here, a new catalyst class delivers unusual selectivity and is self‐switched between different polymerization cycles to construct specific sequences and desirable chain‐end chemistries. The best catalyst is a trinuclear dizinc(II)sodium(I) complex and it functions without additives or co‐catalysts. It shows excellent rates across different ring‐opening (co)polymerization catalytic cycles and allows precise control of CO_2_ incorporation within polyesters and polyethers, thereby allowing access to new polymer chemistries without requiring esoteric monomers, multi‐reactor processes or complex post‐polymerization procedures. The structures, kinetics and mechanisms of the catalysts are investigated, providing evidence for intermediate speciation and uncovering the factors governing structure and composition and thereby guiding future catalyst design.

## Graphical abstract

A dizinc‐sodium catalyst has been synthesized which allows precise control of CO_2_ incorporation within poly(carbonates), ‐esters, and ‐ethers. The catalyst selectively switches between three distinct catalytic cycles; kinetics and structure–activity studies inform the mechanisms of switchable polymerization catalysis.

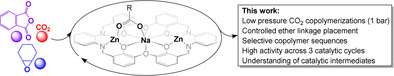

## Introduction

Carbon dioxide utilization is essential to add value to and recycle wastes; when it is effectively coupled with sequestration and storage it also has potential for significant reductions in greenhouse gas emissions.[^1^, ^2^, ^3^] Society urgently needs these utilization technologies but the fundamental science is under‐developed and lacks viable products. One effective and genuine utilization is the catalytic ring‐opening copolymerization (ROCOP) of carbon dioxide and epoxides.[^4^, ^5^, ^6^, ^7^, ^8^] This process yields aliphatic polycarbonates which are applied either as low‐molecular weight polyols in polyurethanes, coatings, and surfactants production or as high‐molecular weight plastics or elastomers.^[9]^ These products are being commercialized but opportunities remain to improve catalytic activity, selectivity, product structural diversity and to fully understand catalytic cycle intermediates. Most homogenous catalysts are highly selective for carbon dioxide/epoxide ROCOP and conditions are optimized to produce very few ether linkages.[^10^, ^11^, ^12^, ^13^] Yet, catalysis controlling both the location and overall fraction of CO_2_ taken up could effectively moderate polymer physical‐chemical properties without requiring any changes to the monomers, polymerization processes or catalysts. Such controllable catalysis should also allow other monomers, such as anhydrides, which are known to copolymerize with epoxides, to be efficiently localized within the polymer backbone.[^14^, ^15^] Chains featuring oligoether end groups would be especially desirable to overcome a problematic instability of carbon dioxide derived polycarbonates. These polycarbonates, in contact with residual catalyst, are easily depolymerized under conditions including removal of carbon dioxide gas atmosphere, heating, exposure to base and/or metal salts.[^16^, ^17^, ^18^, ^19^, ^20^] The chemical instability necessitates well‐timed process purifications and complete catalyst removal prior to polymer processing and application.[^21^, ^22^] Oligoether groups are much more stable but installing them onto polycarbonate chain ends is difficult due to the high nucleophilicity of the propagating metal alkoxide species which preferentially attacks the polycarbonate chain by back‐biting reactions over sequential epoxide enchainment.^[23]^ Block polyethers are usually prepared in two steps: firstly by epoxide ring‐opening polymerization and purification and, secondly, by use of the polyethers as chain transfer agents in epoxide/carbon dioxide ROCOP; unfortunately, such a strategy cannot place oligoethers at both chain ends.[^24^, ^25^, ^26^] An attractive alternative would be to develop catalysis with an intrinsic mechanistic switch between either epoxide ROP or epoxide/carbon dioxide ROCOP, but so far this has not proved possible.[^27^, ^28^, ^29^, ^30^]

Dinuclear metal complexes can be highly effective in CO_2_/epoxide ROCOP catalysis.[^31^, ^32^, ^33^, ^34^] The best catalysts are highly active under low CO_2_ pressures obviating the need of specialist reactors and operating without additives. Cyclohexene oxide (CHO)/carbon dioxide ROCOP catalysis is an important benchmark and the product poly(cyclohexene carbonate) (PCHC) has an attractive high glass transition temperature, high Young's modulus and structural rigidity complementary to bio‐based aliphatic polyesters. So far, none of the highly active metal catalysts can switch into CHO ring‐opening polymerization (ROP) and, therefore, it is difficult to control the quantity and placement of CO_2_ within the polymer chain.[^35^, ^36^] To moderate these catalysts also requires better understanding of the polymerization mechanism, catalytic intermediate speciation, and factors mediating activity and selectivity.

## Results and Discussion

To selectively catalyze both epoxide ROP and epoxide/carbon dioxide ROCOP, heterometallic catalysts with distinctive roles for each metal in the catalytic cycles were targeted.^[32]^ Previous dinuclear catalysts almost always have rate laws dependent upon both catalyst and cyclohexene oxide concentrations. It was hypothesized that increasing the local concentration of metal coordinated epoxide, with respect to metal carbonate intermediate, could accelerate rates and control ether linkage formation. Synergic heterometallic complexes, featuring Zn^II^ and Na^I^, were targeted owing to the precedent for Zn^II^ in high‐performance ROCOP catalysis and for Na^I^ in epoxide ROP, with additional benefits due to the latter's abundance and low cost.[^37^, ^38^, ^39^, ^40^] The trinucleating ligand H_2_ **L**, developed by Akine et al., was selected since it features two bis(phenoxy)diimine binding pockets, that is, salen‐type coordination chemistry, suitable for Zn^II^ and the central six O‐donors match with the coordination chemistry of sodium.[^41^, ^42^]

**Figure 1:**
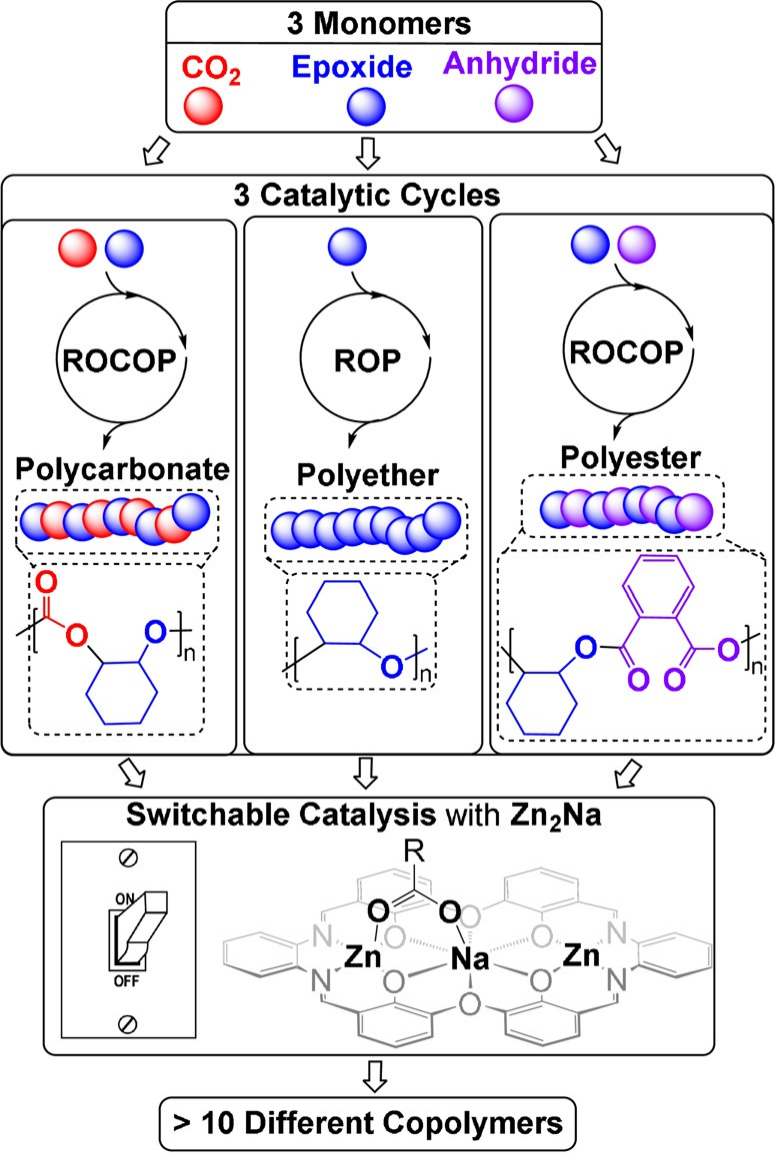
Monomers and switchable catalytic cycles and the new trinuclear catalyst **Zn_2_Na**.

The target **Zn_2_Na** catalyst was prepared in 96 % isolated yield by the simultaneous metalation of H_2_ **L** by reaction with [Zn(OAc)_2_⋅(H_2_O)_2_] and NaCO_2_C_6_H_4_CF_3_ (Figure 1). **Zn_2_Na** was fully characterized and analytical purity confirmed (Supporting Information, Section S2). With pure **Zn_2_Na** in hand, its performance in CO_2_/CHO ROCOP was investigated using conditions only successful with high performance catalysts (1:4000 **Zn_2_Na**:CHO, neat CHO, 80–120 °C, 1 bar CO_2_, Table 1; Supporting Information, Section S3.1).

**Table 1:** Data for copolymerizations of CHO and CO_2_ using **Zn_2_Na**.

Reaction Temp. [°C]	Polym. Selectivity [%]^[b]^	Carbonate: Ether Selectivity [%]^[c]^	Cat. TON^[d]^	Cat. TOF [h^−1^]^[e]^	Polymer M_n_ [kg mol^−1^] (*Ð*)^[f]^
80^[a]^	99	>95:<5	920	75	2.42 (1.20)
90^a^	98	90:10	2400	270	6.86 (1.24)
100^[a]^	97	86:14	1960	478	5.61 (1.29)
110^[a]^	96	82:18	2120	578	7.12 (1.26)
120^[a]^	94	67:33	2000	956	5.12 (1.24)
80^[g]^	99	41:59	1200	200	2.38 (1.22)
120^[h]^	96	>95:<5	1360	2900	5.35 (1.16)

[a] Copolymerization conditions: 0.025 mol % catalyst loading (1:4000), 20 equiv 1,2‐cyclohexane diol (CHD), 1 bar CO_2_, CHO neat (9.99 M). Polymerizations were stopped after 20 h or once conversion plateaued (Supporting Information, Section S3). [b] Determined by comparison of the relative integrals, in the normalized ^1^H NMR spectrum, of resonances due to polymer (δ 4.65 ppm, 3.45 ppm) and cyclic carbonate (δ 4.00 ppm) (Supporting Information, Figure S16). [c] Determined by comparison of the relative integrals, in the normalized the ^1^H NMR spectrum, of resonances due to carbonate (δ 4.65 ppm) and ether (δ 3.45 ppm) linkages (Supporting Information, Figure S16). [d] Turnover number (TON), number of moles of CHO consumed per mole of catalyst. [e] Turnover frequency (TOF) determined from initial rates analysis by in situ ATR‐IR spectroscopy (typically 5–15 % conversion) as TON/time. [f] Determined by GPC (gel permeation chromatography) measurements conducted in THF, using narrow MW polystyrene standards to calibrate the instrument (Supporting Information, Section S1); *Ð*=M_w_/M_n_. [g] Polymerization conducted using unpurified gas mixture comprising 0.5 bar CO_2_ and 0.5 bar N_2_ with otherwise analogous conditions. [h] Polymerization conducted using 20 bar CO_2_ pressure in a stainless‐steel reactor with mechanical stirring (Supporting Information, Section S1).

**Figure 2:**
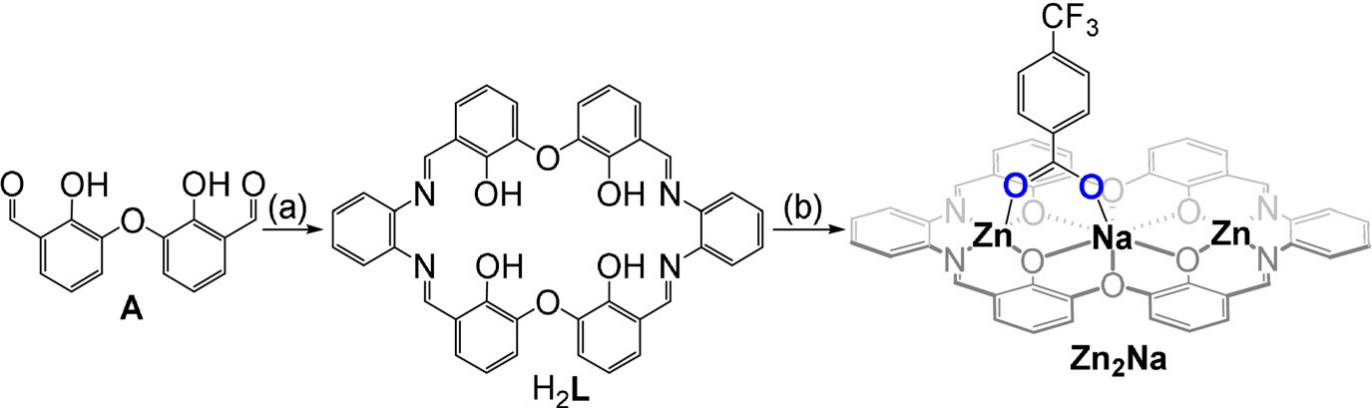
Synthesis of the **Zn_2_Na** from H_2_ **L**. a) 1 equiv 1,2‐diaminobenzene, CH_3_CN, room temperature, 7 d, 55 %. b) 2 equiv [Zn(OAc)_2_⋅(H_2_O)_2_], 1 equiv NaCO_2_C_6_H_4_CF_3_, 1:1 DCM:MeOH, room temperature, 5 min, 96 %. **A** obtained as previously described by Akine et al (Supporting Information, Section S1).

Under these conditions, **Zn_2_Na** showed excellent activity and high polymer selectivity from 80–120 °C. As the temperature increased, the polymer composition changed from almost entirely carbonate linkages (<5 % ether) to polycarbonates featuring random ether linkages (33 % ether). Such facile ether linkage control is unexpected for low pressure catalysts which rely on high carbonate linkage stability to maximize polymer selectivity (see below). Compared to other low pressure catalysts (Supporting Information, Section S3.2), **Zn_2_Na** showed excellent absolute polymer productivity (TON) and activity (TOF) and tolerated impressive low loading (1:4000, catalyst:CHO). Its performance is particularly exciting as it comprises inexpensive metals, obviates co‐catalyst requirements and highlights how the inclusion of sodium enhances the catalysis. The controllable ether linkage selectivity was exploited by changing the polymerization temperature during a run to produce a gradient copolymer (Figure 2; Supporting Information, Section S3.3). Polymerizations were monitored continually using in situ ATR‐IR spectroscopy which clearly identifies compositional selectivity: by increasing the reaction temperature, from 80 to 120 °C, a concomitant sharp rise in ether linkage formation occurs. The production of ether linkages was also moderated by the carbon dioxide pressure (Supporting Information, Section S3.1), for example at 120 °C and 20 bar pressure the selectivity for PCHC was essentially quantitative, whilst at the same temperature but with 1 bar CO_2_ almost one third ether linkages were produced. Under both high temperature and pressure conditions, the catalyst showed a very high TOF >2900 h^−1^_._ It was also active at carbon dioxide pressures below 1 bar, showing increased ether linkage formation. For example, a mixture of 0.5 bar CO_2_ and 0.5 bar N_2_ results in 41 % carbonate and 59 % ether linkages. Remarkably, under these conditions it still maintained an impressive activity, with TOF >200 h^−1^ at 80 °C. The high activity of **Zn_2_Na** at sub‐1 bar CO_2_ pressure is very unusual; most catalysts appear to fail under these conditions and a rare report of a dizinc catalyst showed a significant activity and selectivity reduction in this regime.^[43]^ In all cases the maximum CHO conversion during CO_2_ ROCOP and switchable catalysis was found to be <60 % which we attribute at least in part to the increase in viscosity during polymerizations conducted in glassware and using magnetic stirring.

**Figure 3:**
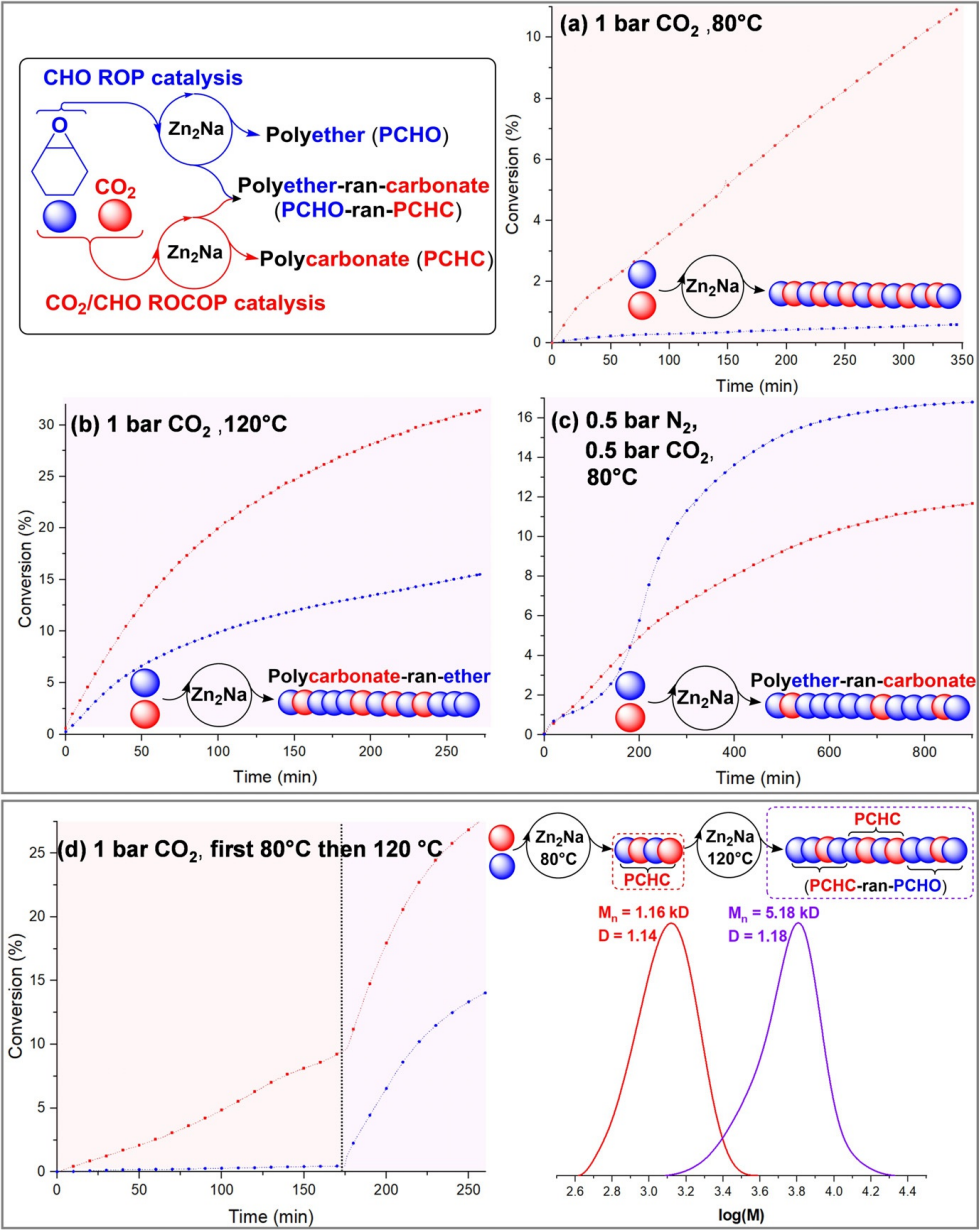
a)–c) Illustration of CHO ROP and CHO/CO_2_ ROCOP using **Zn_2_Na**. Initial monomer conversion versus time plots determined using in situ IR spectroscopy, monitoring PCHC formation via its carbonate absorption at 1744 cm^−1^ and PCHO formation via its absorption at 1089 cm^−1^. Conversions referenced using ^1^H NMR spectra of aliquots. d) Gradient polymer formation using an in situ temperature switch, from 80 °C to 120 °C, at 175 min and the resulting GPC traces of aliquots removed. Copolymerization conditions: 0.025 mol % catalyst loading (1:4000), 20 equiv 1,2‐cyclohexane diol (CHD), CHO neat (9.99 M).

**Zn_2_Na** was also a stand‐alone catalyst for CHO ROP to form poly(cyclohexene oxide) PCHO (Figure 3; Supporting Information, Sections S4 and S5). In situ ATR‐IR spectroscopy showed that CHO ROP occurred significantly faster than CO_2_/CHO ROCOP, with TOF values from 454–1477 h^−1^ at temperatures from 80–120 °C (TOF=75–602 h^−1^ for the equivalent CHO/CO_2_ ROCOP). The CHO ROP was well controlled as evidenced by linear increases to PCHO molar mass and its narrow dispersity (*Ð*>1.3). In the case of both ROP and ROCOP, the majority of chains are initiated from the CHD chain‐transfer agent while a minority (<5 %) is initiated from the trifluorobenzoate co‐ligand of **Zn_2_Na**.

Having established that **Zn_2_Na** catalyzes both CHO ROP and CHO/CO_2_ ROCOP, next attention turned to how to select for a particular catalytic cycle; that is, switchable catalysis. The CHO/CO_2_ ROCOP was completely and immediately stopped by changing the gas atmosphere from carbon dioxide to nitrogen and, at the same time, this switch resulted in an unusual rate acceleration as CHO ROP occurs faster than its CO_2_ ROCOP (Supporting Information, Section S7.1). This result was surprising because other catalysts undergo PCHC degradation by back‐biting reactions to cyclic carbonate when the carbon dioxide atmosphere is removed (see below).^[23]^ In contrast, **Zn_2_Na** undergoes negligible back‐biting (ca. 1 % cyclic carbonate formation). Because the polymerizations are initiated from 1,2‐cyclohexane diol, chains are telechelic and hence the CHO ROP phase results in the efficient installation of oligoethers at both chain ends. It was also possible to prepare more complex sequences by introducing carbon dioxide into the polymerization solution at pre‐determined time intervals, that is, reversible switching. As soon as carbon dioxide was added the catalysis underwent an immediate switch back to CO_2_/CHO ROCOP. Lastly, the reverse end‐capping of a central PCHO block with two outer PCHC blocks was feasible via switching from a nitrogen to carbon dioxide gas atmosphere (Supporting Information, Sections S7.2 and S7.3).

Intrigued by the facility to control ether linkage content in carbon dioxide derived polymers, **Zn_2_Na** was tested in mechanistically related epoxide/anhydride ROCOP. Such controlled copolymerizations produce polyesters showing structures that would be impossible or very difficult to access using lactone ROP.^[14]^ Phthalic anhydride (PA) was selected as a model co‐monomer since it is a common ROCOP activity benchmark and is a commercial product already used at scale in other polymer manufacturing.^[15]^ Using **Zn_2_Na** for PA/CHO ROCOP, in neat CHO, results in the selective formation of poly(cyclohexylene‐*alt*‐phthalate) (PCHPE) without ether linkages. The polyester shows a narrow, monomodal molecular weight distribution indicative of good control. Furthermore, exposure of the catalyst to ternary mixtures of CO_2_/PA/CHO results in the selective formation of polyester, as has been observed for other terpolymerization catalysts.[^44^, ^45^] In terms of catalytic activity, the absolute performance of **Zn_2_Na** is high (TOF=173–1093 h^−1^, 100–130 °C), placing it amongst the most active catalysts in this field (Supporting Information, Section S6).^[14]^

**Figure 4:**
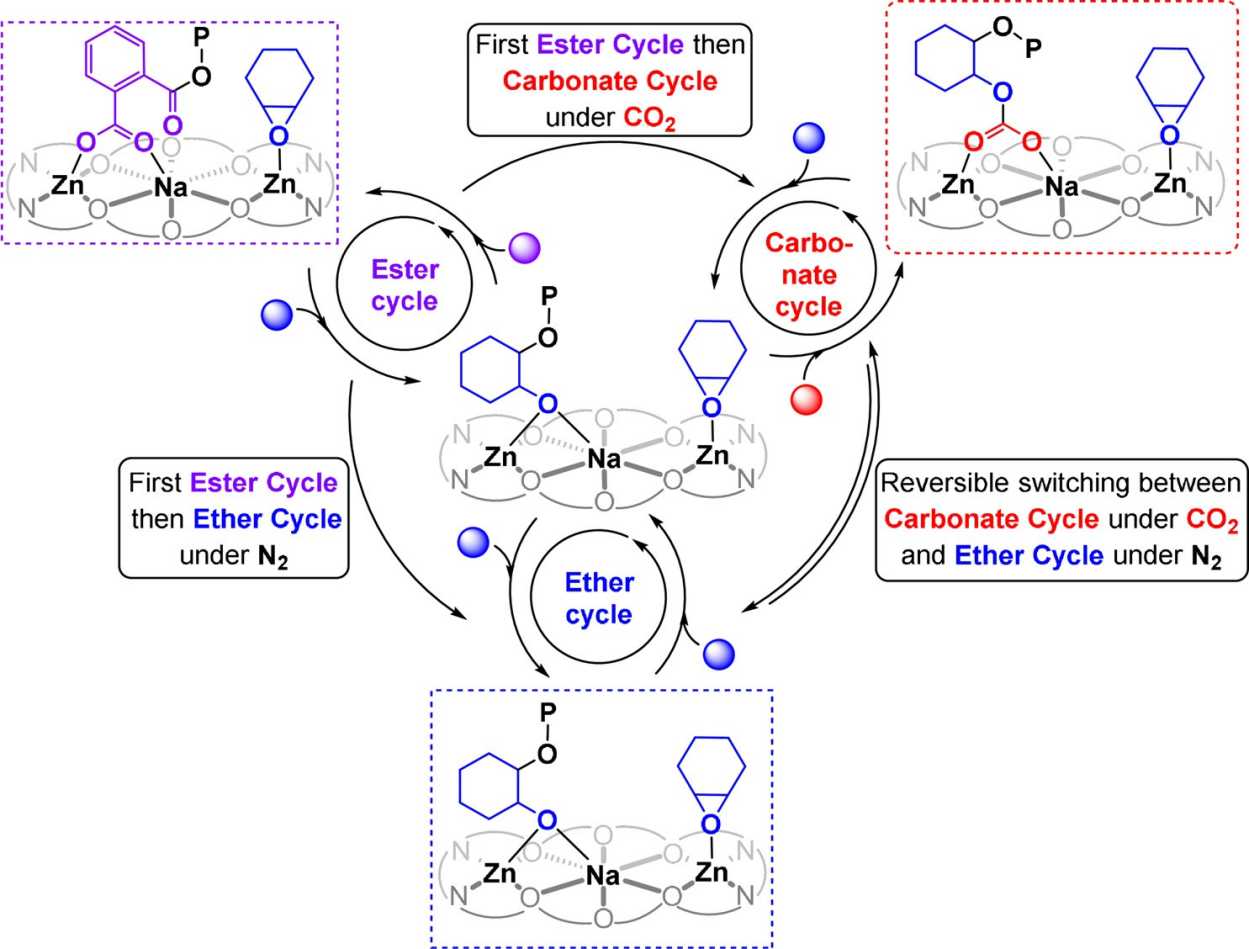
Illustration of the different catalytic cycles accessed by **Zn_2_Na** and the sequences feasible in switchable catalysis.

Next, **Zn_2_Na** was investigated for switchable catalysis using mixtures of PA, CHO and CO_2_ (Figure 4). Mixtures of CO_2,_ excess CHO and PA resulted in first PA/CHO ROCOP until the anhydride was completely consumed, followed by CO_2_/CHO ROCOP (Supporting Information, Section S7.4). Changing the gas atmosphere at this stage, from CO_2_ to N_2_, resulted in immediate cessation of CO_2_/CHO ROCOP and the onset of CHO ROP leading to production of polyether chain ends. As proof of potential, the gas atmosphere was switched back to carbon dioxide to produce a second polycarbonate sequence (Supporting Information, Section S7.5). The polymerization of excess CHO and PA, under N_2_, selectively forms polyester (PA/CHO ROCOP) but, once all PA is consumed, CHO ROP starts. At any point during the ether ROP stage, carbon dioxide can be added which immediately switches the catalysis into the CHO/CO_2_ ROCOP cycle and forms polycarbonate linkages (Supporting Information, Section S7.6). Consecutive enchainment of these different linkages (ester, carbonate, ether) into single block‐copolymers was confirmed using a range of analytical techniques (Supporting Information, Section S7.6). It should be noted that owing to the comparatively low degrees of polymerizations achieved, the monomer sequencing via switchable catalysis does not substantively influence material properties such as glass transition temperature or solubility.

Previously, dizinc switchable catalytic selectivity was rationalized by different transition state barriers and linkage stabilities, as modelled using DFT.^[45]^ The conclusion was that selectivity depended upon both kinetic and thermodynamic factors. This rationale and predictable monomer enchainment rules have subsequently proven successful for predicting behavior of other metallic/bimetallic catalysts, organocatalysts and for a range of other monomers.[^27^, ^28^, ^30^, ^46^] Nonetheless, so far the hypothesis lacks clear experimental evidence for the proposed intermediates or investigations of intermediate reactivity. This lack of evidence arises from the sensitivity (to moisture), paramagnetism and/or fluxionality of key intermediates, as well as uncertainty regarding the number of chains growing per catalyst molecule and, in cases where co‐catalyst is necessary, the presence of multiple competitive initiation and propagation species.[^12^, ^31^, ^32^] **Zn_2_Na** could be regarded as privileged in this regard since it features just one initiating group (benzoate) per complex and functions without co‐catalyst. To better understand its special performance and selectivity, Arrhenius analyses of each of the separate polymerization catalyses was conducted (Supporting Information, Section S8.1). The activation energies decrease in the order PA/CHO ROCOP *E*_A_=(76.43±3.71) kJ mol^−1^ > CO_2_/CHO ROCOP *E*_A_=(62.96±11.90) kJ mol^−1^ > CHO ROP *E*_A_=(37.60±6.17) kJ mol^−1^. Somewhat counter‐intuitively, the monomer selectivity from mixtures follows the opposite sequence: ester > carbonate > ether (that is, PA > CO_2_ > CHO) attributed to the potential for reversible monomer insertions, linkage stability and pre‐rate limiting step rates. These findings are key to understanding switchable polymerization catalysts and, here, unusually the enchainment of ether linkages is feasible.[^27^, ^28^, ^30^, ^46^]

It was also observed that during either PA/CHO or CO_2_/CHO ROCOP the reaction solutions were bright orange but during CHO ROP the color significantly darkened to brown. Thus, this catalyst allows for a colorimetric indicator of switching and because color changes were immediate and reversible, the reaction color provides a real‐time measure of both specific monomer enchainment and of intermediate speciation.^[47]^ UV/Vis spectroscopy of the CHO ROP solutions revealed the brown color originated from a new absorption at 559 nm attributed to the metal‐alkoxide intermediate (Supporting Information, Section S8.2). The alkoxide speciation was confirmed by reaction of **Zn_2_** with NaO^t^Bu resulting in brown solutions, with a very similar UV/Vis absorption at 552 nm. The absorption is tentatively assigned to electronic transitions associated with the Zn^II^–Schiff base (imine) moiety, indicating that the alkoxide intermediate likely bridges between the Na^I^ and Zn^II^ sites.

Exposure of brown **RO‐Zn_2_Na** (from reaction of **Zn_2_Na** with 4000 equiv CHO at 100 °C), to 1 atm ^13^CO_2_ (at room‐temperature in CHO), led to an immediate color change to orange and complete disappearance of the absorption at 559 nm, both observations correspond closely to the spectroscopic features observed during CO_2_/CHO ROCOP catalysis. ^13^C{^1^H} NMR spectroscopy unambiguously confirms the formation of the carbonate group, with a resonance at 160.3 ppm for intermediate **ROCO_2_‐Zn_2_Na** (Supporting Information, Section S8.3). The speciation of **ROCO_2_‐Zn_2_Na** was further confirmed by the independent synthesis of a model carbonate complex featuring a OCO_2_^t^Bu co‐ligand. Adding stoichiometric amounts of PA, at room temperature, to a brown solution of **RO‐Zn_2_Na** led to the immediate formation of a bright orange solution, proposed as the carboxylate intermediate **ArCO_2_‐Zn_2_Na**. The UV/Vis spectrum of this orange solution was identical to that observed for the catalyst present during PA/CHO ROCOP. The ^1^H NMR spectrum of **ArCO_2_‐Zn_2_Na** is also very similar, but with somewhat broader resonances, to the spectrum of the starting carboxylate complex **Zn_2_Na**. Additionally, its ^13^C NMR spectrum reveals formation of two different phthalate signals at 169.6 and 164.6 ppm, corresponding to the carbonyl groups closer and further from the metals, respectively.

These experiments, together with the polymerization kinetic analyses, provide much needed support for the switchable catalysis mechanism and rationalize the novel monomer selectivity. The mechanism is underpinned by different catalytic cycles and intermediates, some of which can bridge between the various polymerizations: during CO_2_/CHO ROCOP the resting state is a carbonate species, that is, **ROCO_2_‐Zn_2_Na**, during PA/CHO ROCOP it is a carboxylate complex, that is, **ArCO_2_‐Zn_2_Na**, and during CHO ROP it is an alkoxide intermediate **RO‐Zn_2_Na**. The rate determining step in each catalytic cycle appears to be epoxide ring‐opening (CHO) and whilst insertion of PA or CO_2_ into the **RO‐Zn_2_Na** intermediate occurs readily at room temperature, insertion of CHO into the **ROCO_2_‐Zn_2_Na** or **RCO_2_‐Zn_2_Na** intermediates does not occur below 80 °C or 100 °C, respectively, as at lower temperatures the barriers to ROCOP are too high (see above).

The pre‐rate determining step involves insertion of different monomers into the metal alkoxide intermediate: phthalic anhydride, carbon dioxide or epoxide. Carbon dioxide insertion appears to be diffusion limited, based on related insertion kinetics during CO_2_/CHO ROCOP, and occurs at least 10^8^ times faster than epoxide insertion.^[35]^ Hence, the partial CO_2_ incorporation under some conditions during CO_2_/CHO ROCOP cannot be kinetically controlled and must be the consequence of a thermodynamic equilibrium. Van't Hoff analysis of the equilibrium: [**RO‐Zn_2_Na**]+[CO_2_]⇄[**ROCO_2_**‐**Zn_2_Na**] yields Δ*H*=−(88.25±11.45) kJ mol^−1^, Δ*S*=(189.37±39.73) J mol^−1^ K^−1^, consistent with endotropic, but exothermic, fixation of CO_2_ (Supporting Information, Section S8.1). This translates to a controllable ratio of [**ROCO_2_‐Zn_2_Na**]:[**RO‐Zn_2_Na**] from 15:1 at 80 °C, to 2:1 at 120 °C (that is, a change from 99 % to 79 % concentration of **ROCO_2_‐Zn_2_Na** in the catalytic resting state).

**Figure 5:**
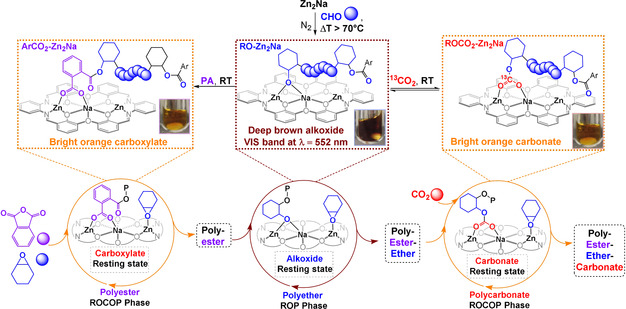
Identification of the catalyst intermediates during CHO/CO_2_ ROCOP, CHO/PA ROCOP and CHO ROP. Stoichiometric and in situ measurements confirm the speciation as CHO ROP=**RO‐Zn_2_Na**, CO_2_/CHO ROCOP=**ROCO_2_‐Zn_2_Na** and CHO/PA ROCOP=**ArOCO_2_‐Zn_2_Na**.

Thus, carbon dioxide insertion is rapid and reversible under the conditions of these polymerizations. On the other hand, the data indicate that the insertion of PA into the metal alkoxide intermediate is irreversible. Monitoring PA consumption kinetics indicates that it follows a zeroth order, consistent with rapid yet irreversible insertion of phthalic anhydride (Supporting Information, Sections S6 and S7.4–S7.6). Over the entire temperature range of these experiments (100–130 °C), the polyester selectivity remains quantitative but the equivalent carbon dioxide insertion decreases with increasing temperature. Together these observations suggest that PA insertion into **RO‐Zn_2_Na** is orders of magnitude faster than epoxide insertion and is irreversible in the investigated temperature interval. Importantly, this irreversible reactivity explains why PA/CHO ROCOP occurs selectively before CO_2_/CHO ROCOP when all three monomers are present in mixtures (Figure 5).

**Figure 6:**
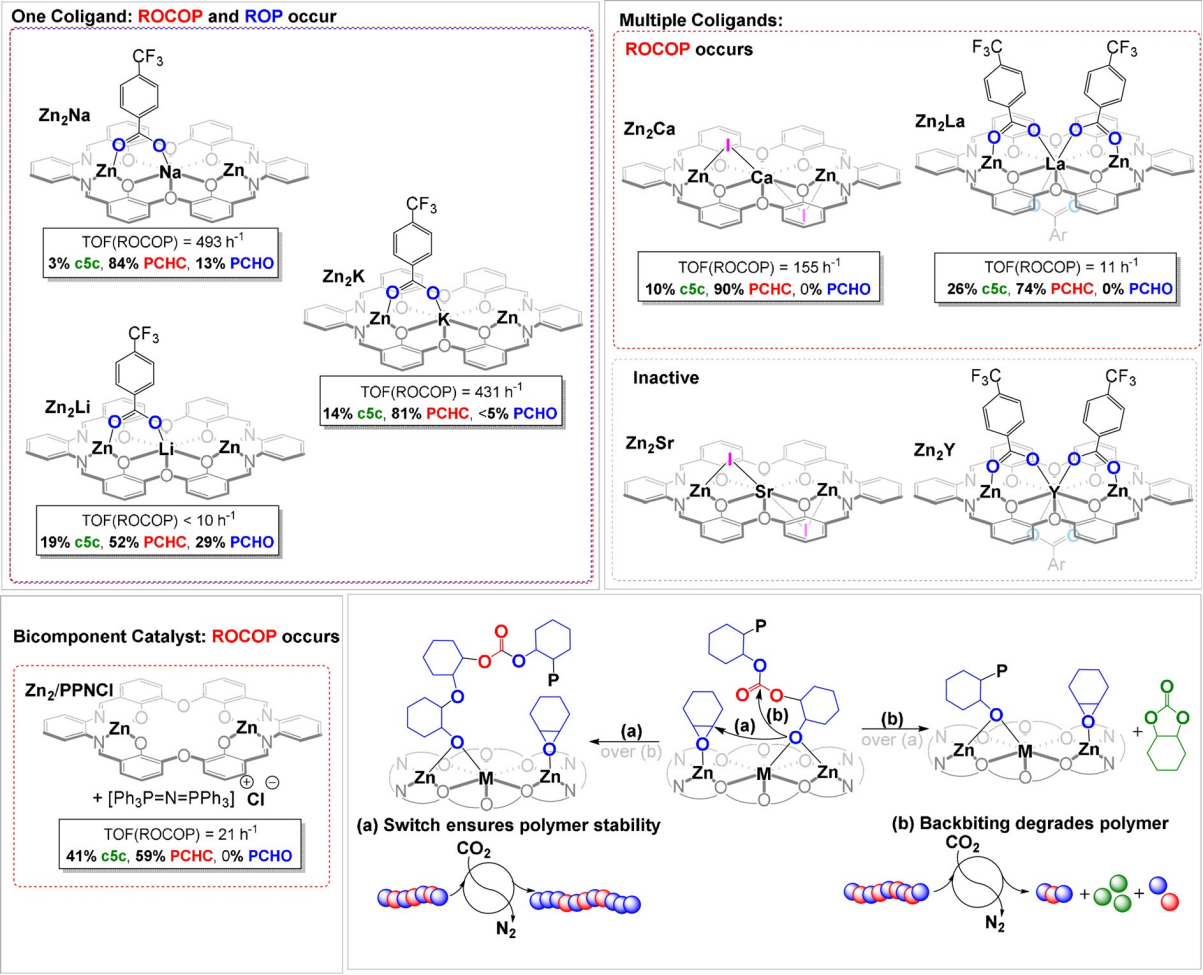
Comparison of the catalytic properties of **Zn_2_Na/Li/K**; **Zn_2_Ca/Sr**, **Zn_2_La/Y** and **Zn_2_**. Polymerization Conditions: 0.025 mol % catalyst (1:4000), 20 equiv 1,2‐cyclohexane diol (CHD), 1 bar CO_2_, CHO neat (9.99 M). Note that specific activity (TOF) is reported, that is, per initiating group, to account for differences in metal oxidation state. Differences in co‐ligand chemistries (iodide vs. benzoate) do not affect activity or selectivity which are determined only during propagation (continual ATR‐IR spectroscopy).

To examine the apparently special role of the Na^I^ centre in **Zn_2_Na**, the synthesis of a series of other complexes was undertaken (Figure 6). The series comprises Group 1 metals (Li, Na, K), Group 2 congeners (Ca, Sr) and two representative Group 3 centers (Y, La): all complexes were tested under common polymerization conditions (Supporting Information, Sections S9–S15). These complexes allow an answer to the long‐standing question of how many co‐ligands are responsible for initiation in multimetallic catalysts.^[48]^ Using in situ ^19^F NMR spectroscopy to characterize **Zn_2_La** during polymerization reveals that all three 4‐triflouromethyl‐benzoate groups initiate (Supporting Information, Section S13). After initiation, the inorganic benzoate group is transformed into an organic ester which leads to a reproducible change in chemical shift value of ca 1 ppm. The results of this experiment indicate that comparisons of catalytic activity should be made per initiator to accurately account for factors underpinning high activity and selectivity.

In terms of catalytic performances, **Zn_2_La** forms only polycarbonate, without any ether linkages, and shows significantly lower specific activity (TOF=11 h^−1^ per initiator) and reduced polymer selectivity (74 %) than **Zn_2_Na**. The calcium catalyst, **Zn_2_Ca** also selectively formed PCHC, without ether linkages, (TOF 155 h^−1^ per initiator, polymer selectivity 90 %), while the strontium and yttrium derivatives (**Zn_2_Sr**, **Zn_2_Y**) were inactive. The activity of **Zn_2_Ca** (TOF 427 h^−1^ per initiator) for PA/CHO ROCOP is notably high, especially considering it operates without co‐catalyst (Supporting Information, Sections S6 and S11). The sodium in **Zn_2_Na** appears to be essential both in delivering the unusual ether linkage selectivity and in accelerating activity during CO_2_/CHO ROCOP. Nearly two decades ago, Darensbourg and co‐workers proposed that vacant metal coordination sites might account for ether linkage formation during CHO/CO_2_ ROCOP and their prescient observation is substantiated for this series of complexes.[^49^, ^50^, ^51^] **Zn_2_Ca**, **Zn_2_La**, **Zn_2_Sr**, and **Zn_2_Y** contain more coligands than **Zn_2_Na** and these co‐ligands likely bridge between the Zn^II^ centres and reduce the coordinative vacancies. The Group 1 congeners, **Zn_2_K** (TOF 431 h^−1^) and **Zn_2_Li** (TOF <10 h^−1^), both have a single co‐ligand and hence offer at least four vacant sites for epoxide coordination. Both these catalysts show both CHO ROP and CHO/CO_2_ ROCOP, albeit with lower activity and selectivity than the sodium analogue. The decrease in polymer selectivity may originate from less stable alkali metal coordination as **L** was previously found to be Na^I^ ion selective.^[41]^ Changing the alkali metal from sodium may weaken the alkoxide‐catalyst association and favor polycarbonate backbiting over polyether formation. In agreement with this hypothesis, a bicomponent system comprising of **Zn_2_**+**PPNCl** produces 49 % cyclic carbonate (c5c) and the residual polymer is just PCHC (TOF=21 h^−1^).

A final observation was that activity at pressures below 1 bar CO_2_, important in the context of practical CCU, is also contingent upon metal selection. Under 0.5 bar CO_2_ partial pressure, **Zn_2_Ca** and **Zn_2_La** showed massively decreased activity and polymer selectivity, whereas, as noted earlier, **Zn_2_Na** retains both high activity and selectivity. These differing performances are rationalized by the switchable catalysis mechanisms. Under low pressures, the carbon dioxide concentration reduces which changes the equilibrium between metal carbonate and alkoxide intermediates. The combined effect of shifting the equilibrium significantly towards alkoxide and where that species cannot react to form ether linkages (through epoxide ROP), results in significant back‐biting of the polycarbonate to form cyclic carbonate. This is clearly exemplified by **Zn_2_Ca** and **Zn_2_La** which show efficient catalytic decomposition of PCHC, at 120 °C under N_2_ (Supporting Information, Sections S11 and S13). In contrast, PCHC which is endcapped with oligo(CHO) groups retained its stability and composition under identical conditions either in the presence of catalyst or upon removal of CO_2_ (Supporting Information, Section S7.1). These findings clearly demonstrate the benefits of oligoether end‐capping of polycarbonate chains and underscore the importance of sodium in these high activity trimetallic catalysts.

## Conclusion

A highly active heterometallic sodium‐dizinc catalyst showed high activity for three different polymerizations: 1) The ring‐opening copolymerization of CO_2_/cyclohexene oxide; 2) The ring‐opening copolymerization of phthalic anhydride/cyclohexene oxide; and 3) The ring‐opening polymerization of cyclohexene oxide. The catalyst is unusual because it allows for control over the placement and formation of poly(carbonate), ‐ester, and ‐ether linkages, the latter are beneficial end‐groups for product stability. It is easily switched between three different catalytic cycles providing a straightforward and highly practical route to make new polymer compositions. The catalyst shows high absolute activity and maintains both its activity and selectivity under sub‐atmospheric CO_2_ pressure; conditions which may be relevant to carbon dioxide utilization scenarios. Detailed investigations of polymerization kinetics, catalyst speciation and thermodynamics underpin a mechanism and allow for new insights into the factors governing control and selectivity in switchable polymerization catalyses. Monomer selectivity, from mixtures, is governed by the insertion reaction thermodynamics and equilibria. The best catalyst features inexpensive and abundant sodium within a ligand scaffold incorporating two zinc metals; vacant coordination sites adjacent to the metal‐nucleophile and tight co‐ligand binding are key to reducing barriers to epoxide ROP thereby enabling ether linkage incorporation. These findings should stimulate research into new catalysts using other metals and ligands; the continued exploration of light Group 1 metals is certainly warranted on the basis of performances, costs and abundance. The switchable rules established here should apply to other epoxides, anhydrides, heterocumulenes, heterocycles and even lactones: many of these monomers are commercially available and a significant number are, or could easily be, bio‐based. Thus, this work sign‐posts routes to other new carbon dioxide and bio‐based polymers.

## Conflict of interest

C.K.W. is a director of econic technologies.

## Supporting information

As a service to our authors and readers, this journal provides supporting information supplied by the authors. Such materials are peer reviewed and may be re‐organized for online delivery, but are not copy‐edited or typeset. Technical support issues arising from supporting information (other than missing files) should be addressed to the authors.

Supplementary
