# Supplementary material for: Heterotrimetallic Carbon Dioxide Copolymerization and Switchable Catalysts: Sodium is the Key to High Activity and Unusual Selectivity
Source: Angew Chem Int Ed Engl. 2021 May 10;60(24):13372–9. doi: 10.1002/anie.202101180 (PMC8251569; doi:10.1002/anie.202101180)
Supplement: Supplementary file 1 — Supplementary [file ANIE-60-13372-s001.pdf]

Angewandte Chemie
Eine Zeitschrift der Gesellschaft Deutscher Chemiker

Supporting Information

Heterotrimetallic Carbon Dioxide Copolymerization and Switchable Catalysts: Sodium is the Key to High Activity and Unusual Selectivity

*Alex J. Plajer and Charlotte K. Williams**

anie_202101180_sm_miscellaneous_information.pdf

Contents

Section S1: Methods ... 2

Section S2: Synthesis and characterisation of Zn_2Na ... 4

Section S3: Kinetic data for CO_2/CHO ROCOP ... 8

Section S3.1: Kinetic data for CO_2/CHO ROCOP at different temperatures and pressures ... 8

Section S3.2: Comparison of literature know catalysts to Zn_2Na ... 17

Section S3.3: *In situ* Temperature switching and polymerisation without CHD ... 18

Section S4: CHO ROP ... 21

Section S5: Synthesis and characterisation of Zn_2Na_{BArF} ... 22

Section S6: PA/CHO ROCOP ... 26

Section S7: Switchable Catalysis ... 27

Section S7.1: CO_2/CHO ROCOP to CHO ROP ... 27

Section S7.2: CHO ROP to CO_2/CHO ROCOP ... 29

Section S7.3: CO_2/CHO ROCOP to CHO ROP to CO_2/CHO ROCOP ... 31

Section S7.4: PA/CHO ROCOP to CO_2/CHO ROCOP to CHO ROP ... 33

Section S7.5: PA/CHO ROCOP to CO_2/CHO ROCOP to CHO ROP to CO_2/CHO ROCOP ... 35

Section S7.6: PA/CHO ROCOP to CHO ROP to CO_2/CHO ROCOP ... 38

Section S8: Mechanistic Investigations ... 41

Section S8.1: Arrhenius and van't Hoff analysis ... 41

Section S8.2: UV-Vis Spectroscopy ... 45

Section S8.3: NMR spectroscopy ... 47

Section S9: Synthesis of Zn_2K and its Polymerisation Kinetics ... 51

Section S10: Synthesis of Zn_2Li and its Polymerisation Kinetics ... 56

Section S11: Synthesis of Zn_2Ca and its Polymerisation Kinetics ... 59

Section S12: Synthesis of Zn_2Sr ... 63

Section S13: Synthesis of Zn_2La and its Polymerisation Kinetics ... 65

Section S14: Synthesis of Zn_2Y ... 71

Section S15: Polymerisation Kinetics of Zn_2+PPNCl ... 74

References ... 74

Section S1: Methods

Materials: Solvents and reagents were obtained from commercial sources and used as received unless stated otherwise. If "dried solvents" were used these were obtained from an SPS system, degassed by several freeze-pump-thaw cycles and further dried with 3 Å molecular sieves and stored under nitrogen. Cyclohexene oxide was dried over calcium hydride overnight and purified via fractional distillation prior to use and stored in an inert atmosphere. Phthalic anhydride was extracted with dry benzene, recrystallised from dry chloroform and sublimed at 100 °C and 0.05 bar. Commercial 1,2-cyclohexenediol was recrystallized from dry chloroform and dried under dynamic vacuum before use. 99.8% carbon dioxide supplied by BOC Ltd was dried by passing it through two drying columns (VICI Metronics carbon dioxide purifier) prior to use. Compound **A** and H_2**L** were obtained according to a procedure published by *Akine et al*.[1] The spectroscopic data was in agreement with the literature.

NMR spectra were recorded on a Bruker Advance 400 QNP or Bruker Avance 500 MHz cryo spectrometer. All spectra were recorded with external standards. Unambiguous assignments of NMR resonances were made on the basis of 2D NMR experiments.

UV-visible spectra were collected on a Varian Cary 50 UV spectrometer.

Gel permeation chromatography analysis was carried out on a a Shimadzu LC-20AD instrument equipped with two mixed bed PSS SDV linear S columns in series, THF as the eluent at a flow rate of 1mL/min and at 40 °C. Polymer molecular mass (M_n) was determined by comparison against narrow molecular mass polystyrene standards which were used to calibrate the instrument. Each polymer sample was dissolved in HPLC-grade THF (10 mg/mL) and filtered through a 0.20 µm porous filter frit prior to analysis.

High-resolution ESI mass spectra were obtained using a Thermofisher LTQ Orbitrap XL, by the EPSRC UK National Mass Spectrometry Facility at Swansea University.

Elemental analysis was obtained using a Perkin Elmer 240 Elemental Analyser, by Dr Nigel Howard from the elemental analysis lab at the University of Cambridge.

General Polymerization protocols

Example of low pressure epoxide copolymerization: A mixture of the catalyst (5.2 mg, 5.0 µmol, 1 equiv.), 1,2-cyclohexenediol, (if used, 11.7 mg. 100.0 µmol, 20 equiv.), phthalic anhydride (148.7 mg, 1.0 mmol, 200 equiv.) and cyclohexene oxide (2 mL, 20.0 mmol, 4000 equiv.) was added to a Schlenk tube, under nitrogen. If carbon dioxide was applied, the reaction was subjected to three vacuum/CO_2 cycles (pressure regulated at 1 bar CO_2 or 1 bar of a 1:1 mixture of CO_2 and N_2). If carbon dioxide was not used, the Schlenk tube was connected to vacuum/nitrogen line. In some cases, the Schlenk tube was fitted with a DiComp probe for *in situ*-ATR- IR spectroscopy (REACTIR) and submerged into an oil bath that was pre-heated to the appropriate temperature. At this point the REACTIR instrument was set to begin data-collection (t_0). Where a gas atmosphere switch was necessary, it was performed slowly using three vacuum/gas cycles at the given temperature. After the reaction was completed, the mixture was allowed to cool to room temperature and the crude product composition was analysed by NMR spectroscopy of an aliquot to determine the ratio of products (poly(cyclohexene carbonate) PCHC, poly(cyclohexene oxide) PCHO, cyclohexene carbonate c5c). The polymer was isolated by adding the concentrated polymerisation mixture (ca 0.5 mL, achieved by removing excess CHO under a stream of N_2) to 100 mL of acidified MeOH (10 µL concentrated HCl, per 100 mL MeOH) resulting in the precipitation of white product.

High pressure epoxide copolymerization: A suspension of catalyst (52.0 mg, 50.0 µmol, 1 equiv.), cyclohexene diol (117.0 mg, 1.0 mmol, 20 equiv.) in CHO (20.0 mL, 0.2 mol, 4000 equiv.) was injected

into a 100 mL Parr reactor, under a stream of dry CO_2. The reactor was also fitted with a DiComp sentinel probe, attached to an ATR-IR spectrometer, which allowed for continual monitoring of PCHC formation. The reactor was then pressurized with CO_2 to the target reaction pressure and allowed to reach the required temperature. Upon reaction completion, the reactor vessel was cooled to room temperature and depressurized; the product composition was analysed by NMR spectroscopy of an aliquot. The polymer was isolated by adding the concentrated polymerisation mixture (ca 5.0 mL, achieved by removing excess CHO under dynamic vacuum) to 500 mL of acidified MeOH (50µL concentrated HCl, per 500 mL MeOH) resulting in precipitation of white product.

Section S2: Synthesis and characterisation of Zn_2Na

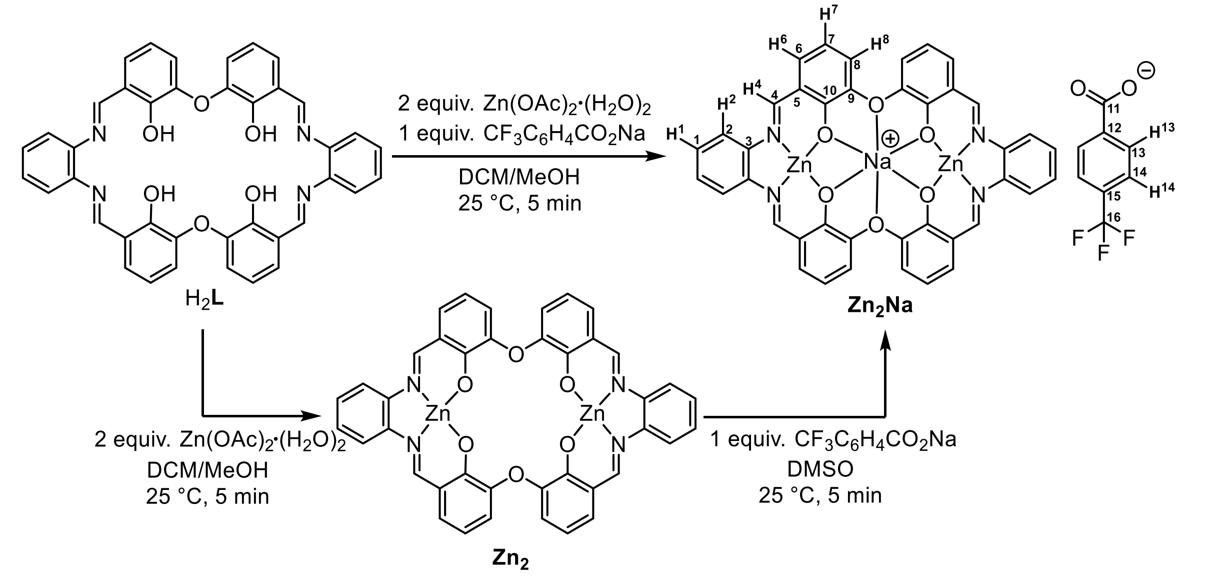

Scheme S1: Synthesis of $\mathbf{Zn_2Na}$ from $H_2\mathbf{L}$ and NMR assignment numbering for $\mathbf{Zn_2Na}$.

Synthesis of $\mathbf{Zn_2Na}$: A solution of $Zn(OAc)_2{\cdot}(H_2O)_2$ (33.3 mg, 151 µmol) and $CF_3C_6F_4CO_2Na$ (17.2 mg, 75 µmol) in MeOH (5 mL) was added to a solution of $H_2\mathbf{L}$ (50.0 mg, 75 µmol) in 5 mL DCM (5mL). The resulting solution was left unperturbed for 5 min. Afterwards all volatiles were removed, *in vacuo*, yielding a semi-solid which was washed with Et_2O (100 mL). To remove the acetic acid by-product fully, the crude product was suspended in toluene (20 mL) which was afterwards removed under vacuum. This process was repeated, yielding $\mathbf{Zn_2Na}{\cdot}2H_2O$ as an orange powder (74 mg, 73 µmol, 96%).

Alternatively, the addition of just $Zn(OAc)_2{\cdot}(H_2O)_2$ (33.3 mg, 151 µmol) to $H_2\mathbf{L}$ (50.0 mg, 75 µmol) resulted in the precipitation of an orange solid, which was washed with Et_2O (100 mL) and dried in vacuo yielding $\mathbf{Zn_2}$ (39 mg, 98 µmol, 65%). $\mathbf{Zn_2}$ is completely insoluble in all common organic solvents which prevents its characterisation. Yet the addition of $CF_3C_6F_4CO_2Na$ (11.2 mg, 98 µmol) in DMSO (5 mL) to a suspension of $\mathbf{Zn_2}$, quantitatively yielded $\mathbf{Zn_2Na}$ as established of ^{1}H NMR of an aliquot in d_6-DMSO**.**

^{1}H NMR (500 MHz, d_6-DMSO) δ 8.84 (s, 1H, H-4), 7.86 (s, 0.5H, H-13), 7.76 (dd, *J* = 6.0, 3.4 Hz, 1H, H-2), 7.57 (s, 0.5H, H-14), 7.42 (dd, *J* = 6.0, 3.3 Hz, 1H, H-1), 7.22 (d, *J* = 8.1 Hz, 1H, H-6), 7.16 (d, *J* = 8.1 Hz, 1H, H-8), 6.52 (t, *J* = 7.8 Hz, 1H, H-7).

^{13}C {^{1}H} NMR (126 MHz, d_6-DMSO) δ 168.01 (C-11), 164.05 (C-4), 162.52 (C-3), 148.58 (C-9), 140.40 (C-10), 130.40 (C-13), 129.74 (C-6), 127.65 (C-1, C-12), 124.38 (C-14), 124.31 (q, J = 271 Hz, C-16), 121.16 (C-5, C-15), 120.49 (C-8), 117.16 (C-2), 111.48(C-7).

^{19}F {^{1}H} NMR (470 MHz, d_6-DMSO) δ -60.98.

HRESI-MS m/z = calculated $[M - CF_3C_6F_4CO_2Na]^+$ 809.0141. Found 809.0135

Elemental Analysis ($\mathbf{Zn_2Na}{\cdot}2H_2O$, $C_{44}H_{32}F_3N_4NaO_{10}Zn_2$) calculated C 55.7% H 3.1% N 5.4% found C 55.7% H 3.1% N 5.2%

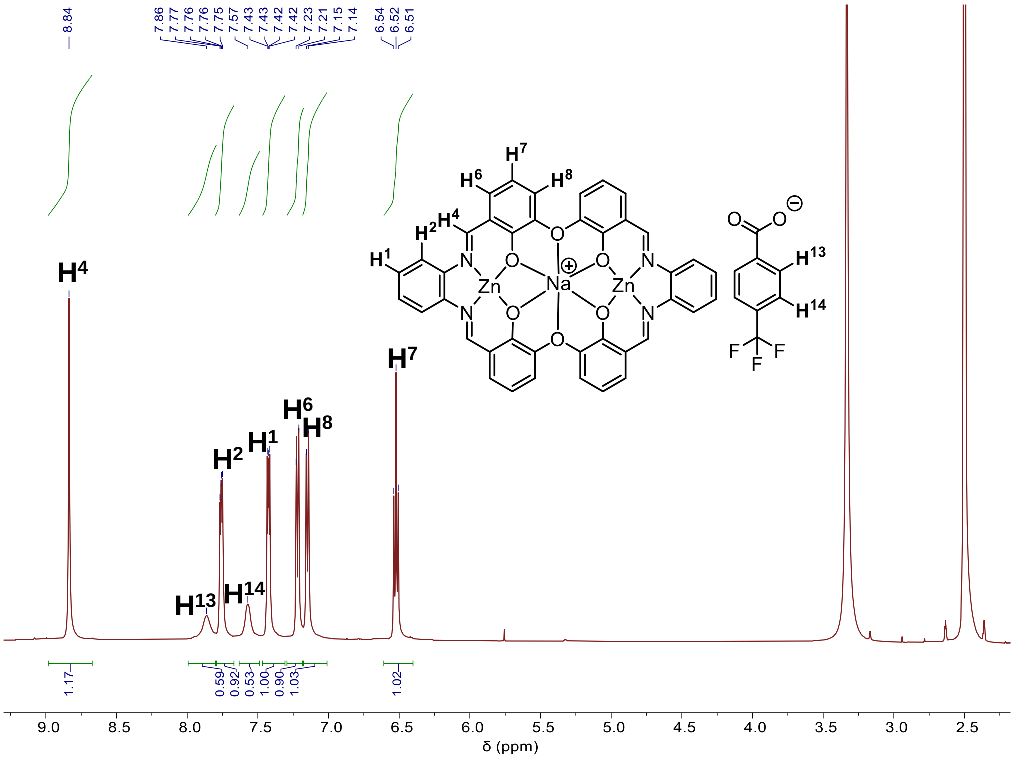

Figure S 1: ^{1}H NMR spectrum (500 MHz, d_6-DMSO, 25°C) of **Zn_2Na**.

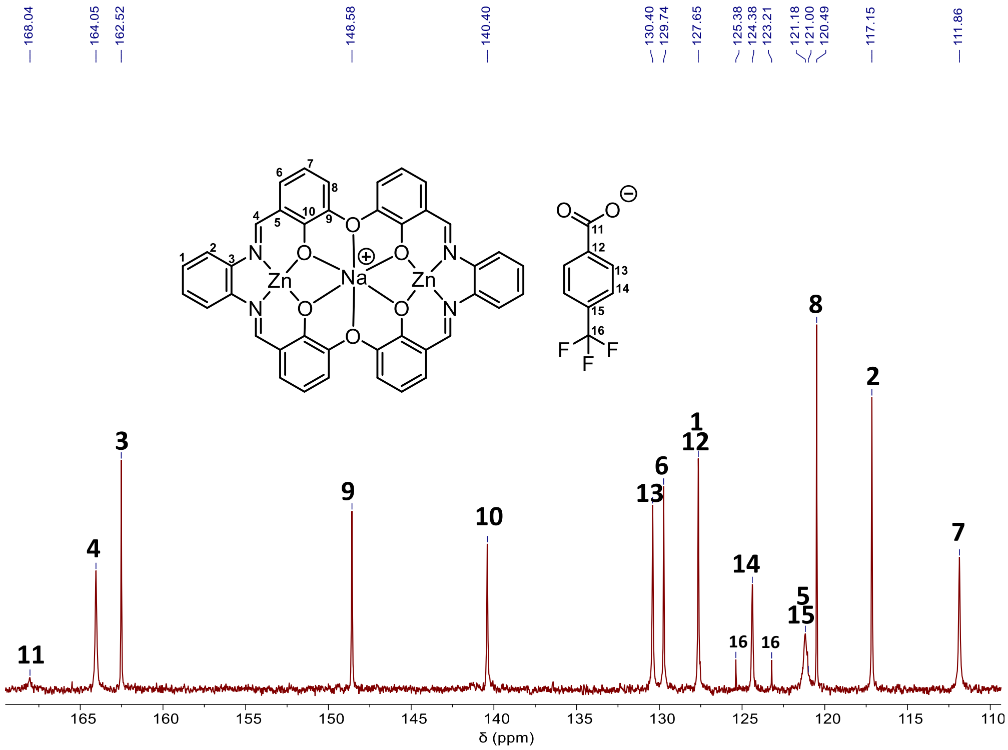

Figure S 2: ^{13}C {^{1}H} NMR (126 MHz, d_6-DMSO, 25°C) of **Zn_2Na**.

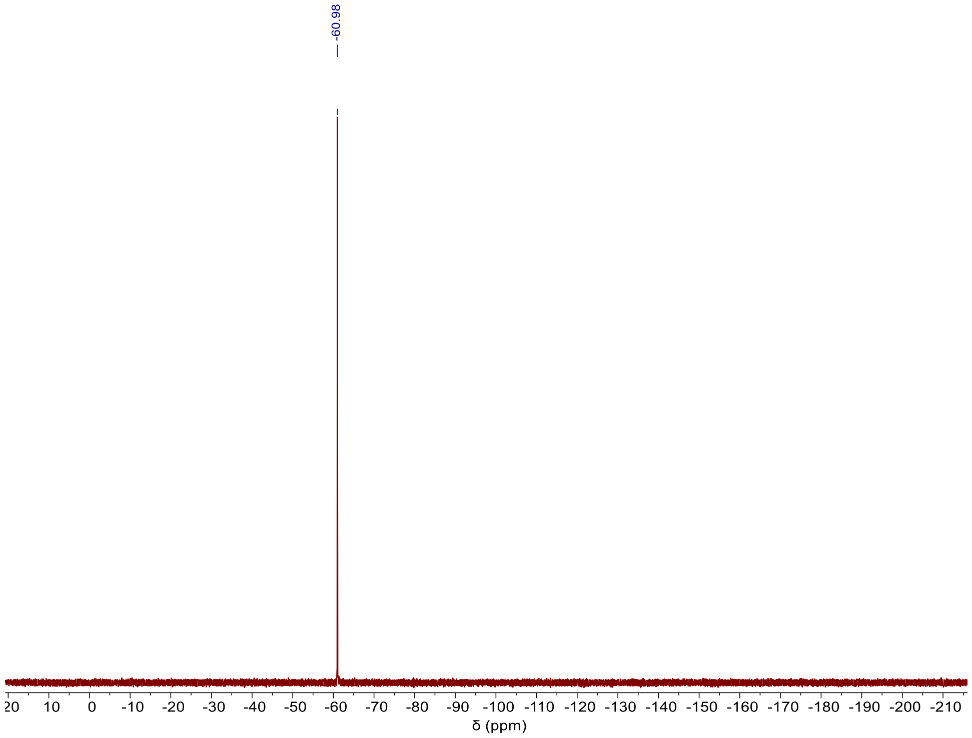

Figure S 3: ^{19}F {^{1}H} NMR (470 MHz, d_6-DMSO, 25°C) of **Zn_2Na**.

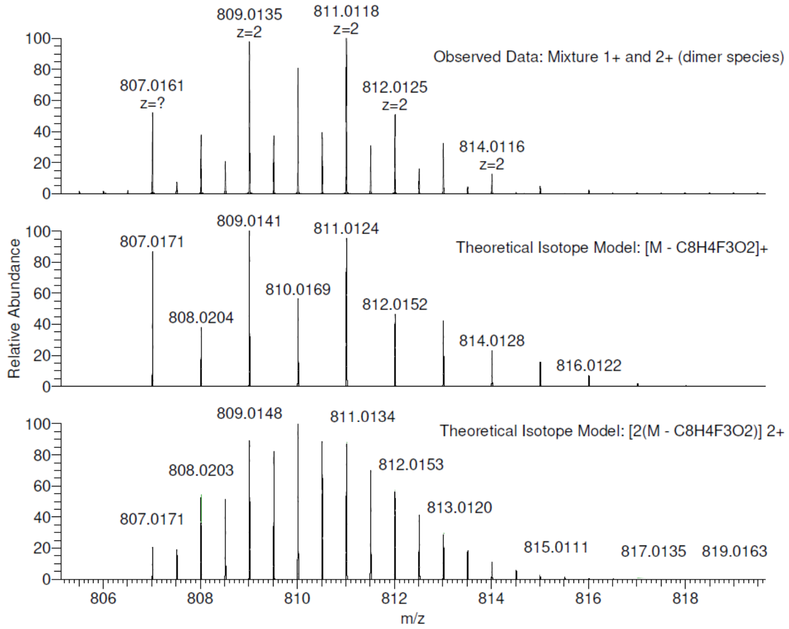

Figure S 4: Positive mode high-resolution ESI mass spectrum of **Zn_2Na.**

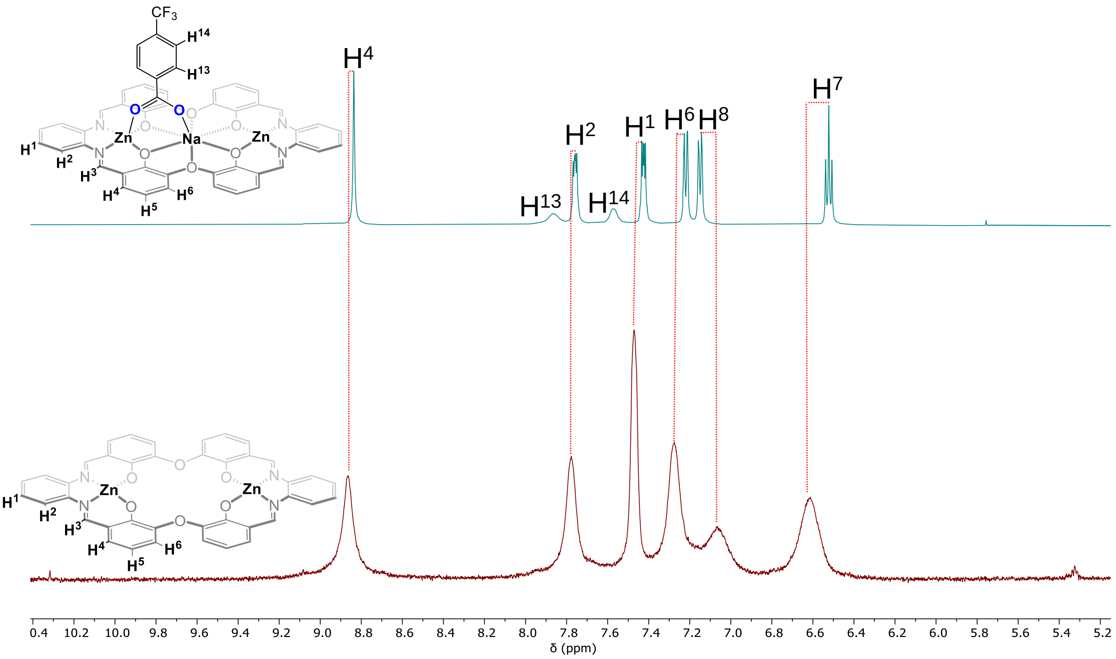

Figure S 5: ^{1}H NMR spectra (400 MHz, d_6-DMSO, 25°C) before (lower) and after (upper) sodium coordination. The lower spectrum corresponds to **Zn$_2$** (prepared *in situ* from $Zn(OAc)_2 \cdot (H_2O)_2$ and H_2**L** in d_6-DMSO) and was measured within 10 min of synthesis (by which time not all **Zn$_2$** had precipitated)**.** This experiment confirms Na coordination within the central all oxygen-binding pocket of **L**.

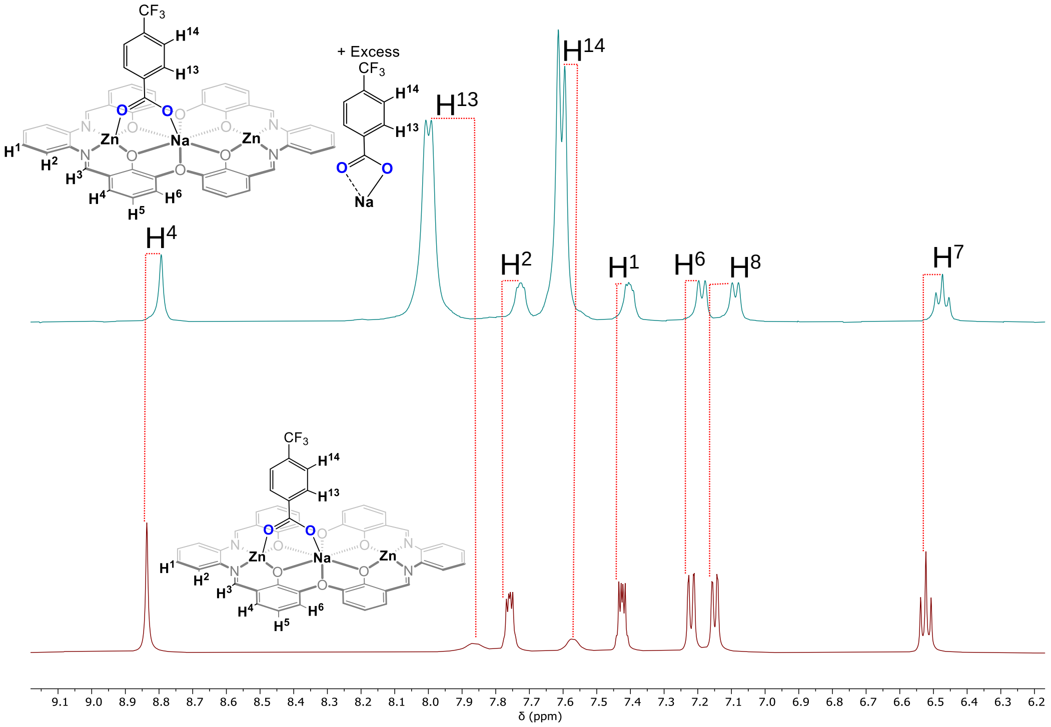

Figure S 6: ^{1}H NMR spectra (400 MHz, d_6-DMSO, 25°C) before (lower) and after (upper) the addition of excess (ca 10 equiv.) $CF_3C_6F_4CO_2Na$ to **Zn$_2$Na**.

Section S3: Kinetic data for CO_2/CHO ROCOP

Section S3.1: Kinetic data for CO_2/CHO ROCOP at different temperatures and pressures

Reaction Stirring: At epoxide conversions >30%, magnetic stirring becomes inefficient due to viscosity limitations, i.e. the polymerization stirring stops or significantly decreases in terms of speed. To minimize viscosity limitations, rare-earth magnetic stirrer bars (1 cm in length, 0.5 cm in maximal diameter) were applied in a narrow diameter Schlenk tube (12 cm in length, 1.75 cm in max diameter, 1.5-3 mL overall fill volume) at 1400 rpm stirring speed. It is important to note that any deviations from these conditions could change the conversion vs. time data and/or proportion of ether linkages.

Copolymer Determination and characterization procedures: The following characterisation techniques were used to establish that the different monomer sequences are present the same polymer chain, as opposed to the formation of two separate chains.

(a) Monomodal, narrow dispersity polymer molecular weight distributions were obtained for all copolymers. Also, aliquots taken during polymerizations showed increasing molar mass values with time, but not significant changes to dispersity or modality. The ^{1}H NMR spectra of these aliquots indicated compositions, at each time point, which correspond to the same composition indicated by *in situ* IR spectroscopy.

(b) All polymers showed ^{1}H DOSY NMR spectra in which all resonances showed a single diffusion coefficient.

(c) ^{1}H-^{1}H COSY NMR spectra allowed identification of different polymer sequence junction units with clear cross correlations.

(d) Polymer compositions, as determined by NMR spectroscopy, remained identical in crude polymerization samples and in products which were purified by fractionation techniques (i.e. precipitation from DCM solutions using MeOH).

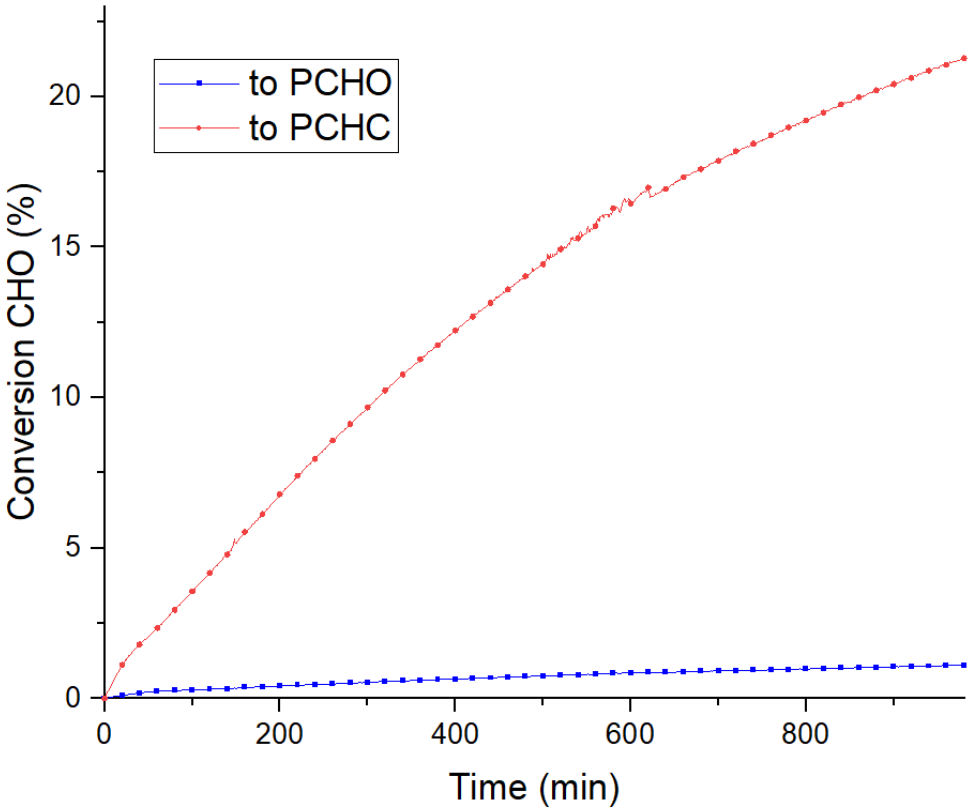

Figure S 7: Plot showing conversion of CHO to polymer (PCHC and PCHO) vs. time. Data are obtained using *in situ* ATR-IR spectroscopic monitoring of polymerizations conducted using 1 equiv. **Zn_2Na**, 20 equiv. cyclohexane diol (CHD), 4000 equiv. cyclohexene oxide (CHO) and 1 bar carbon dioxide pressure at 80 °C (Table S1, run #1). The conversion data was obtained by monitoring the changes to absorptions at 1744 cm^{-1} (PCHC) and 1089 cm^{-1} (PCHO). The initial rate and turn-over-frequency (TOF) were obtained from 0-12.5% polymer conversion.

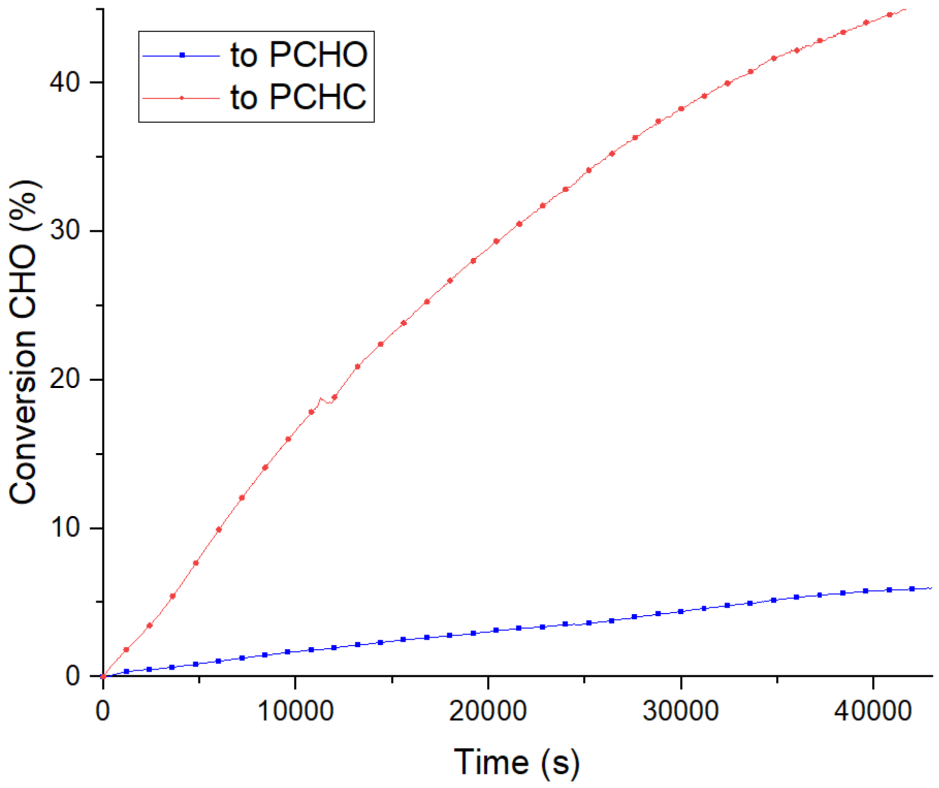

Figure S 8: Plot showing conversion of CHO to polymer (PCHC and PCHO) vs. time. Data are obtained using *in situ* ATR-IR spectroscopic monitoring of polymerizations conducted using 1 equiv. **Zn_2Na**, 20 equiv. cyclohexane diol (CHD), 4000 equiv. cyclohexene oxide (CHO) and 1 bar carbon dioxide pressure at 90 °C (Table S1, run #2). The conversion data was obtained by monitoring the changes to absorptions at 1744 cm^{-1} (PCHC) and 1089 cm^{-1} (PCHO). The initial rate and turn-over-frequency (TOF) were obtained from 0-15% polymer conversion.

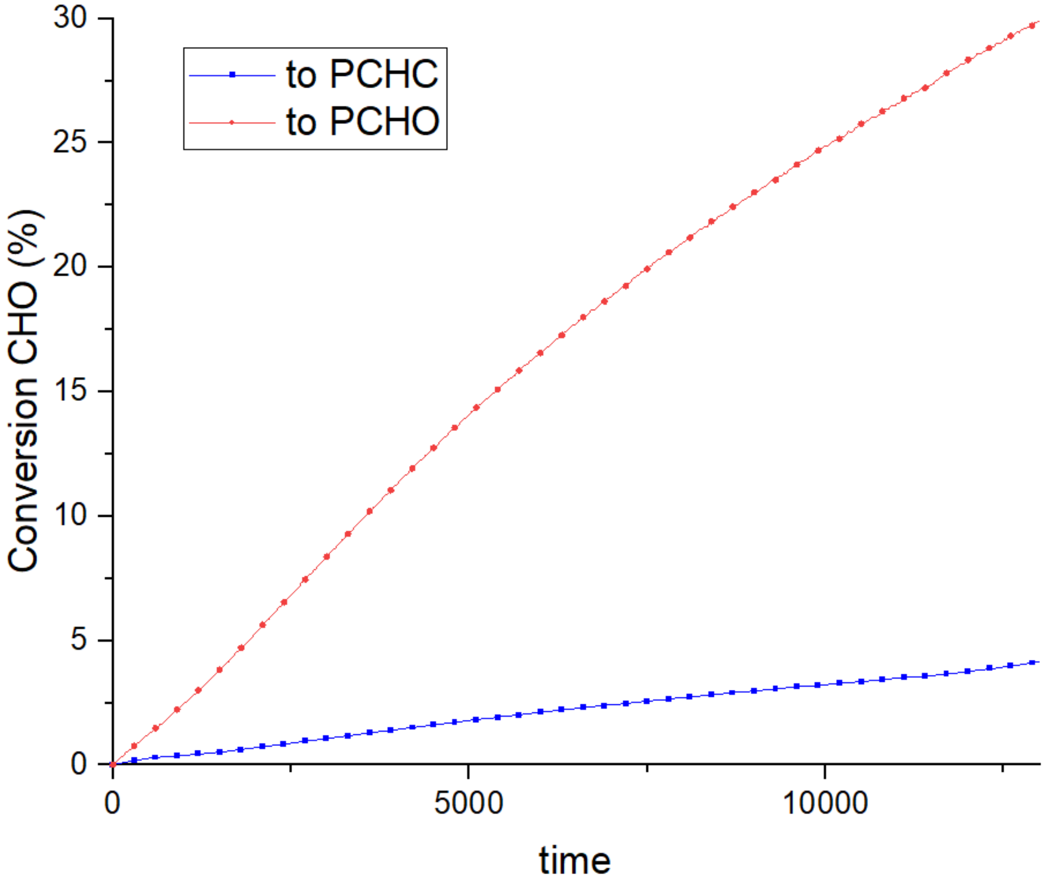

Figure S 9: Plot showing conversion of CHO to polymer (PCHC and PCHO) vs. time. Data are obtained using *in situ* ATR-IR spectroscopic monitoring of polymerizations conducted using 1 equiv. **Zn_2Na**, 20 equiv. cyclohexane diol (CHD), 4000 equiv. cyclohexene oxide (CHO) and 1 bar carbon dioxide pressure at 100 °C (Table S1, run #3). The conversion data was obtained by monitoring the changes to absorptions at 1744 cm^{-1} (PCHC) and 1089 cm^{-1} (PCHO). The initial rate and turn-over-frequency (TOF) were obtained from 0-15% polymer conversion.

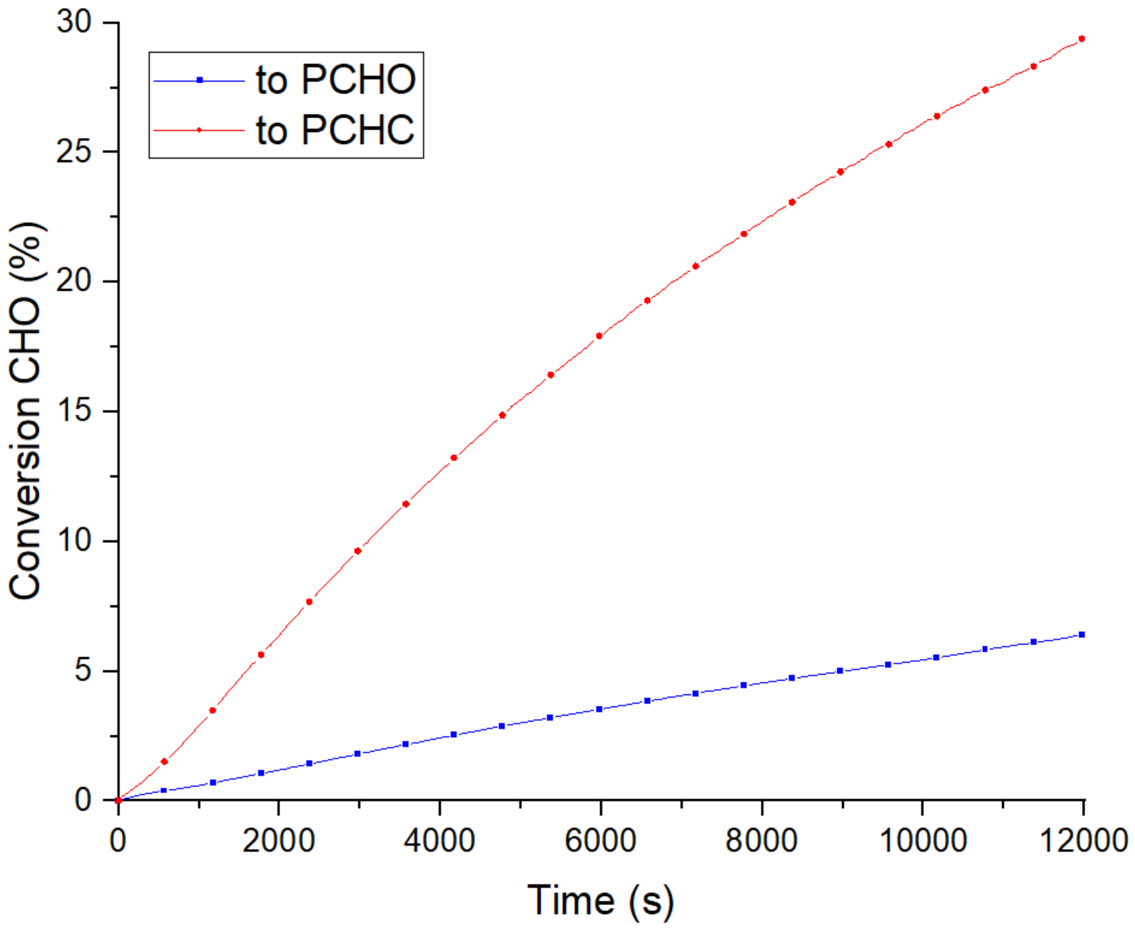

Figure S 10: Plot showing conversion of CHO to polymer (PCHC and PCHO) vs. time. Data are obtained using *in situ* ATR-IR spectroscopic monitoring of polymerizations conducted using 1 equiv. **Zn_2Na**, 20 equiv. cyclohexane diol (CHD), 4000 equiv. cyclohexene oxide (CHO) and 1 bar carbon dioxide pressure at 110 °C (Table S1, run #4). The conversion data was obtained by monitoring the changes to absorptions at 1744 cm^{-1} (PCHC) and 1089 cm^{-1} (PCHO). The initial rate and turn-over-frequency (TOF) were obtained from 0-15% polymer conversion.

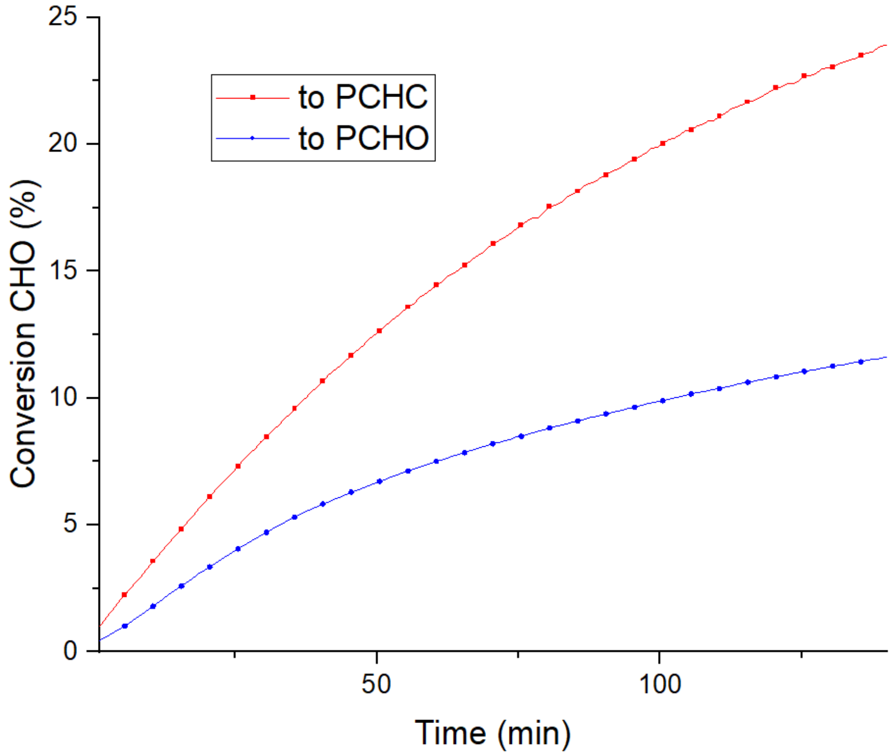

Figure S 11: Plot showing conversion of CHO to polymer (PCHC and PCHO) vs. time. Data are obtained using *in situ* ATR-IR spectroscopic monitoring of polymerizations conducted using 1 equiv. **Zn_2Na**, 20 equiv. cyclohexane diol (CHD), 4000 equiv. cyclohexene oxide (CHO) and 1 bar carbon dioxide pressure at 120 °C (Table S1, run #5). The conversion data was obtained by monitoring the changes to absorptions at 1744 cm^{-1} (PCHC) and 1089 cm^{-1} (PCHO). The initial rate and turn-over-frequency (TOF) were obtained from 0-15% polymer conversion.

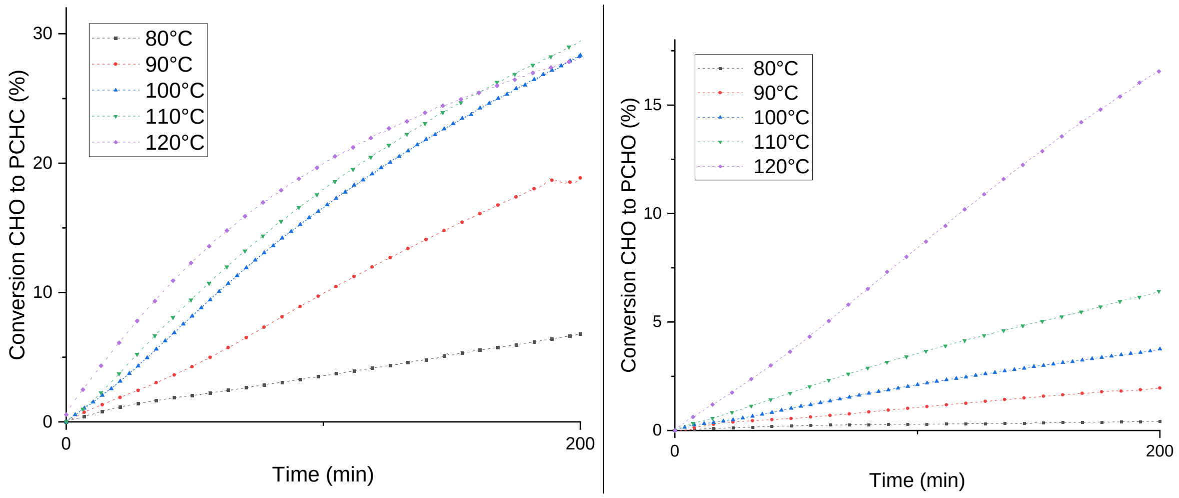

Figure S 12: Plots comparing the conversion of CHO to PCHC and PCHO from **Figure S7 – S11**.

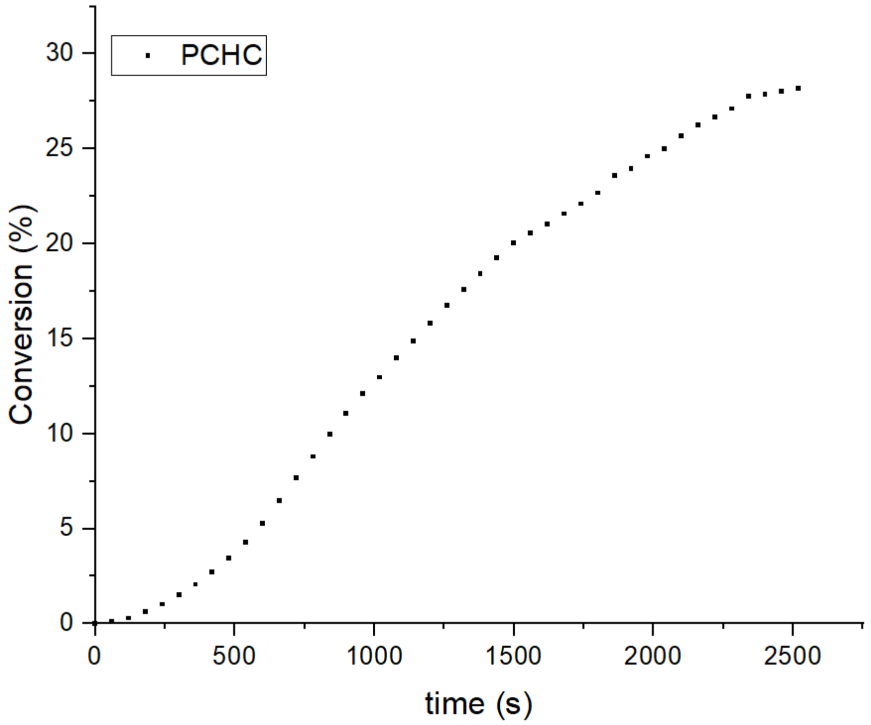

Figure S 13: Plot showing conversion of CHO to polymer (PCHC) vs. time. Data are obtained using *in situ* ATR-IR spectroscopic monitoring of polymerizations conducted using 1 equiv. **Zn_2Na**, 20 equiv. cyclohexane diol (CHD), 4000 equiv. cyclohexene oxide (CHO) and 20 bar carbon dioxide pressure at 120 °C (Table S1, run #6). The conversion data was obtained by monitoring the changes to absorptions at 1744 cm^{-1} (PCHC). The initial rate and turn-over-frequency (TOF) were obtained from 5-20% polymer conversion. Note that the non-linearity from 0-5% conversion arises due to the high-pressure reactor not reaching the target polymerization temperature until 5% conversion

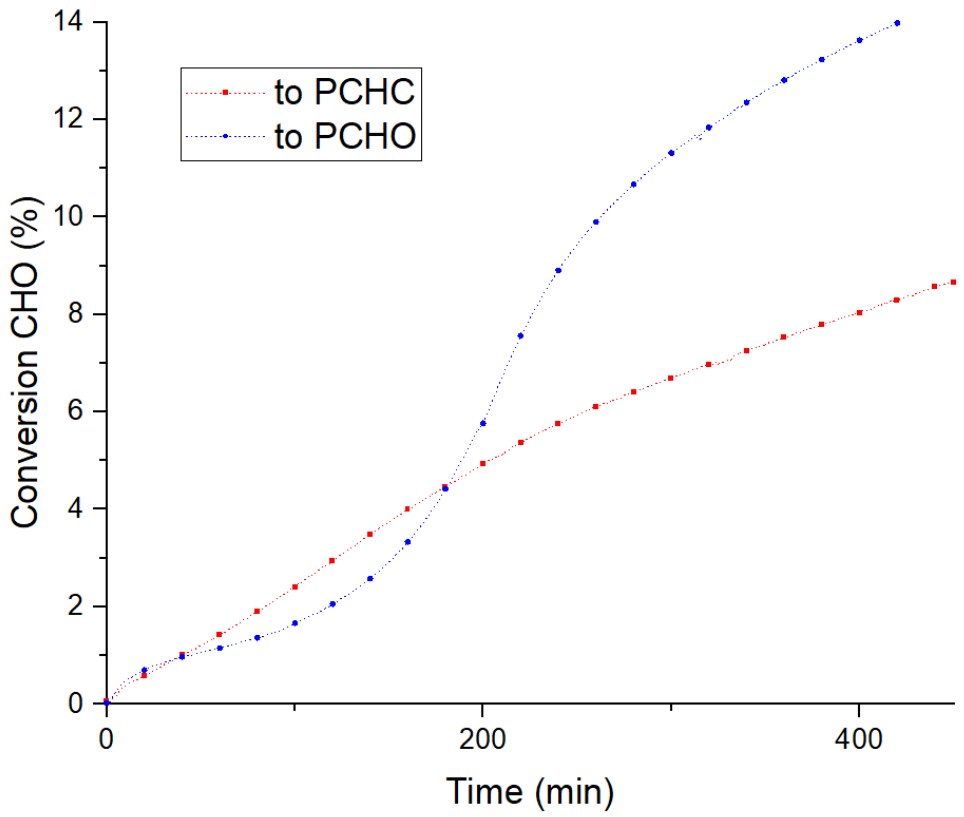

Figure S 14: Plot showing conversion to polymer (PCHC and PCHO) vs. time for a polymerization conducted using **Zn_2Na** (1 equiv), CHD (20 equiv.), CHO (4000 equiv.) under an atmosphere comprising carbon dioxide (0.5 bar) and nitrogen (0.5 bar) at 80 C. The initial rate and TOF were obtained from 0-12.5% conversion overall (i.e. PCHC+PCHO conversion).

T / ºC	Polymer selectivity[b] / %	Selectivity for PCHC:PCHO[c] / %	TOF Polymer / h^{-1}, (TON)[d]	CHO Conversion	Polymer M_n ($M_{n,theo}$) / kg/mol[f], Đ[e]
80[a]	99	>95:<5	75 , (920)	23%	2.42 (6.53), 1.20
90[a]	98	90:10	270, (2400)	60%	6.86 (17.04),1.24
100[a]	97	86:14	478, (1960)	49%	5.61 (13.92), 1.29
110[a]	96	82:18	578, (2120)	53%	7.12 (15.05), 1.26
120[a]	94	67:33	956, (2000)	50%	5.12 (14.20), 1.24
80[g]	99	41:59	200, (1200)	30%	2.38 (8.52), 1.22
120[h]	96	>95:<5	2900, (1360)	34%	5.35 (9.66), 1.16

Table S1: Table showing the data for polymerizations illustrated in this section. [a]Copolymerization conditions: 0.025 mol% catalyst loading (1:4000, Zn_2Na:CHO), 20 equiv. 1,2-cyclohexane diol (CHD), 1 bar CO_2, CHO neat (9.99 M). Polymerizations were stopped after 20 h or once conversion plateaued (see end of plot of respective graphs).[b]Determined by comparison of the relative integrals, in the normalised ^{1}H NMR spectrum, of resonances due to polymer (δ 4.65 ppm, 3.45 ppm) and cyclic carbonate (δ 4.00 ppm).[c]Determined by comparison of the relative integrals, in the normalised the ^{1}H NMR spectrum, of resonances due to (poly)carbonate (δ 4.65 ppm) and (poly)ether (δ 3.45 ppm) linkages. [d]Turnover number (TON), number of moles of CHO consumed per mole of catalyst.[e]Turnover frequency (TOF) determined from initial rates using in situ ATR-IR spectroscopy (typically from 5 – 15% conversion) as TON/time. [f]Determined by GPC (gel permeation chromatography) measurements conducted in THF, using narrow MW polystyrene standards to calibrate the instrument; $Đ = M_w/M_n$. [g] Polymerization conducted using unpurified gas mixture comprising 0.5 bar CO_2 and 0.5 bar N_2 with otherwise analogous conditions. [h]Polymerization conducted using 20 bar CO_2 pressure, in a stainless steel reactor, with mechanical stirring.

Section S3.2: Comparison of literature know catalysts to Zn_2Na

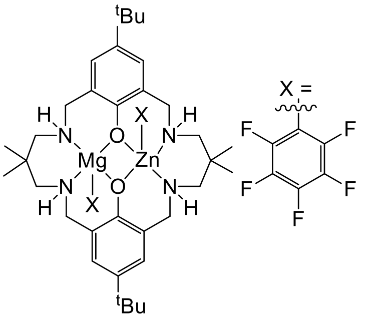

TOF 50 h^{-1} at 80 °C, 0.1 mol%, 1 bar CO_2
>99% PCHC
J. Am. Chem. Soc, 2020, 142, 9, 4367 - 4378

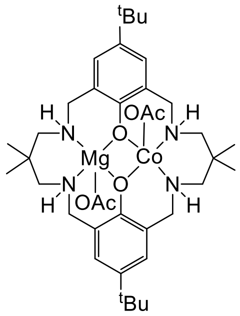

TOF 350 h^{-1} at 100 °C, 0.05 mol%, 1 bar CO_2
>99% PCHC
Nat. Chem., 2020, 12, 372 - 380

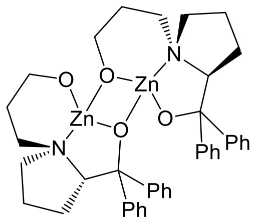

TOF 74 h^{-1} at 80 °C, 0.07 mol%, 1 bar CO_2
>99% PCHC
Chem. Eur. J., 2017, 23, 16472 - 16475

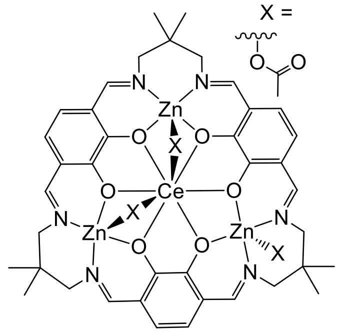

TOF 100 h^{-1} at 100 °C, 0.05 mol%, 3 bar CO_2
>99% PCHC
Angew. Chem. Int. Ed., 2018, 57, 2492 - 2496

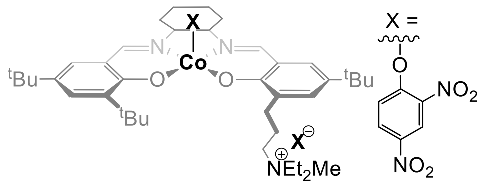

TOF 136 h^{-1} at 50 °C, 0.02 mol%, 1 bar CO_2
>99% PCHC
Macromolecules, 2010, 43, 1396 - 1402

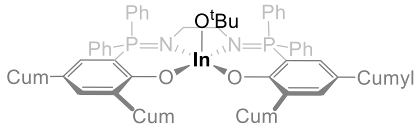

TOF 15 h^{-1} at 80 °C, 1 mol%, 1 bar CO_2
>99% PCHC
J. Am. Chem. Soc., 2018, 140, 6893 - 1903

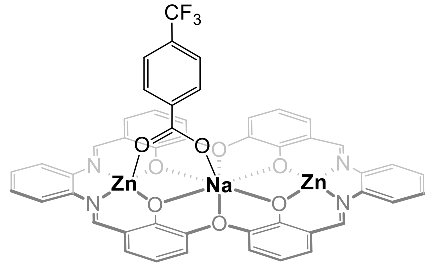

This contribution:
TOF 478 h^{-1} at 100 °C, 0.025 mol%, 1 bar CO_2
86% PCHC, 14% PCHO

Figure S 15: Selected high activity, under low carbon dioxide pressure (<3 atm), catalysts for CO_2/CHO ROCOP. Note that the activity values are quoted per initiating group in each case.

Section S3.3: *In situ* Temperature switching and polymerisation without CHD

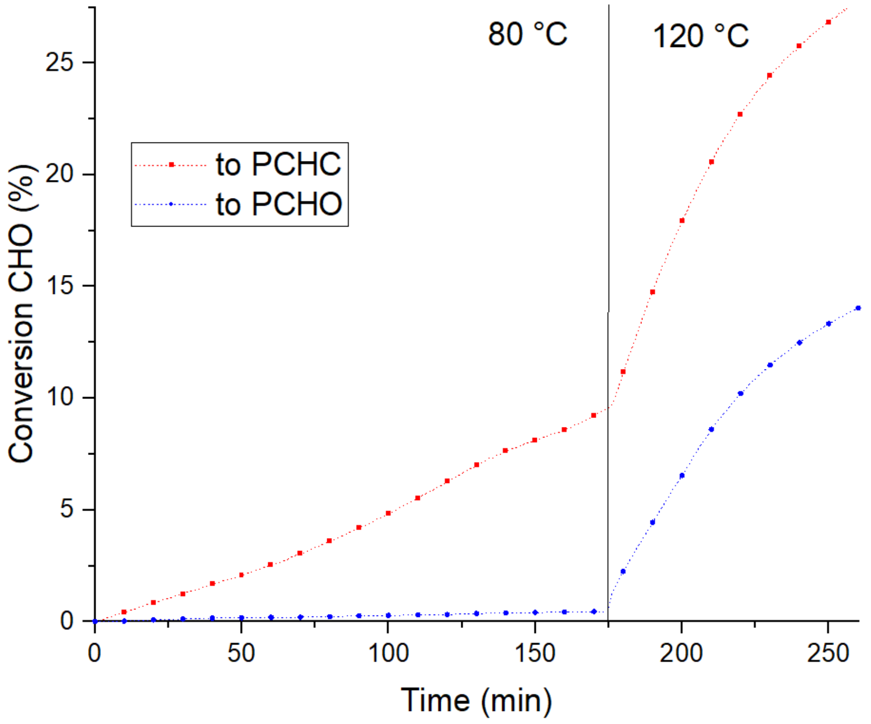

Figure S 16: Conversion vs. time plot for the formation of PCHO and PCHC, with a temperature 'switch' occurring from 80 - 120 °C, at 175 min. Polymerization conditions: 1 equiv. **Zn_2Na:** 20 equiv. CHD: 4000 equiv. CHO.

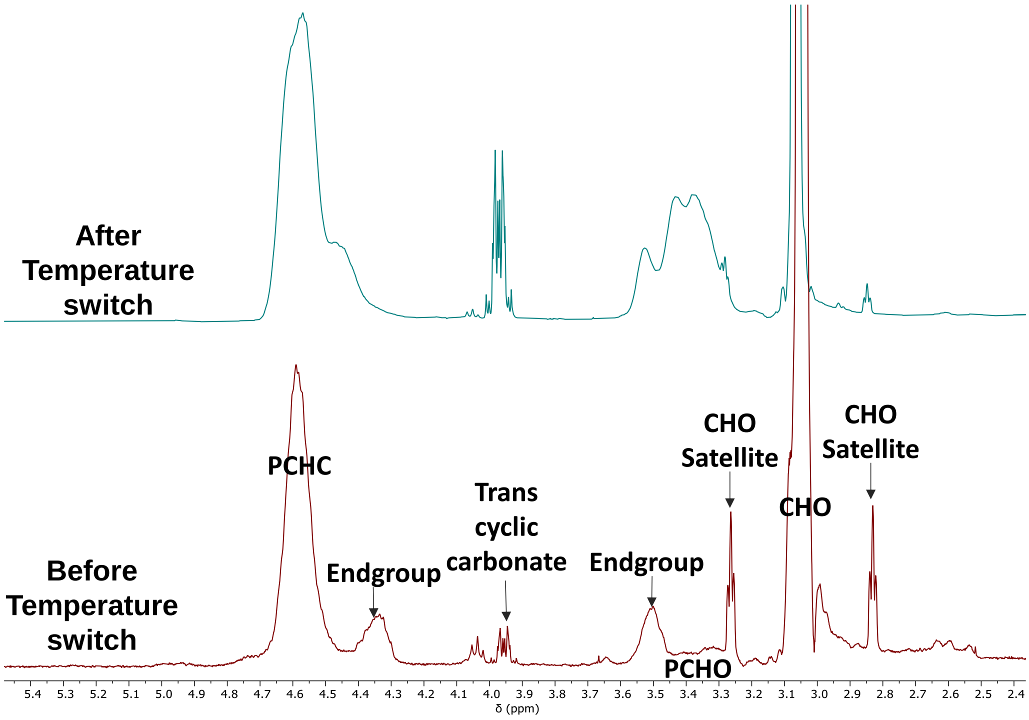

Figure S 17: Comparison of the *in situ* ^{1}H NMR spectra (400 MHz, $CDCl_3$, 25°C) of polymer samples taken before (lower) the temperature change (i.e. at 80 °C), and after 1.5 h of further reaction (i.e. at

120 °C, upper). Note the increase in the resonance assigned to PCHO (~ 3.35 ppm) arises from the increase in temperature.

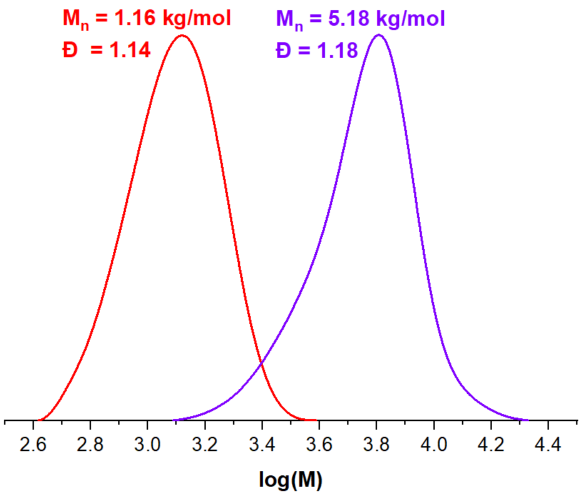

Figure S 18: GPC traces of aliquots removed during the polymerization described above (Fig. S15, 16). The red trace is from the aliquot taken from the reaction at 80 °C and the purple trace taken after the reaction temperature was increased to 120 °C.

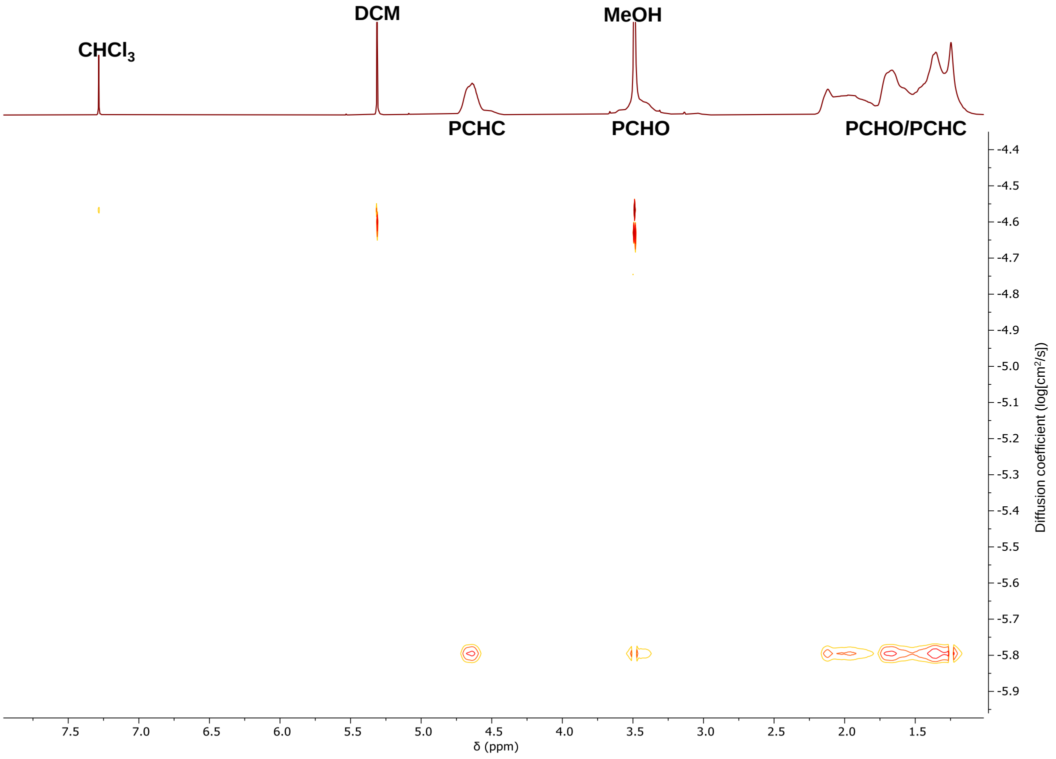

Figure S 19: Representative ^{1}H DOSY NMR spectrum (400 MHz, $CDCl_3$, 25°C) of the polymer produced according to any conditions shown in Table S1 . The plot shows that the carbonate and ether resonances diffuse at the same rate and are, therefore, attached to one another.

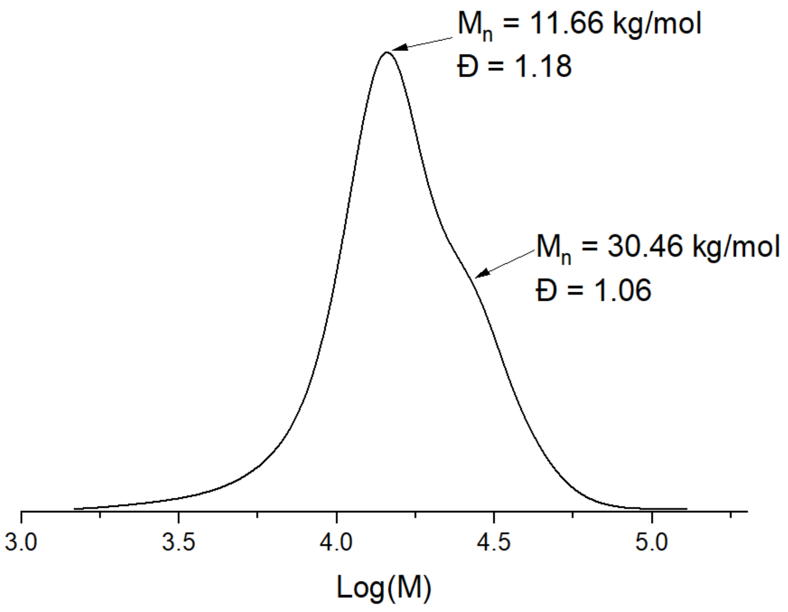

Figure S 20: GPC trace of CO_2/CHO ROCOP conducted without added chain transfer agent. The molecular weight distribution is bimodal and this is attributed to chains being initiated both by the benzoate coligand and by 1,2-cyclohexanediol present or generated by reaction of water with CHO. Catalysis conditions: 1 equiv. **Zn_2Na**, 4000 equiv. cyclohexene oxide (CHO) and 1 bar carbon dioxide pressure at 100 °C, 16 h.

Section S4: CHO ROP

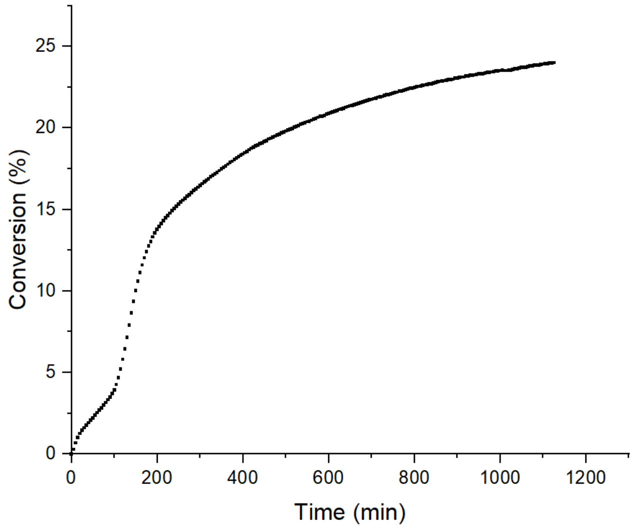

Figure S 21*:* Plot showing conversion of CHO to polymer (PCHO) vs. time. Data are obtained using *in situ* ATR-IR spectroscopic monitoring of polymerizations conducted using 1 equiv. **Zn_2Na**, 20 equiv. cyclohexane diol (CHD), 4000 equiv. cyclohexene oxide (CHO), 1 bar nitrogen pressure, at 120 °C. The conversion data was obtained by monitoring the changes to absorptions at 1089 cm^{-1} (PCHO). The initial rate and turn-over-frequency (TOF) were obtained from 3-14% polymer conversion

Note that during CHO ROP, using **Zn_2Na*,*** complete CHO conversion was not achieved. The maximum CHO conversion was also observed to decrease with increasing temperature. This phenomenon does not arise due to catalyst decomposition as the reaction can be switched to CO_2/CHO ROCOP (after CHO ROP conversion has apparently stopped). These data appear to indicate that CHO ROP may be equilibrium limited but such an explanation is not consistent with the fact that epoxide ROP is heavily exergonic. We infer that **Zn_2Na** catalyses the depolymerisation of PCHO, through a different mechanism to CHO ROP, and hence seemingly pseudo-equilibrates the ROP of CHO. This effect will be the subject to further studies and lies outside the scope of this contribution.

T [°C]	k_{obs} [s^{-1}], (R^2)
80	($2.54*10^{-5}$± $9.22*10^{-8}$), (0.99)
90	($5.41*10^{-5}$± $4.15*10^{-7}$), (0.99)
100	($6.18*10^{-5}$ ± $5.71*10^{-7}$), (0.99)
110	($8.13*10^{-5}$ ± $4.59*10^{-7}$), (0.99)
120	($1.05*10^{-4}$ ± $1.74*10^{-6}$), (0.99)

Table S2: k_{obs} values for CHO ROP under conditions outlined in this section (k_{obs} = gradient of linear fitting of the logarithm of CHO concentration vs time).

Section S5: Synthesis and characterisation of Zn_2Na_{BArF}

In order to confirm that CHO ROP catalysed by **Zn_2Na** features an anionic rather than a cationic propagating chain end,a derivative without anionic initiator was prepared: **$Zn_2NaBArF$** and its performane in CHO ROP compared. As presented in this section, polymerization results in significantly broader disperity values and (near instantaneous) much faster CHO ROP for **$Zn_2NaBArF$** than for **Zn_2Na.** These findings are proposed to be a consequence of cationic rather than anionic propagation mechanisms. The findings suggest that CHO ROP using **Zn_2Na** occurs by anionic (coordination insertion) propagation mechanisms.

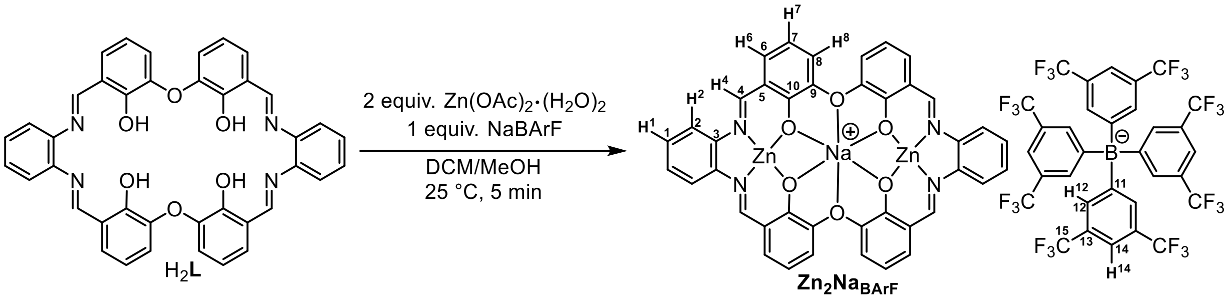

Scheme S2: Synthesis of **$Zn_2NaBArF$** from H_2**L** and NMR assignment numbering for **$Zn_2NaBArF$**.

Synthesis of Zn_2Na_{BArF}: A solution of $Zn(OAc)_2(H_2O)_2$ (33.3 mg, 151 µmol) and tetrakis(3,5-bis(trifluoromethyl)phenyl)borate NaBArF (67.1 mg, 75 µmol) in MeOH (5 mL) was added to a solution of H_2**L** (50.0 mg, 75 µmol) in DCM (5 mL). The resulting solution was left unperturbed for 5 min. Afterwards all volatiles were removed in vacuum yielding a semi-solid. In order to remove residual AcOH by-product, the crude material was suspended in toluene (20 mL), which was afterwards removed undedr vacuum. This process was repeated yielding **Zn_2Na_{BArF}**$\cdot 4H_2O$ as an orange powder (123 mg, 73 µmol, 98%).

^{1}H NMR (500 MHz, d_6-DMSO) δ 8.91 (s, 1H, H-4), 7.82 (dd, *J* = 6.0, 3.4 Hz, 1H, H-2), 7.72 (s, 1H, H-14), 7.62 (s, 2H, H-12), 7.47 (dd, *J* = 6.0, 3.3 Hz, 1H,H-1), 7.27 (dd, *J* = 8.0, 1.7 Hz, 1H, H-6), 7.20 (dd, *J* = 7.7, 1.7 Hz, 1H, H-8), 6.58 (t, *J* = 7.8 Hz, 1H,H-7).

^{13}C {^{1}H} NMR (126 MHz, d_6-DMSO) δ 165.12, 162.63, 161.40 (q, *J* = 99.6), 149.09, 140.34, 134.52, 131.10, 128.97 (q, *J* = 31.8, 30.6 Hz), 128.44, 124.48 (q, *J* = 272.4 Hz), 121.96, 120.86, 118.26, 117.83, 112.98

^{19}F{^{1}H} NMR (376.5 MHz, d_6-DMSO) δ –61.6

HRESI-MS m/z = (M - $[\{3,5\text{-}(CF_3)_2C_6H_3\}_4B])^+$ calculated 809.0141 found 809.0144

Elemental Analysis (**Zn_2Na_{BArF}** $\cdot 4H_2O$, $C_{72}H_{44}BF_{24}N_4NaO_{10}$) calculated C 49.5% H 2.5% N 3.2% found C 49.8% H 2.2% N 3.1%

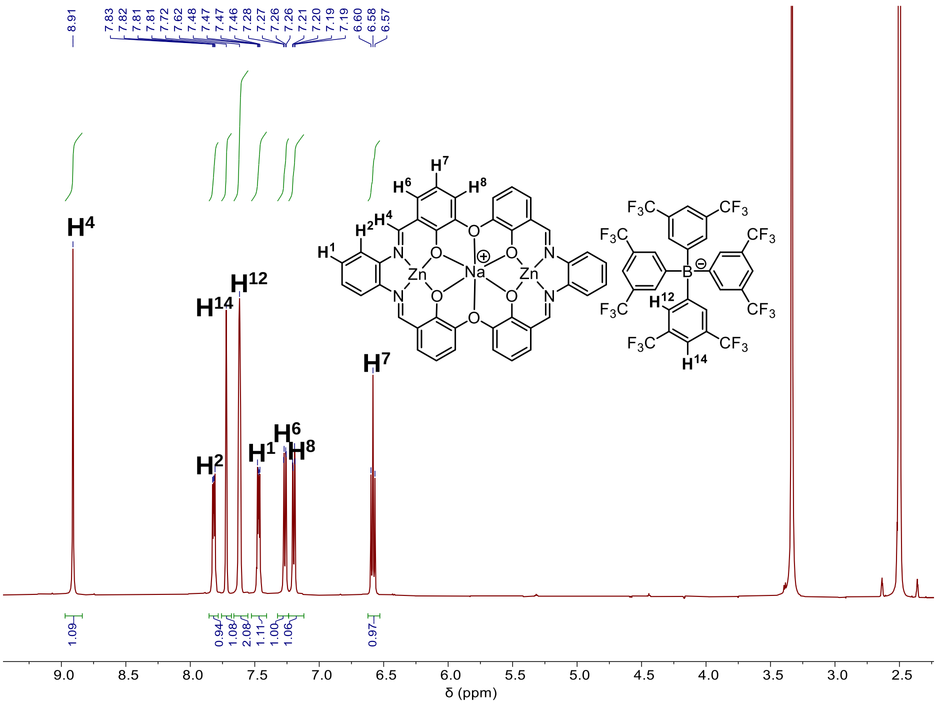

Figure S 22: ^{1}H NMR spectrum (500 MHz, d_6-DMSO, 25°C) of **Zn_2Na_{BArF}**.

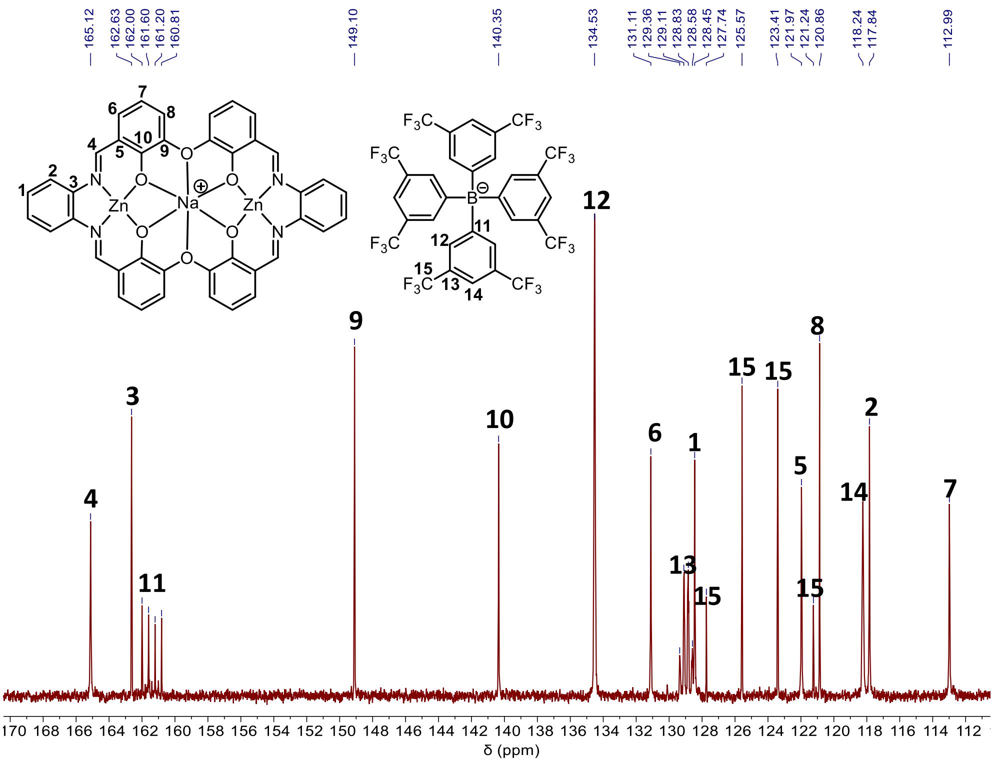

Figure S 23: ^{13}C {^{1}H} NMR spectrum (126 MHz, d_6-DMSO, 25°C) of **Zn_2Na_{BArF}**.

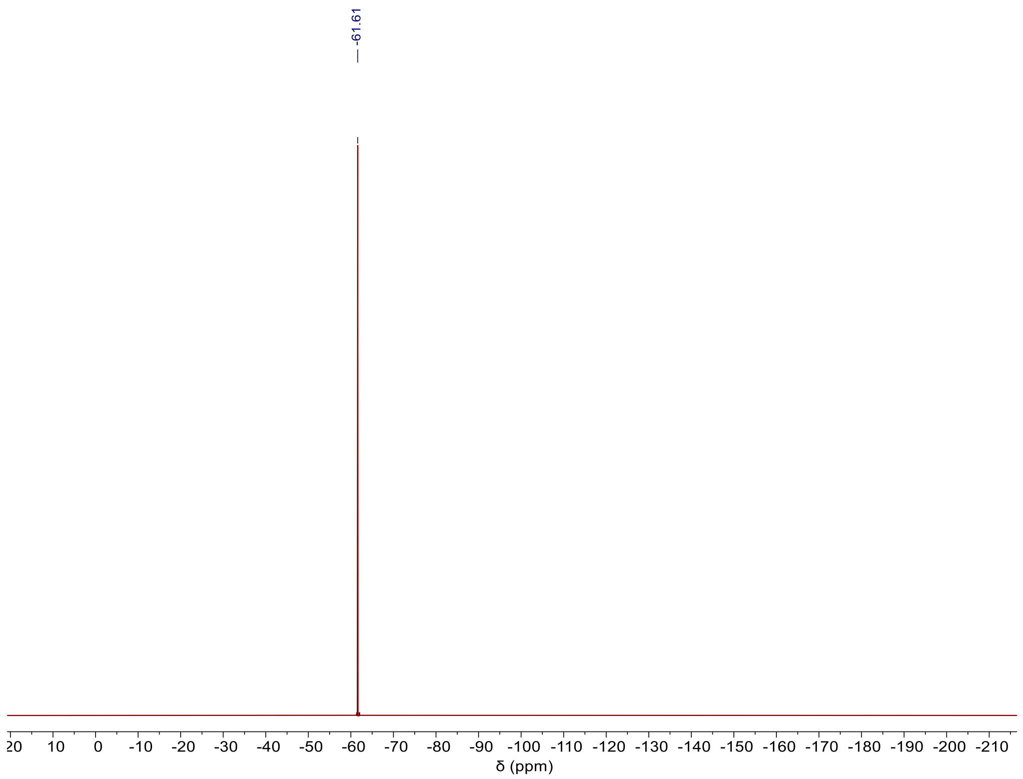

Figure S 24: ^{19}F {^{1}H} NMR spectrum (470 MHz, d_6-DMSO, 25°C) of **Zn_2NaBArF**.

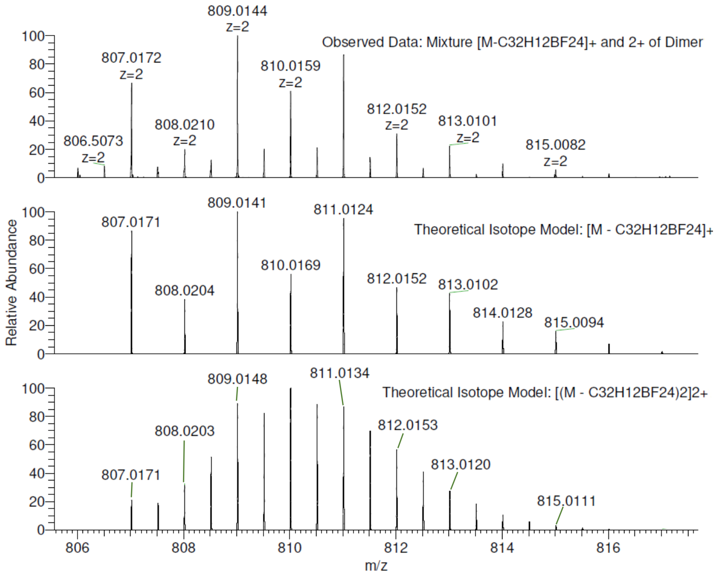

Figure S 25: Positive mode high-resolution ESI mass spectrum of **Zn_2NaBArF**

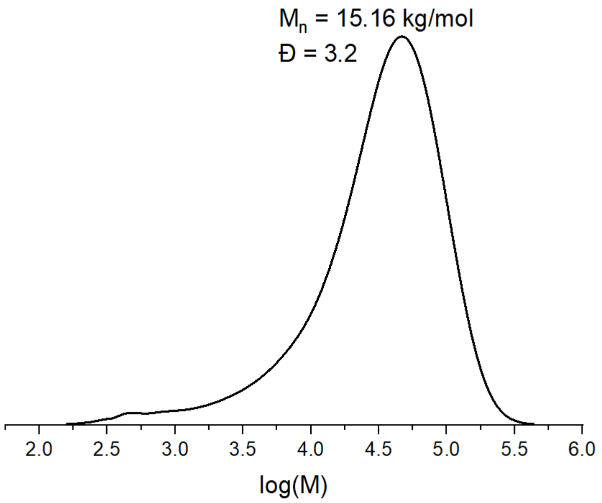

Figure S 26: Representative GPC trace of PCHO synthesized by **Zn_2NaBArF.** Polymerization conditions: **Zn_2NaBArF** (1 equiv), CHD (20 equiv.), CHO (4000 equiv.), N_2 (1 bar), 80 °C.

Section S6: PA/CHO ROCOP

T [°C]	k_{obs} [s^{-1}], (R^2)
100	$1.23*10^{-4} \pm 9.30*10^{-7}$, (0.99)
110	$2.59*10^{-4} \pm 7.97*10^{-7}$, (0.99)
120	$4.68*10^{-4} \pm 4.94*10^{-6}$, (0.99)
130	$7.70*10^{-4} \pm 4.20*10^{-6}$, (0.99)

Table S3: k_{obs} values for PA/CHO ROCOP (k_{obs} = gradient of linear fitting of the PA concentration vs time). Polymerization conditions: **Zn_2Na** (1 equiv), CHD (20 equiv.), PA (100 equiv.), CHO (4000 equiv.), N_2 (1 bar), 100 - 130 °C. In all cases perfectly alternating PCHPE was obtained, as confirmed by ^{1}H NMR spectroscopy.

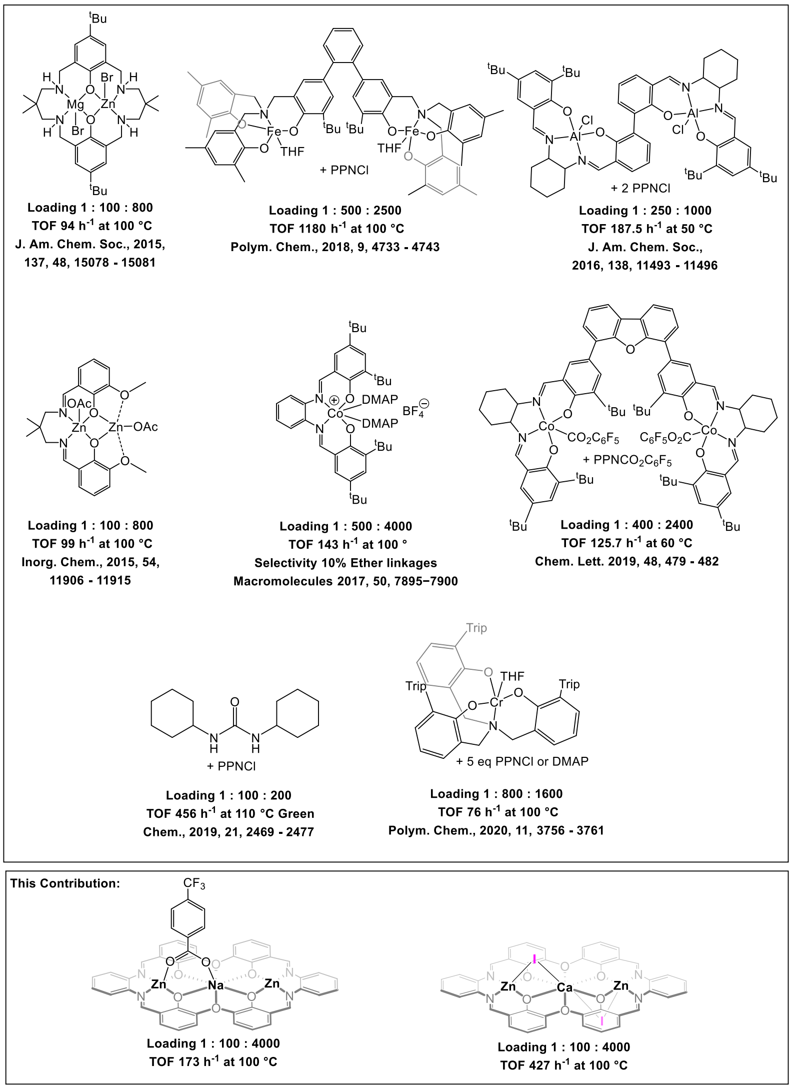

Figure S 27: High activity, PA/CHO ROCOP catalysts previously reported in the literature. Activity is given per initiator and the loading is reported for Catalyst : PA : CHO.

Section S7: Switchable Catalysis

Section S7.1: CO_2/CHO ROCOP to CHO ROP

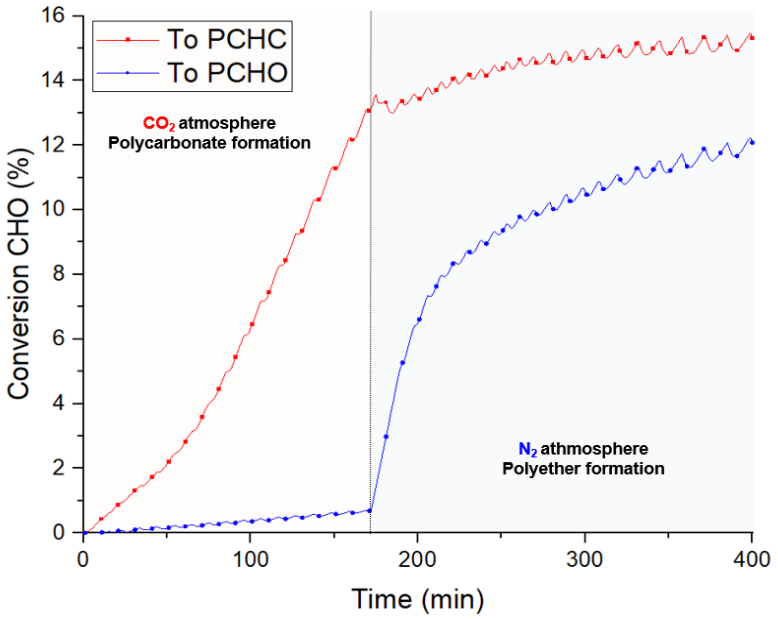

Figure S 28: Plot showing polymer conversion vs. time for a polymerization conducted with a change in gas atmosphere from carbon dioxide to nitrogen occurring after 160 mins. Polymerization conditions as per Table S1 #1. Note that heating increasing the temperature to 120°C after PCHO block formation (in order to investigate potential PCHC depolymerisation) left polymer composition unchanged.

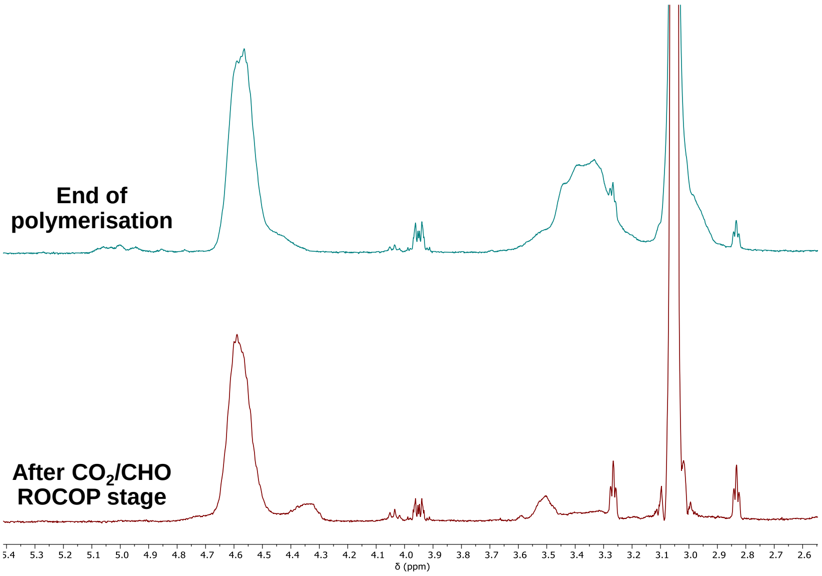

Figure S 29: Comparison of the ^{1}H NMR spectra (400 MHz, $CDCl_3$, 25°C) for aliquots removed at different stages of the switchable polymerization presented above (Fig. S28). (Lower) Under a carbon dioxide atmosphere and at the end of the reaction, under a nitrogen atmosphere (Upper).

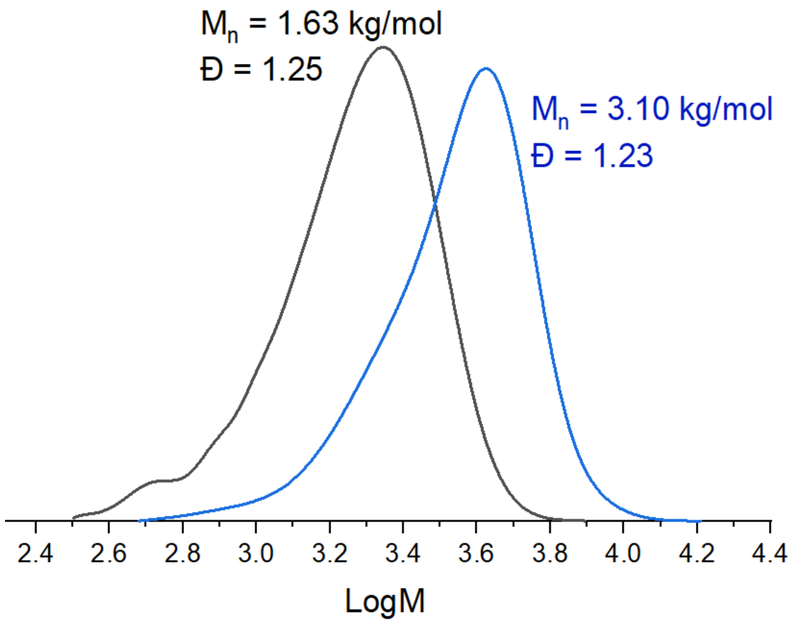

Figure S 30: GPC traces taken from the polymerization conducted under a carbon dioxide (black) and, later, under a nitrogen (blue) atmosphere, respectively. Polymerization conditions are as per Table S1.

Section S7.2: CHO ROP to CO_2/CHO ROCOP

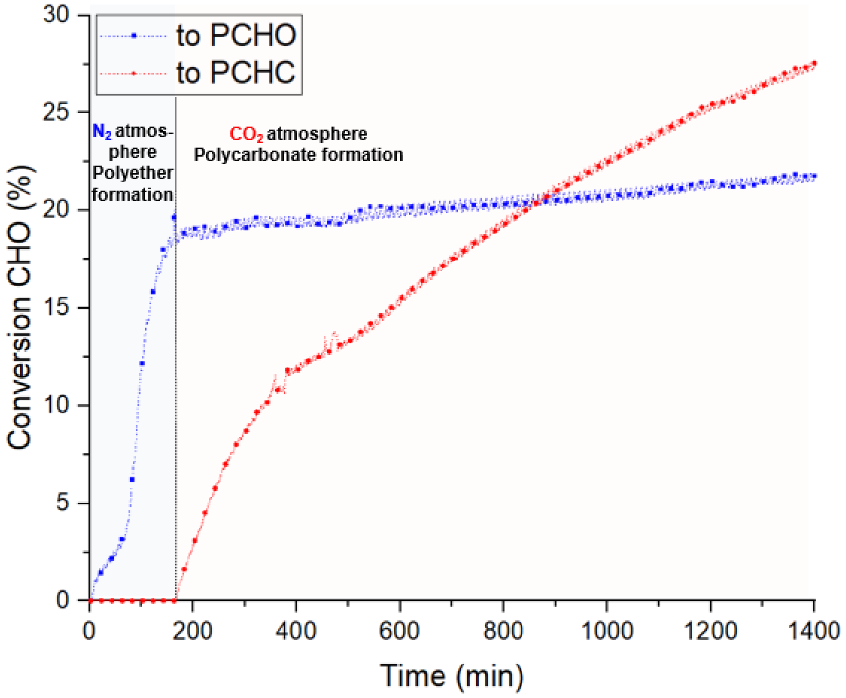

Figure S 31: Plot showing polymer conversion vs. time for a polymerization conducted with a change in gas atmosphere from nitrogen to carbon dioxide occurring after 160 mins. Polymerization conditions as per Table S1 #2.

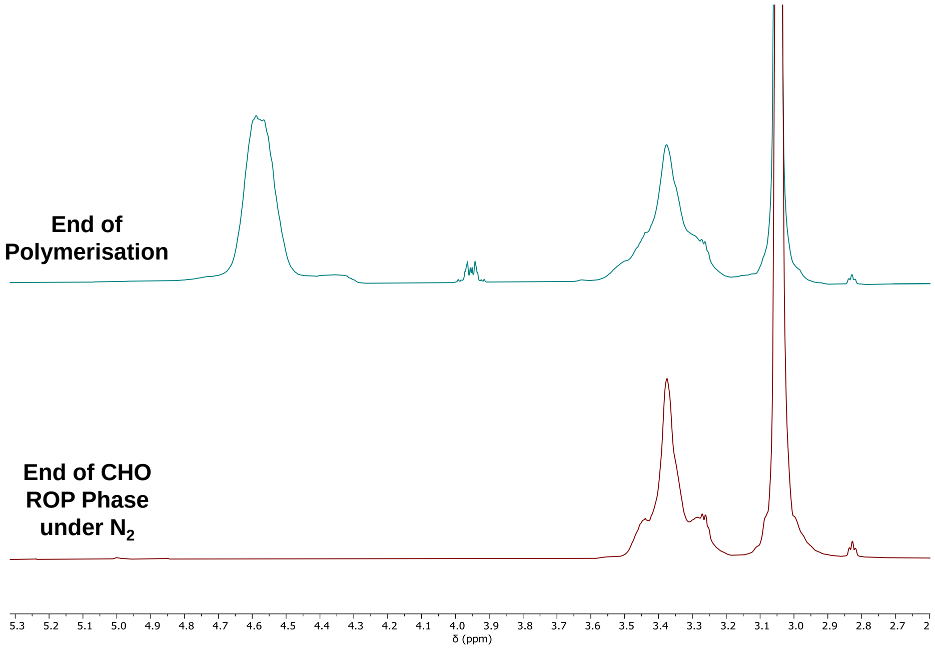

Figure S 32: ^1H NMR spectra (400 MHz, $CDCl_3$, 25°C) for aliquots removed at different stages of the switchable polymerization shown above (**Figure S 31**). The lower spectrum corresponds to the polymer produced under nitrogen and the upper spectrum to the polymer produced after the reaction was conducted under a carbon dioxide atmosphere.

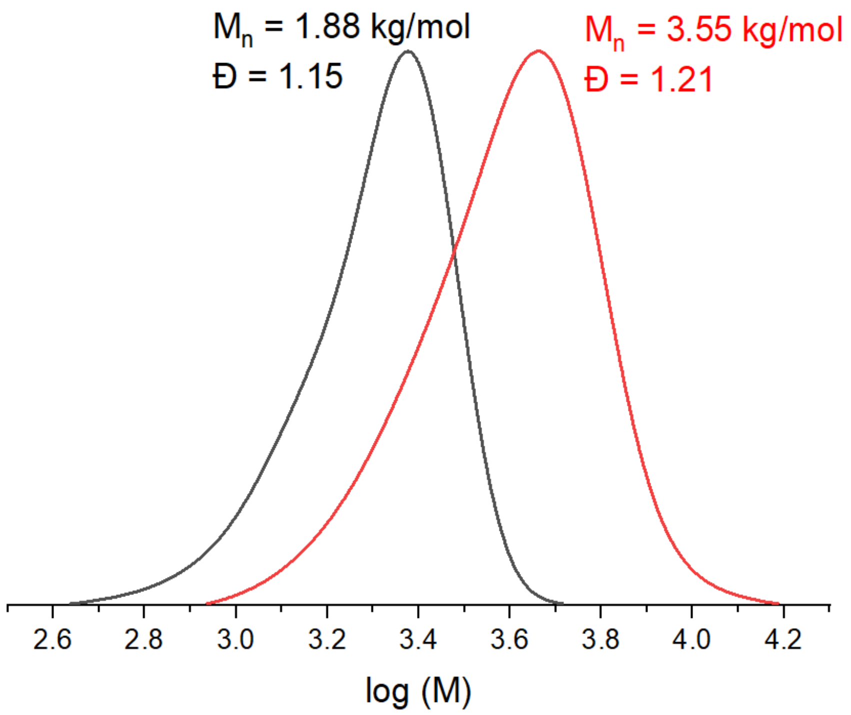

Figure S 33: GPC traces from the polymerization presented in **Figure S 31**. The black trace corresponds to the polymer produced when the reaction was conducted under nitrogen, whilst the red trace corresponds to the polymer produced after reaction under carbon dioxide.

Section S7.3: CO_2/CHO ROCOP to CHO ROP to CO_2/CHO ROCOP

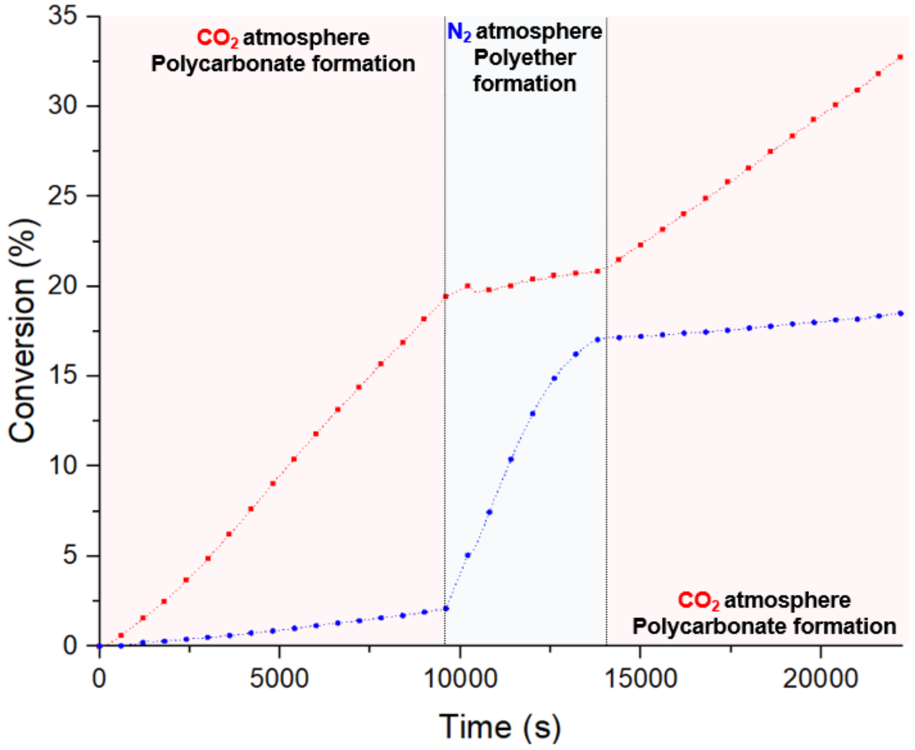

Figure S 34: Plot showing polymer conversion vs. time for a polymerization conducted with a change in gas atmosphere: from carbon dioxide to nitrogen, occurring after 10,000 s, and a change from nitrogen to carbon dioxide at 13500 s. Polymerization conditions as per Table S1 #2.

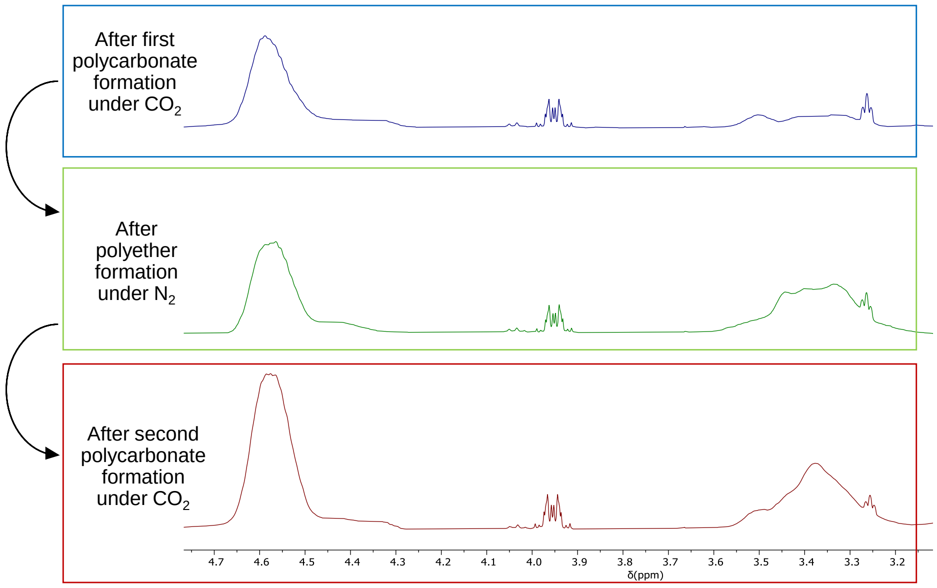

Figure S 35: ^1H NMR spectra (400 MHz, $CDCl_3$, 25°C) for aliquots removed at different stages of the switchable polymerization presented above (**Figure S 34**), before (red), after nitrogen switch (green) and after carbon dioxide switch (blue).

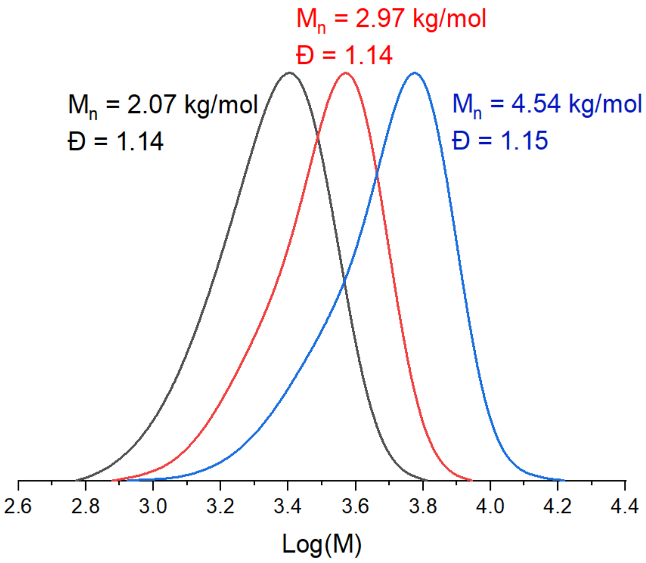

Figure S 36: GPC traces for aliquots removed for the polymerization shown above (**Figure S 34**). The black trace corresponds to the polymer produced under a carbon dioxide atmosphere (PCHC), the red trace to the copolymer produced when the gas was changed to nitrogen and the blue trace for the copolymer produced after the gas was changed back to carbon dioxide.

Section S7.4: PA/CHO ROCOP to CO_2/CHO ROCOP to CHO ROP

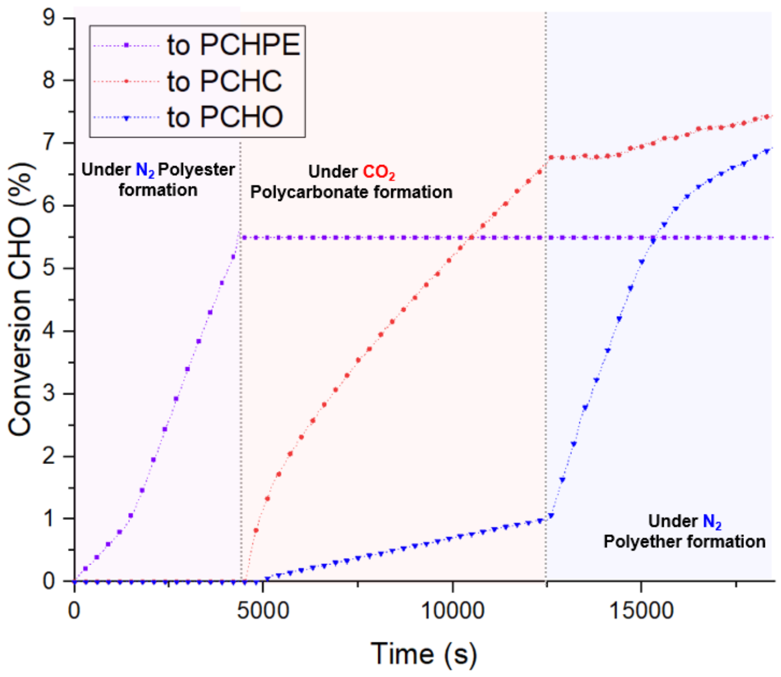

Figure S 37: Plot showing polymer conversion vs. time for a polymerization conducted with a change in gas atmosphere from carbon dioxide to nitrogen occurring after 12,500 s. Polymerization conditions: $\mathbf{Zn_2Na}$ (1 equiv), CHD (20 equiv.), PA (200 equiv.), CHO (4000 equiv.), CO_2 or N_2 (1 bar), 100 °C. During CO_2/CHO ROCOP the temperature was slowly reduced to 90 °C to reduce ether linkages in the PCHC.

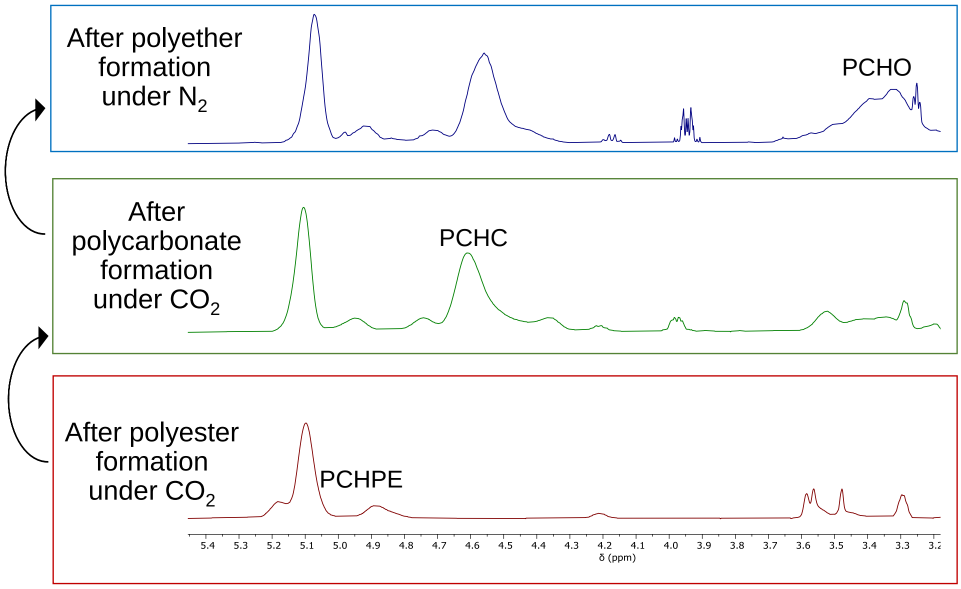

Figure S 38: ^{1}H NMR spectra (400 MHz, $CDCl_3$, 25°C) for aliquots removed at different stages during the switchable polymerisation above (**Figure S 37**).

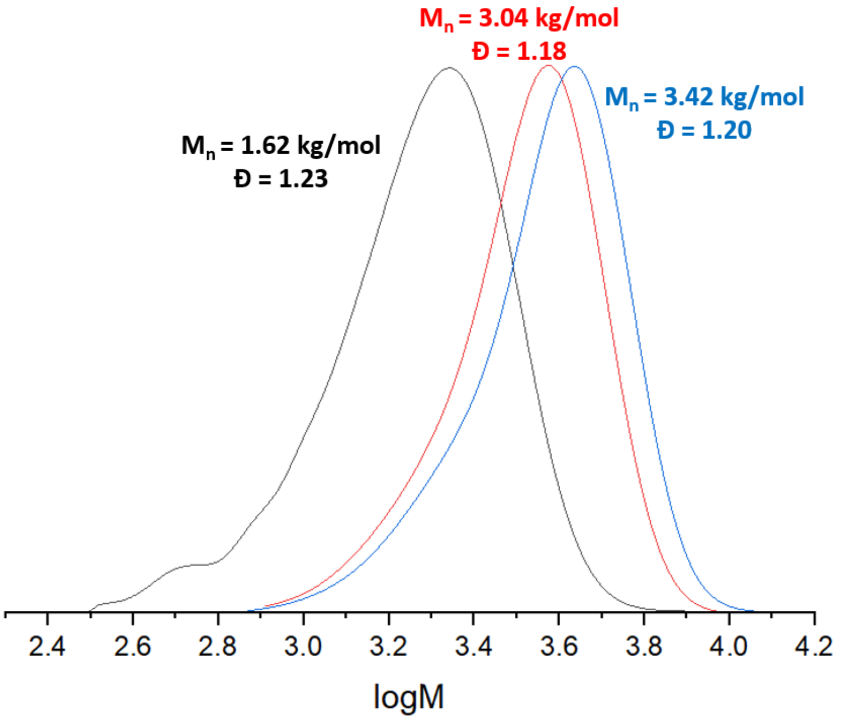

Figure S 39: GPC traces for aliquots removed after PA/CHO ROCOP stage (black), after the CO_2/CHO ROCOP stage (red) and after the CHO ROP stage (blue) (**Figure S 37**).

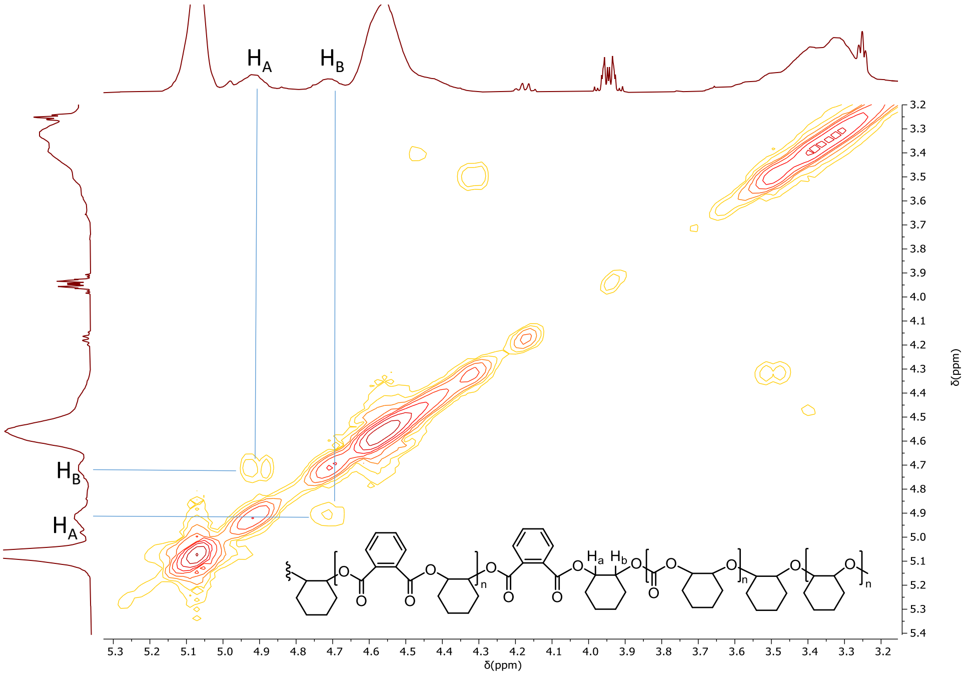

Figure S 40: *In situ* ^{1}H-^{1}H COSY NMR spectrum (400 MHz, $CDCl_3$, 25°C) showing that the ester block is joined to ether block and not vice versa (**Figure S 37**).

Section S7.5: PA/CHO ROCOP to CO_2/CHO ROCOP to CHO ROP to CO_2/CHO ROCOP

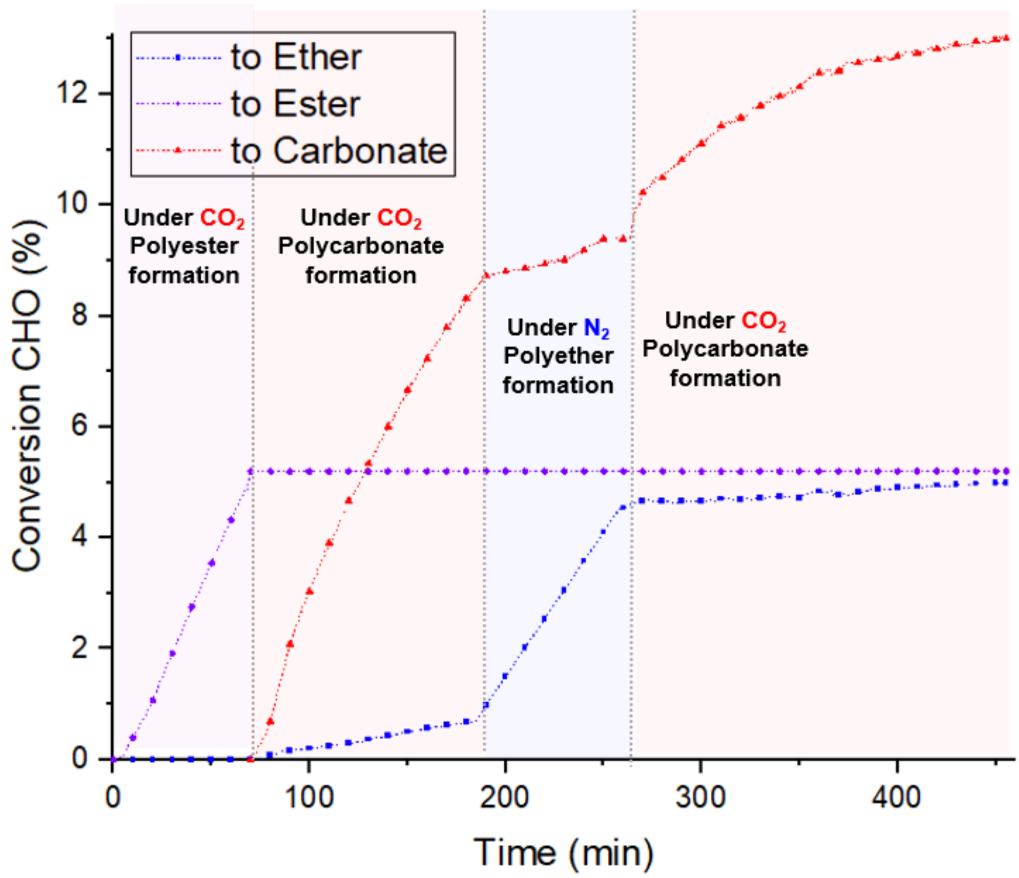

Figure S 41: Plot showing polymer conversion vs. time for a polymerization conducted with a change in gas atmosphere: from carbon dioxide to nitrogen, occurring after 190 min, and from nitrogen to carbon dioxide, at 260 min. Polymerization conditions: **Zn_2Na** (1 equiv.), CHD (20 equiv.), PA (200 equiv.), CHO (4000 equiv.), CO_2 or N_2 (1 bar), 100 °C. During CHO ROP the temperature was slowly reduced to 90°C to reduce ether linkages in the PCHC.

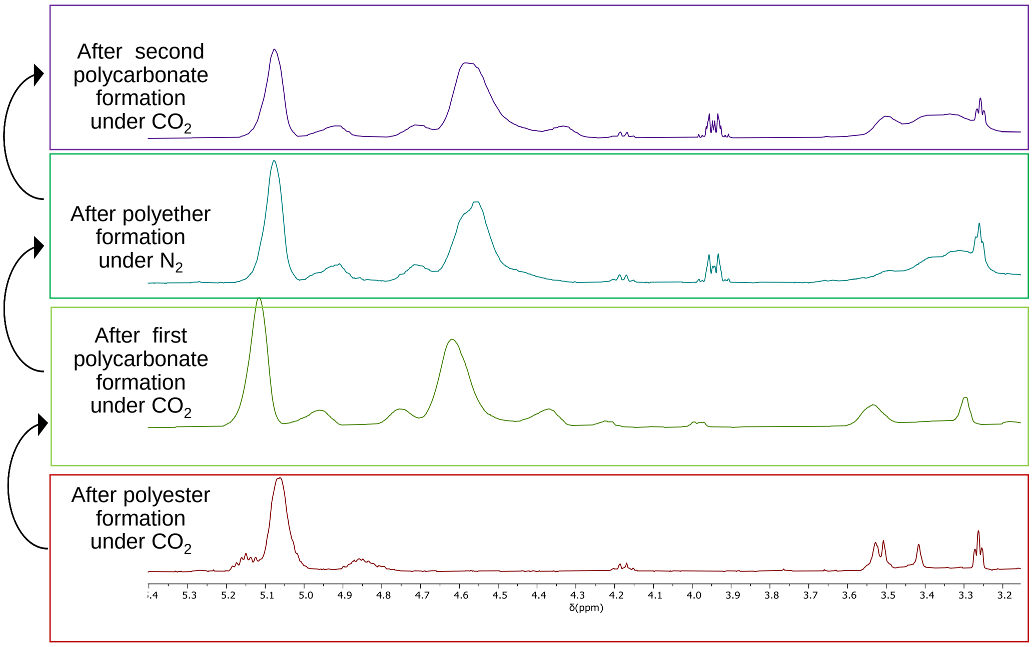

Figure S 42: In situ ^{1}H NMR spectra (400 MHz, $CDCl_3$, 25°C) of aliquots removed at different stages during switchable polymerisation (**Figure S 41**).

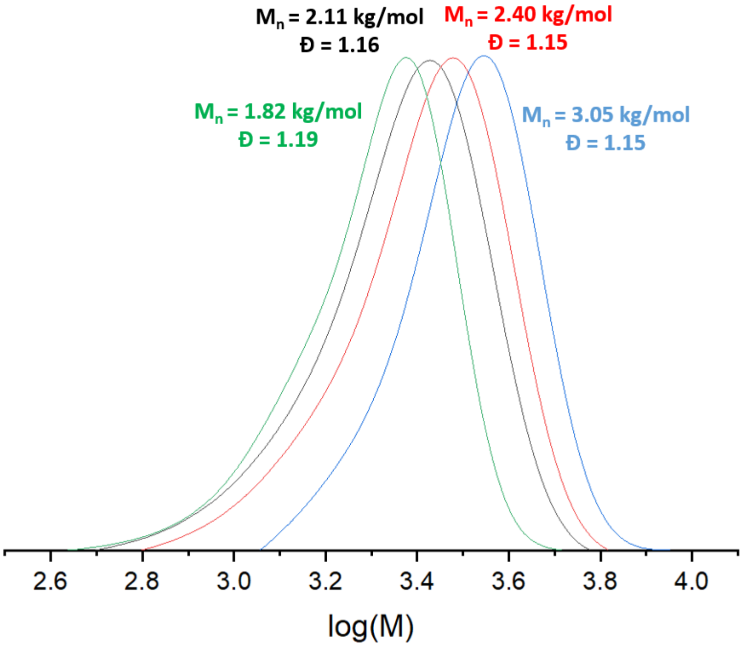

Figure S 43: GPC traces for aliquots removed after different stages of switchable catalysis. In green after the PA/CHO ROCOP stage (polyester formation), in black after the first CO_2/CHO ROCOP stage (polycarbonate formation), in red after CHO ROP stage (polyether formation), in blue after second CO_2/CHO ROCOP (polycarbonate formation) stage (**Figure S 41**).

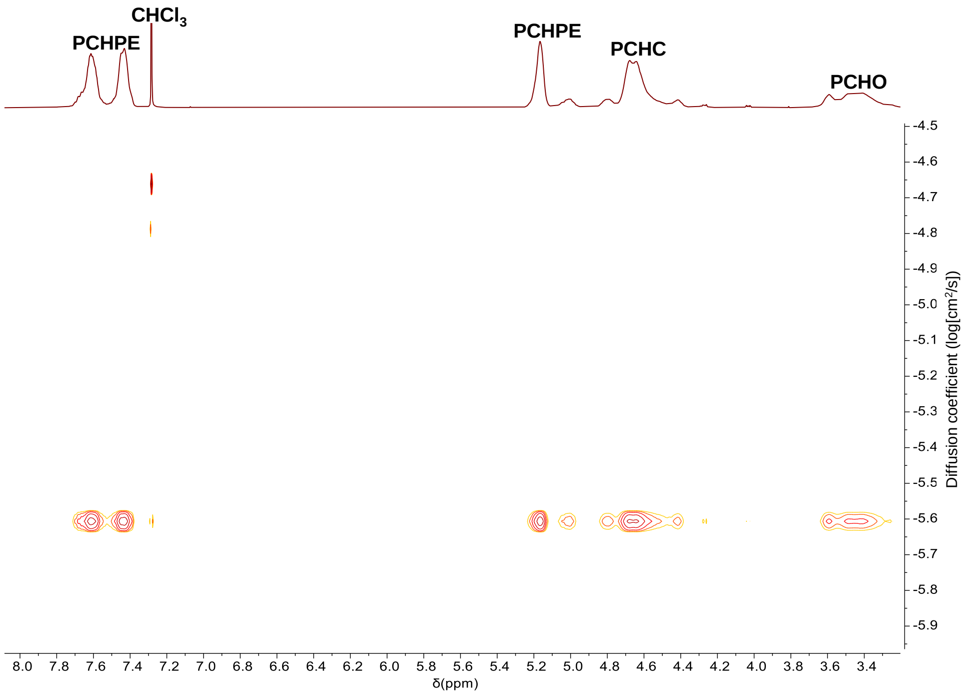

Figure S 44: Representative ^{1}H DOSY NMR spectrum (400 MHz, $CDCl_3$, 25°C) of the polymer produced from reaction above. The spectrum shows that the resonances assigned to poly(carbonate), poly(ether) and poly(ester) linkages diffuse at the same rate (**Figure S 41**).

Section S7.6: PA/CHO ROCOP to CHO ROP to CO_2/CHO ROCOP

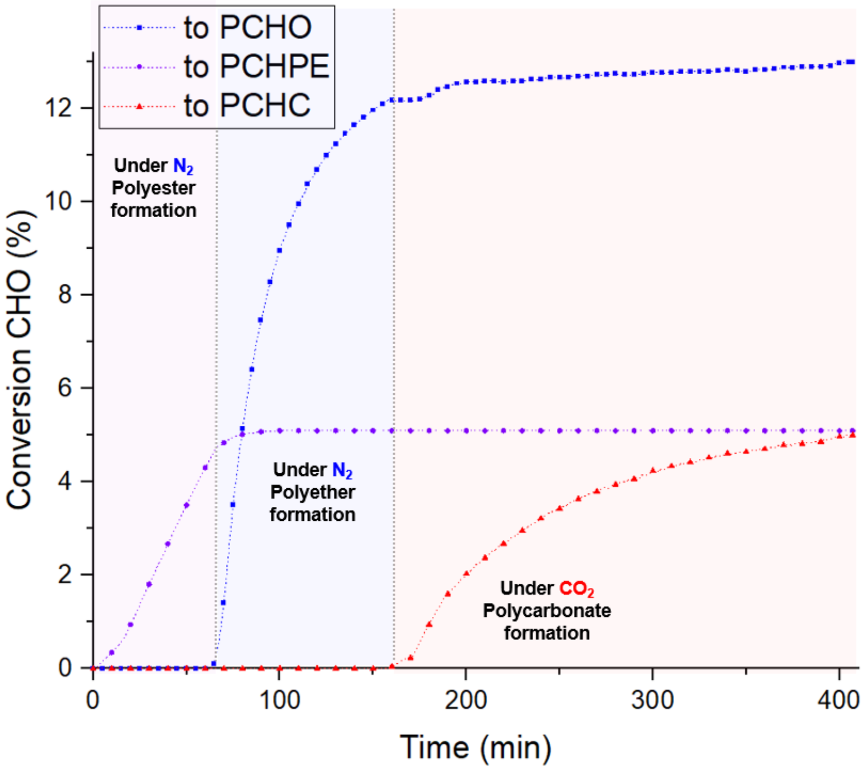

Figure S 45: Plot showing polymer conversion vs. time for a polymerization conducted with a change in gas atmosphere: from nitrogen to carbon dioxide occurring after 160 min. Polymerization conditions: **Zn_2Na** (1 equiv.), CHD (20 equiv.), PA (200 equiv.), CHO (4000 equiv.), CO_2 or N_2 (1 bar), 100 °C. During CHO ROP the temperature was slowly reduced to 90°C to reduce ether linkages in the PCHC.

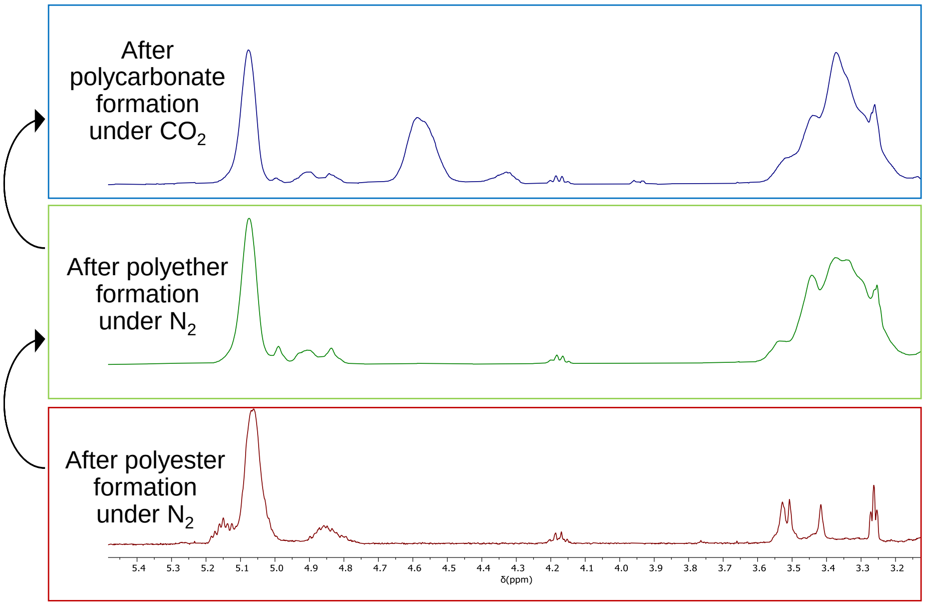

Figure S 46: In situ ^1H NMR spectra (400 MHz, $CDCl_3$, 25°C) of aliquots removed at different stages during switchable polymerisation (**Figure S 45**).

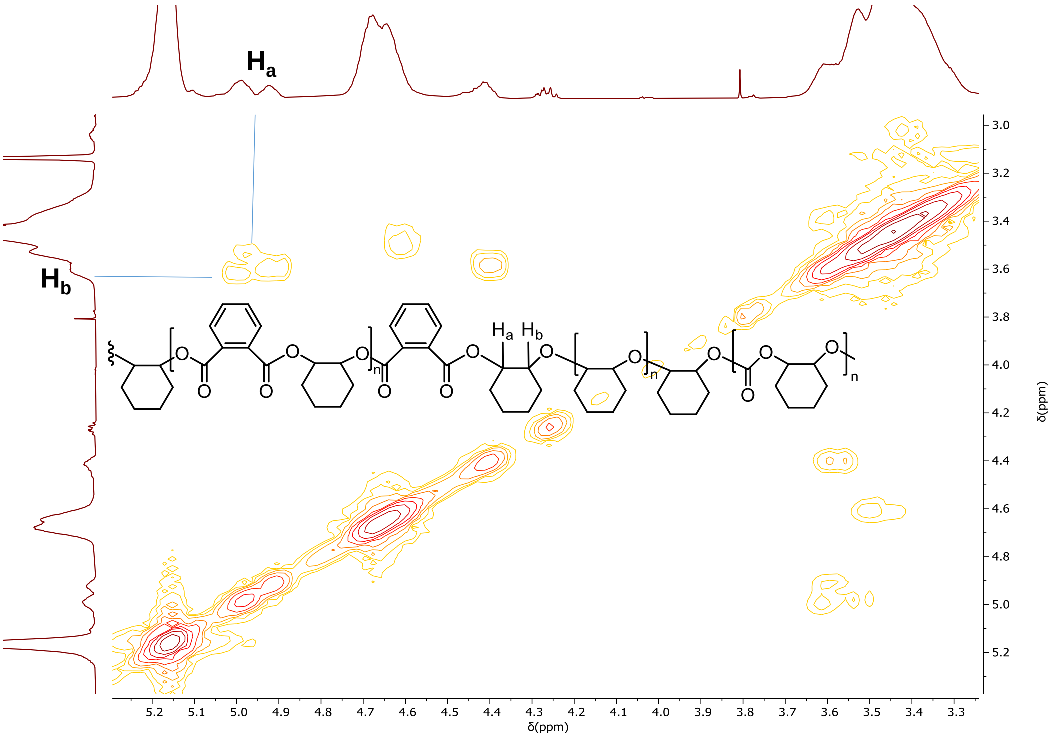

Figure S 47: ^1H-^1H COSY NMR spectrum (400MHz, $CDCl_3$, 25°C) showing that the polyester block is adjacent to the polyether block and not *vice versa* (**Figure S 45**).

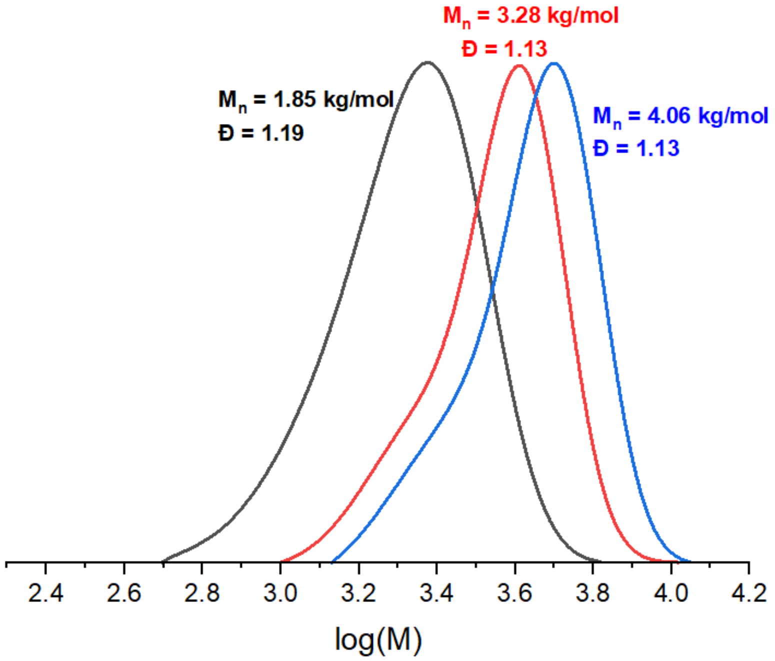

Figure S 48: GPC traces after different stages of switchable catalysis. In black after PA/CHO ROCOP stage (polyester formation), in red after CHO ROP stage (polyether formation), in blue after CO_2/CHO ROCOP stage (polycarbonate formation) (**Figure S 45**).

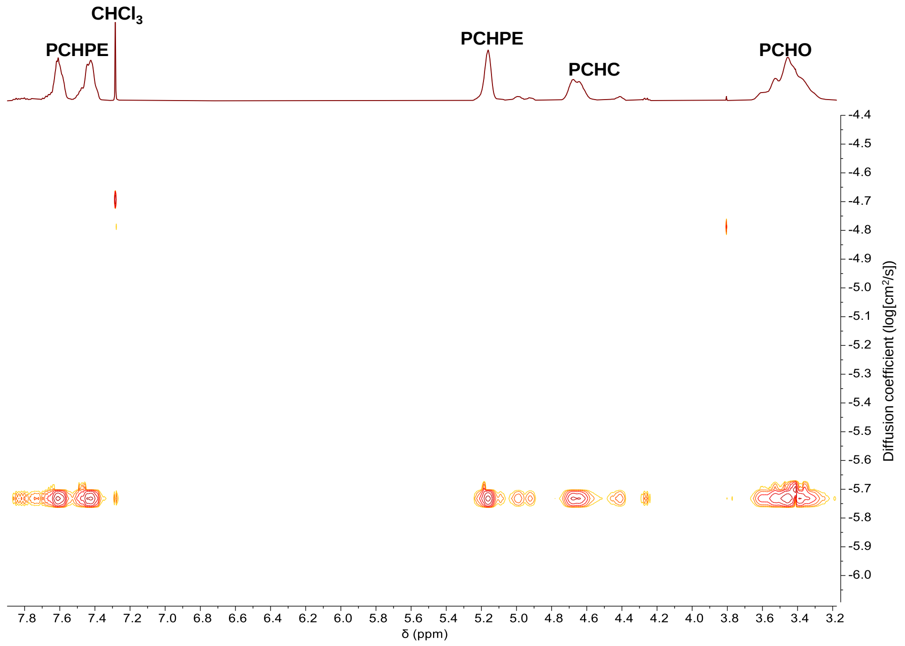

Figure S 49: Representative ^{1}H DOSY NMR spectrum (400 MHz, $CDCl_3$, 25°C) of the polymer produced according to procedure above. The plot shows that the poly(ester), -carbonate and -ether resonances diffuse at the same rate and are, therefore, attached to one another (**Figure S 45**).

Section S8: Mechanistic Investigations

Section S8.1: Arrhenius and van't Hoff analysis

Due to the fact that some CHO ROP occurs during the CO_2/CHO ROCOP at elevated temperatures, the following equation was used for Arrhenius analysis:

$$\frac{[Alkoxide\ resting\ state\ during\ ROCOP]}{[Alkoxide\ resting\ state\ during\ pure\ ROP]} = \frac{[Alkoxide\ resting\ state\ during\ ROCOP]}{[Cat_0]} = \frac{k(PCHO\ under\ CO_2)_{obs}}{k(PCHO\ under\ N_2)_{obs}} = \frac{[Alkoxide][CHO]k_{p(CHO\ ROP)}}{[Cat_0][CHO]k_{p(CHO\ ROP)}}$$

Where:

$$[Cat_0] = [Alkoxide\ resting\ state\ during\ ROCOP] + [Carbonate\ resting\ state\ during\ ROCOP]$$

Accordingly, the ratio of the observed rate for CHO ROP, during CO_2/CHO ROCOP, to the observed rate for CHO ROP, under N_2, yields the fraction of alkoxide resting state during CO_2/CHO ROCOP; and from there the fraction of carbonate resting state. For Arrhenius analysis [Cat] must be constant across the measured temperature range. However, there is an increase in alkoxide resting state as the reaction temperature is increased, due to the change in selectivity.

Hence, an approximation can be made that:

[Carbonate resting state] = [Cat_0]

Then:

$k_{obs(ROCOP)} = k_{obs(ROP+ROCOP)}[Cat_0]/[Carbonate\ resting\ state]_{(ROP+ROCOP)}$

In effect this allows determination of the observed rate for all catalyst effective during CO_2/CHO ROCOP by using a correction factor: [Cat_0]/[Carbonate resting state] which takes into account that a proportion of the [Cat_0] becomes an alkoxide resting state.

Using this method, yields the following Arrhenius plot:

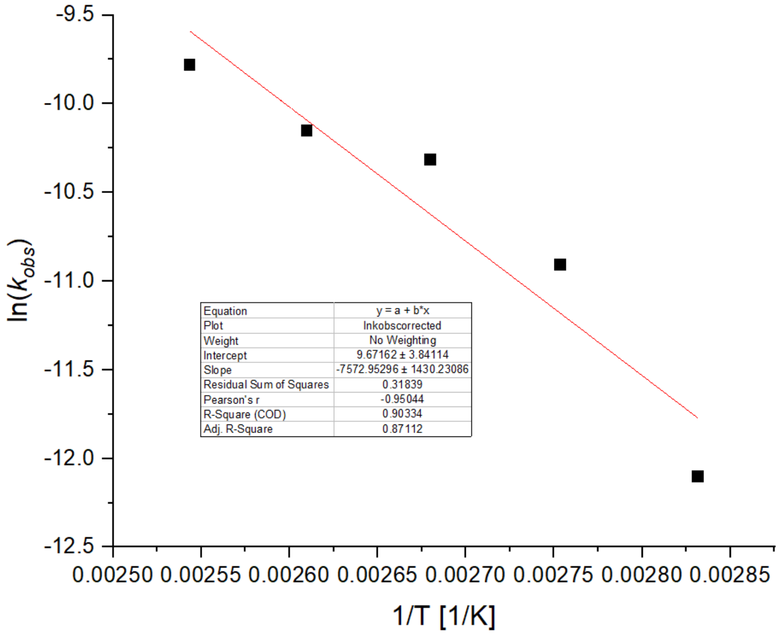

Figure S 50: Arrhenius plot for CHO/CO_2 ROCOP determined as ln(k_{obs}) vs. 1/T (K^{-1}). Polymerization conditions: 1 bar CO_2, 1 equiv. **Zn_2Na,** 20 equiv. CHD and 4000 equiv. CHO, 80-120 °C. The plot allows determination of the polymerization activation energy of E_A = 62.96 ± 11.90 kJ/mol.

With [Alkoxide during ROCOP] and [Carbonate during ROCOP] identified, it is also feasible to analyse the alkoxide-carbonate equilibrium:

$$K_{eq} = \frac{[Carbonate]}{[Alkoxide][CO_2]} = \frac{[Cat_0] - [Alkoxide]}{[Alkoxide][CO_2]}$$

To solve this relation, requires knowledge of [CO_2], and therefore the solubility of CO_2 in neat CHO over the temperature range 80 - 120 °C. The solubility data was approximated using an exponential fit (in line with Henry's law) to the published solubility of CO_2 in diethyl carbonate (which shows similar polymerization performance to reactions conducted in neat CHO).[2]

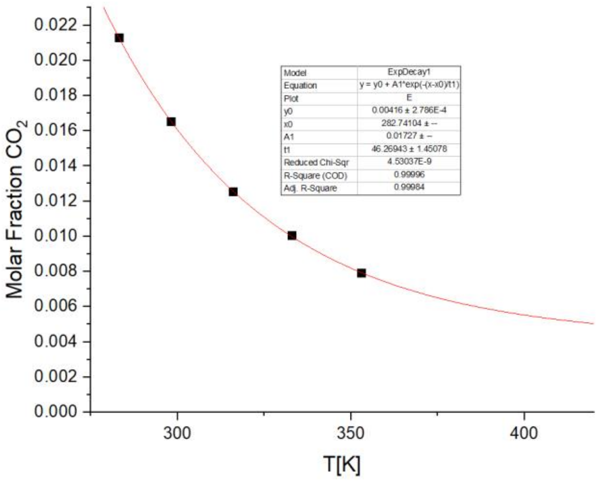

Figure S 51: Exponential Fit to solubility of CO_2 in diethyl carbonate allowing inference of [CO_2] at any particular temperature during polymerization.

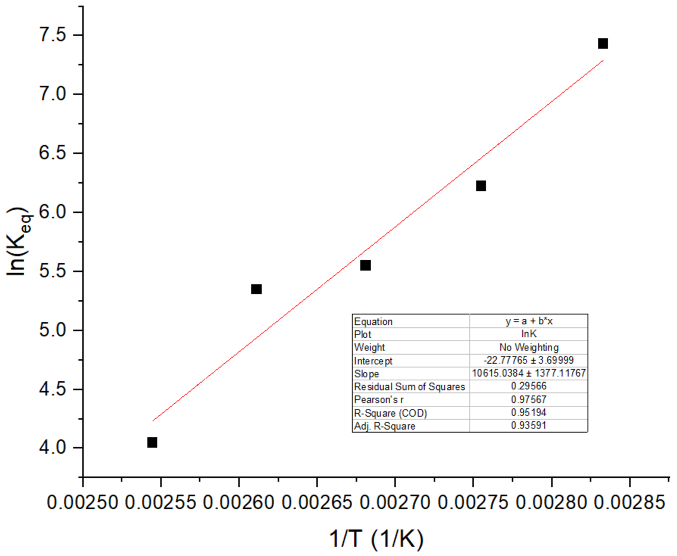

Figure S 52: Van't Hoff plot for CHO/CO_2 ROCOP. Polymerization conditions: 1 bar CO_2, 1 equiv. **Zn_2Na**, 20 equiv. CHD and 4000 equiv. CHO at 80-120 °C. The plot allows determination of: ΔH= -(88.25 ± 11.45) kJ/mol, ΔS= (189.37 ± 39.73) J/(mol*K).

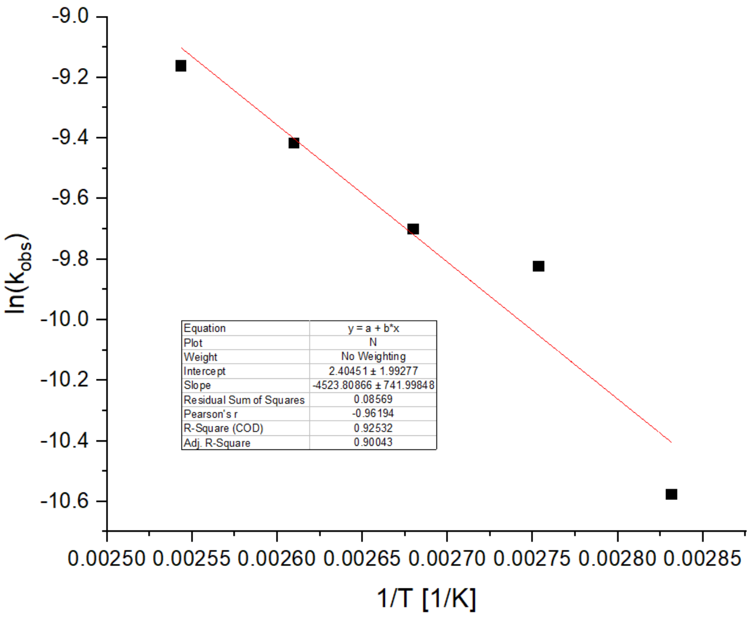

Figure S 53: Arrhenius plot for CHO ROP. Polymerization conditions: 1 bar N_2, 1 equiv. **Zn_2Na,** 20 equiv. CHD, 4000 equiv. CHO at 80-120 °C. The plot allows determination of the activation energy, E_A = (37.60 ± 6.17) kJ/mol

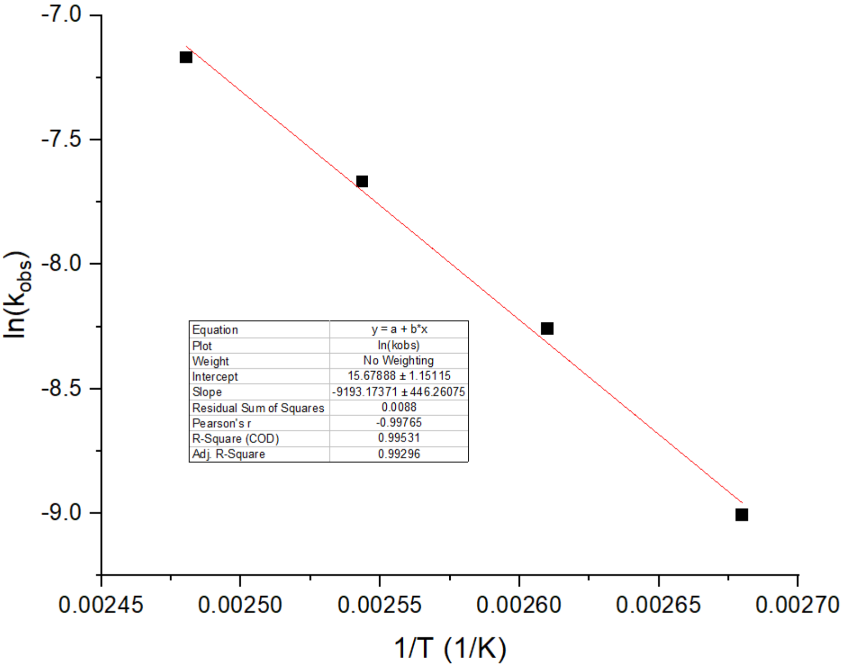

Figure S 54: Arrhenius plot for PA/CHO ROCOP. Polymerization conditions: 1 bar N_2, 1 equiv. **Zn_2Na,** 20 equiv. CHD, 100 equiv. PA, 4000 equiv. CHO at 100-130 °C. The plot allows determination of the activation energy, E_A = 76.43 ± 3.71 kJ/mol.

Section S8.2: UV-Vis Spectroscopy

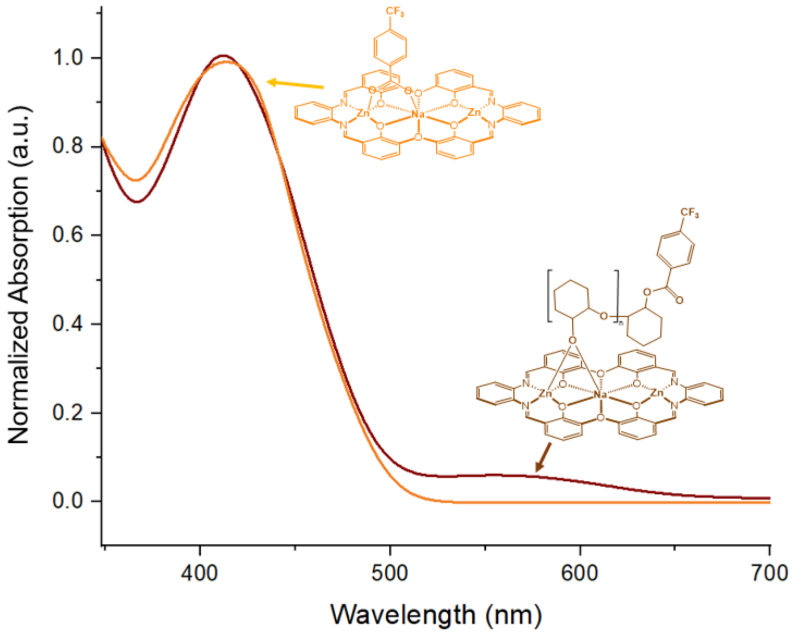

Figure S 55: UV-Vis spectra of Zn_2Na in CHO (1 to 20 equiv. CHD, 40000 equiv. CHO) before (yellow) and after (brown) heating at 120 °C for 15 min.

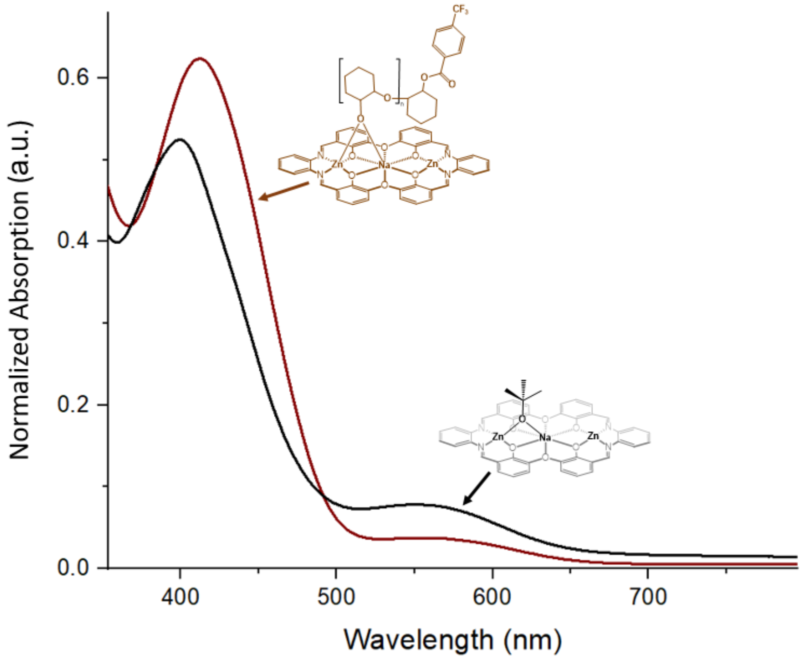

Figure S 56: UV-Vis spectra of Zn_2Na in CHO (1 to 20 equiv. CHD, 40000 equiv. CHO) after heating at 120°C for 15 min (brown) and Zn_2 after the addition of NaO^tBu (1 equiv. Zn_2, 1 equiv. NaO^tBu, 40,000 equiv. THF) (black).

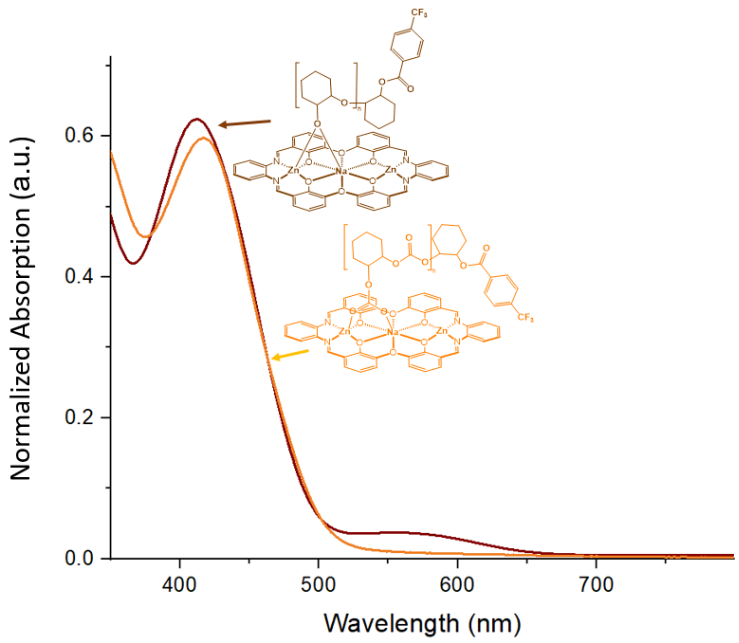

Figure S 57: UV-Vis spectra of **Zn_2Na** in CHO (1 to 20 equiv. CHD, 40,000 equiv. CHO) after heating at 120 °C for 15 min (brown) and UV-Vis spectra (orange) of **Zn_2Na** during CO_2/CHO ROCOP (1 to 20 equiv. CHD, 40,000 equiv. CHO, 1 bar CO_2, 60 min).

Section S8.3: NMR spectroscopy

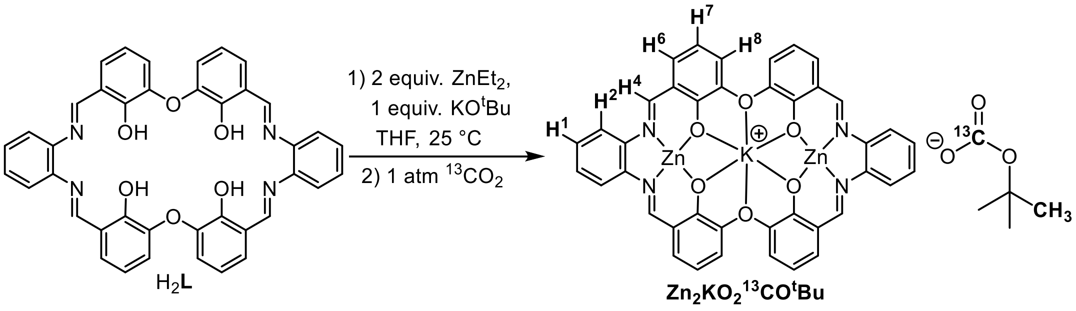

Scheme S 3: Synthesis of **$Zn_2KO_2{}^{13}CO^tBu$** and NMR assignment numbering.

$ZnEt_2$ (2.0 mg, 16.2 µmol, 2.1 equiv.) was added to a suspension of H_2**L** (5.0 mg, 7.6 µmol, 1.0 equiv.) *d*$_8$-THF (1 mL) and the resulting mixture was stirred for 10 min, at room temperature. Afterwards KO^tBu (1.5 mg, 13.4 µmol, 1.7 equiv.) was added and the resulting mixture was stirred for 10 min forming a deep brown solution. The reaction was exposed to 1 atm $^{13}CO_2$ resulting in the immediate formation of a bright yellow precipitate. The precipitate was dissolved by adding a portion of d$_6$-DMSO (0.3 mL) followed by repeating the pressurisation with $^{13}CO_2$. The obtained orange-yellow solution was analysed by NMR spectroscopy.

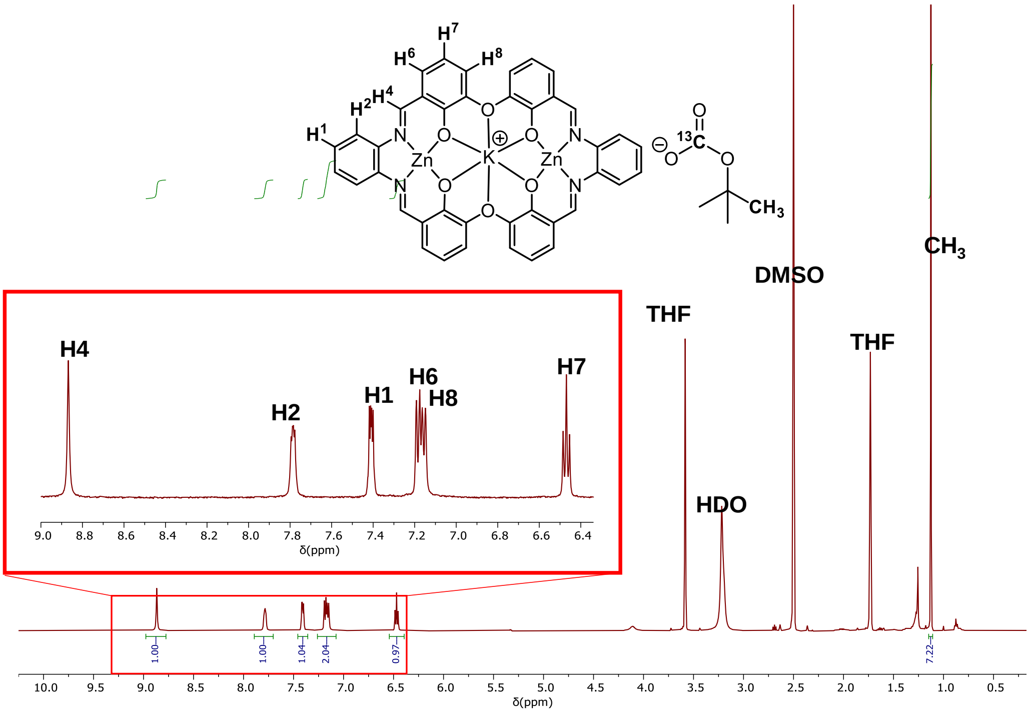

Figure S 58: ^1H NMR spectrum of **$Zn_2KO_2{}^{13}CO^tBu$** (500 MHz, d$_6$-DMSO, 25°C).

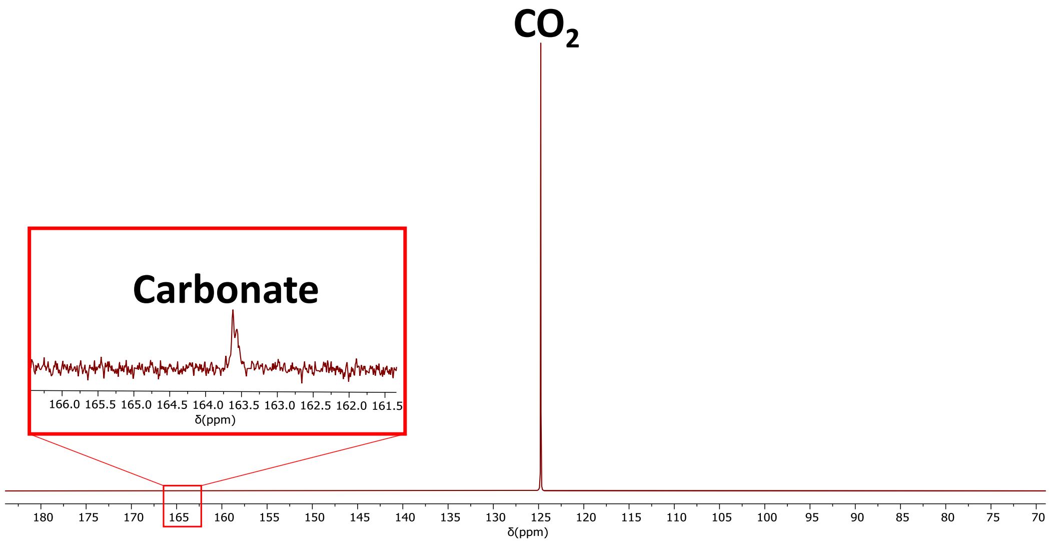

Figure S 59: $^{13}C\ \{^1H\}$ NMR spectrum of **$Zn_2NaO_2{}^{13}CO^tBu$** (176 MHz, d_6-DMSO/d_8-THF, 25°C).

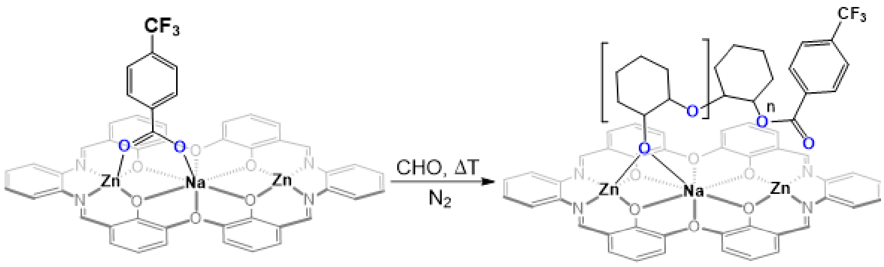

Scheme S 4: Synthesis of **RO-Zn_2Na**.

Zn_2Na (2.5mg) was heated at 100 °C in CHO (2 mL, 1 equiv. **Zn_2Na**, 4000 equiv. CHO). All volatiles were removed under vacuum and the remaining solid was dissolved in dry d_8-THF and analysed by NMR spectroscopy, note that only a broad and undefined spectrum was observed.

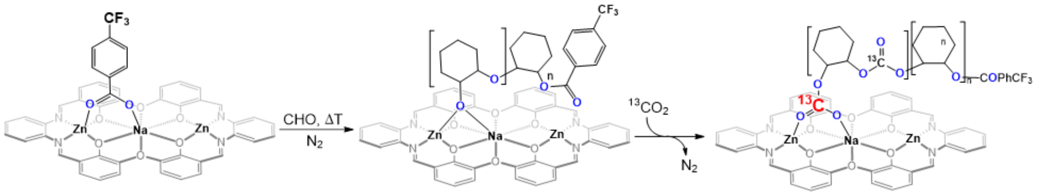

Scheme S 5: Synthesis of **ROCO$_2$-Zn_2Na**.

Zn_2Na (2.5 mg, 1 equiv.) was heated at 100 °C for 30 min, in CHO (2 mL, 4000 equiv.) and with CHD (6 mg, 20 equiv.) inside a J-Youngs NMR tube containing a d_8-toluene lock capillary and using $(MeOPh)_3P$ as an internal standard. Afterwards the sample was cautiously evacuated, before completely cooling to aid degassing and $^{13}CO_2$ was added at 1.5 atm pressure. Note that the $^{13}CO_2$ insertion does also occur readily at room temperature, but requires a greater number of de-gassing cycles; the conditions presented here are preferable due to the high cost of $^{13}CO_2$.

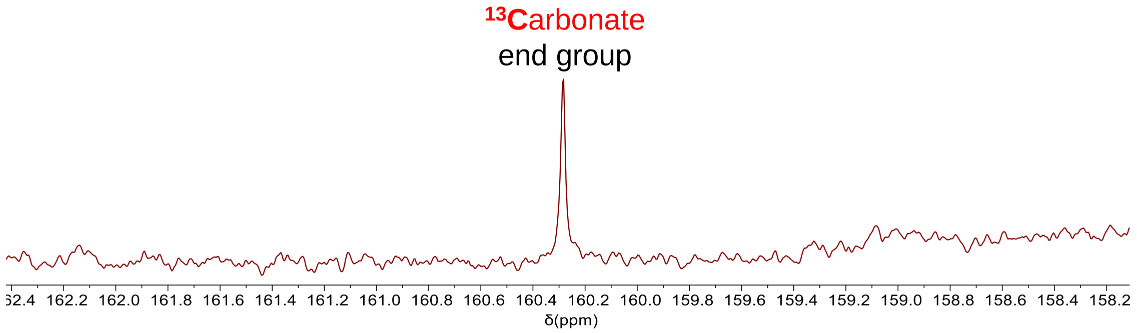

Figure S 60: $^{13}C\{^{1}H\}$ NMR spectrum of **$ROCO_2$-Zn_2Na** carbonate group (176 MHz, CHO with d_8-toluene lock capillary containing $(MeOPh)_3P$ standard, 25 °C) of the catalytic carbonate intermediate (i.e. catalyst resting state during CO_2/CHO ROCOP).

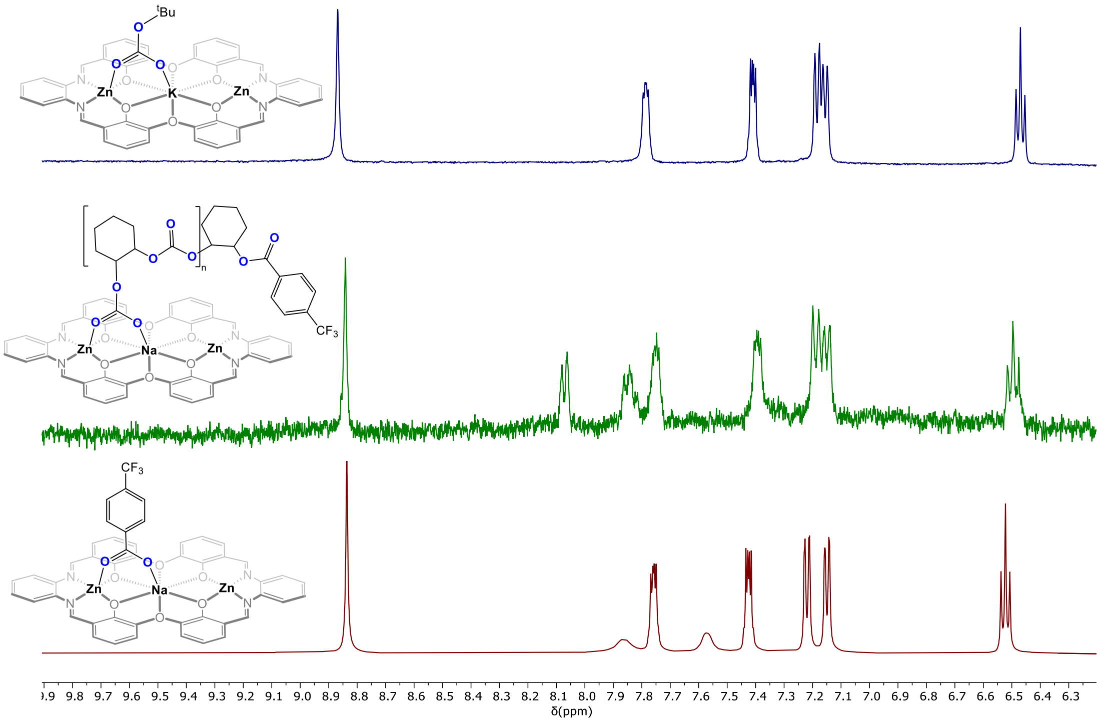

Figure S 61: Comparison of the ^{1}H NMR spectra (400 MHz, d_6-DMSO, 25°C) of (top) **$Zn_2KO_2{}^{13}CO^tBu$**, (middle) an aliquot obtained from CHO/CO_2 ROCOP with conditions according to Table and (bottom) **Zn_2Na**.

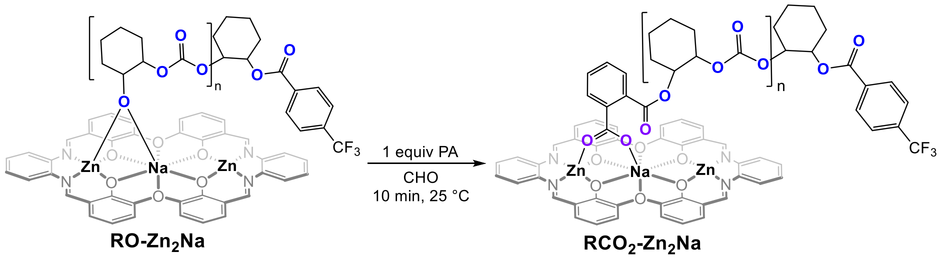

Scheme S 5: Synthesis of **RCO_2-Zn_2Na**.

Zn_2Na (2.5 mg, 1 equiv.) was heated at 100 °C in CHO (2 mL, 4000 equiv. CHO). The solution was cooled to room temperature and transferred to a N_2 filled glovebox where phthalic anhydride (0.37 mg, 1 equiv., from a stock solution in CHO) was added. The resulting solution was stirred for 1 h, at room temperature. All volatiles were removed under vacuum and the remaining solid was dissolved in dry d_8-THF and analysed by NMR spectroscopy.

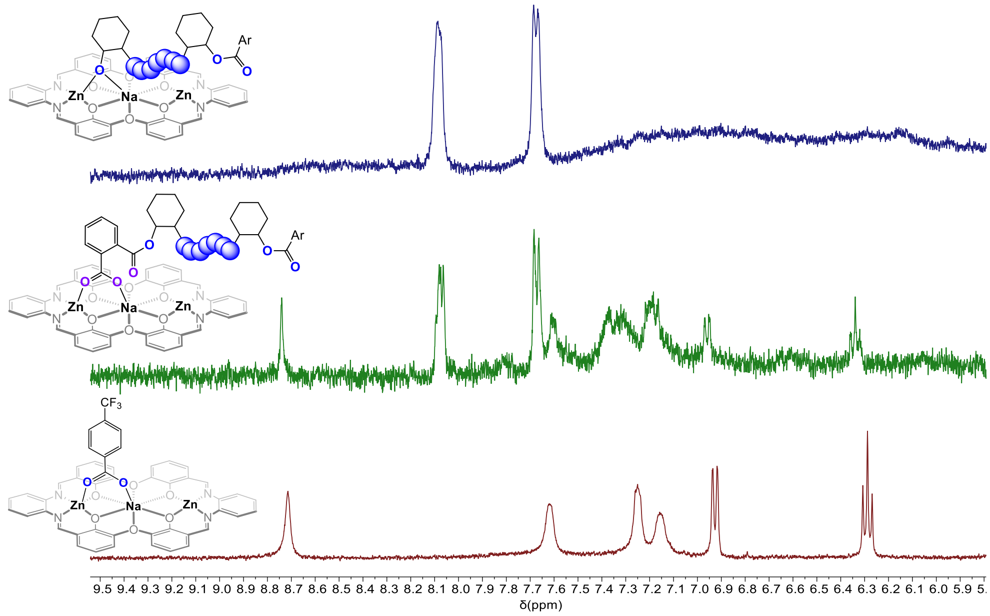

Figure S 62: Comparison of the ^{1}H NMR spectra (500 MHz, d_8 -THF, 25°C) of: (top) **RO-Zn_2Na**, (middle) **RCO_2-Zn_2Na**, (bottom) **Zn_2Na**.

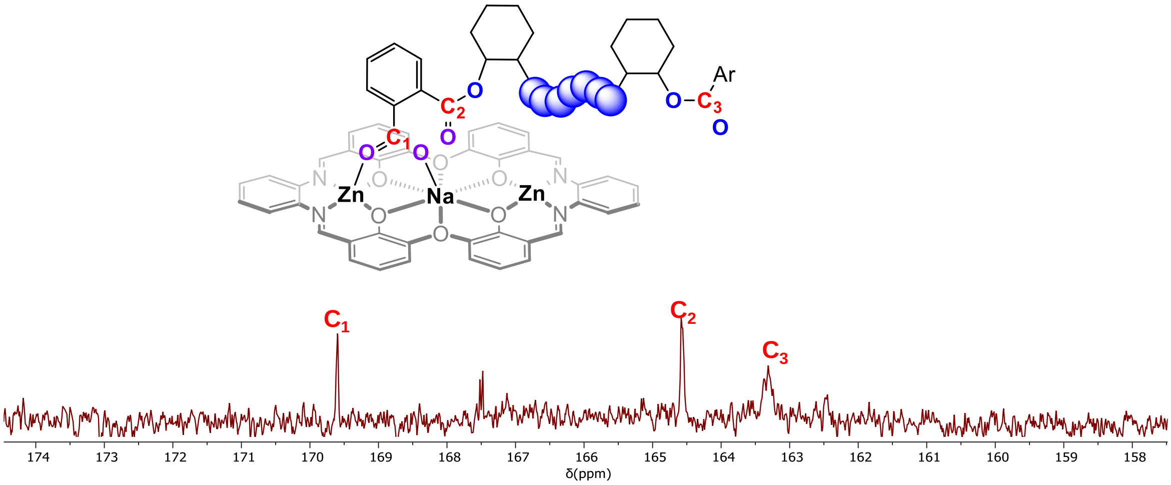

Figure S 63: Carbonyl region of the ^{13}C NMR spectrum (176 MHz, d_8 -THF, 25°C) of **RCO_2-Zn_2Na**.

Section S9: Synthesis of Zn_2K and its Polymerisation Kinetics

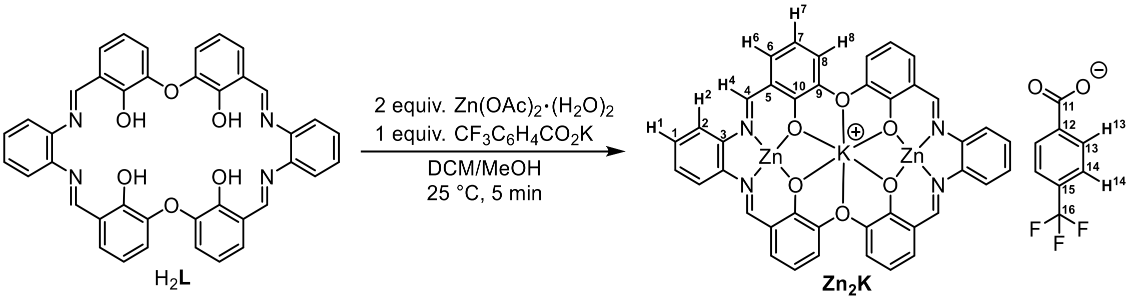

Scheme S 6: Synthesis of **Zn₂K** and NMR assignment numbering.

Synthesis of Zn_2K: A solution of $Zn(OAc)_2{\cdot}(H_2O)_2$ (33.3 mg, 151 µmol) and $CF_3C_6F_4CO_2K$ (17.1 mg, 75 µmol) in MeOH (5 mL) was added to a solution of H_2**L** (50.0 mg, 75 µmol) in DCM (5mL). The resulting solution was left unperturbed for 5 min. Afterwards all volatiles were removed, in vacuo, yielding a semi-solid which was washed with Et_2O (20 mL). In order to remove any acetic acid by-product, the crude material was suspended in toluene (20 mL) which was afterwards removed in vacuo. This process was repeated, yielding **Zn_2K**$\cdot 2H_2O$ as an orange powder (85.0 mg, 73 µmol, 97%).

^{1}H NMR (500 MHz, d_6-DMSO): δ 8.86 (s, 1H, H4), 7.92 (s, 0.5H, H13),H4, 7.78 (dd, *J* = 6.1, 3.4 Hz, 1H, H2), 7.61 (s, 0.5H, H14), 7.44 (dd, *J* = 6.0, 3.3 Hz, 1H, H1), 7.21 (d, *J* = 8.0 Hz, 1H, H6), 7.13 (d, *J* = 7.7 Hz, 1H, H8), 6.51 (t, *J* = 7.8 Hz, 1H, H7).

^{13}C {^{1}H} NMR (126 MHz, d_6-DMSO): δ 168.33, 164.27, 163.26, 148.40, 140.90, 130.67, 130.21, 128.10, 124.88, 124.81 (d, *J* = 272.3 Hz), 121.20, 120.60, 117.71, 112.10.

^{19}F {^{1}H} NMR (470 MHz, d_6-DMSO): δ -60.94.

HRESI-MS (positive ionization mode) m/z = (M- $CF_3C_6F_4CO_2)^+$ calculated 822.9910 found 822.9917

Elemental Analysis (**Zn_2K**$\cdot 2H_2O$, $C_{48}H_{32}F_3N_4O_{10}KZn_2$) calculated C 54.8%, H 3.1%, N 5.3%; found C 54.8%, H 2.8%, N 5.3%

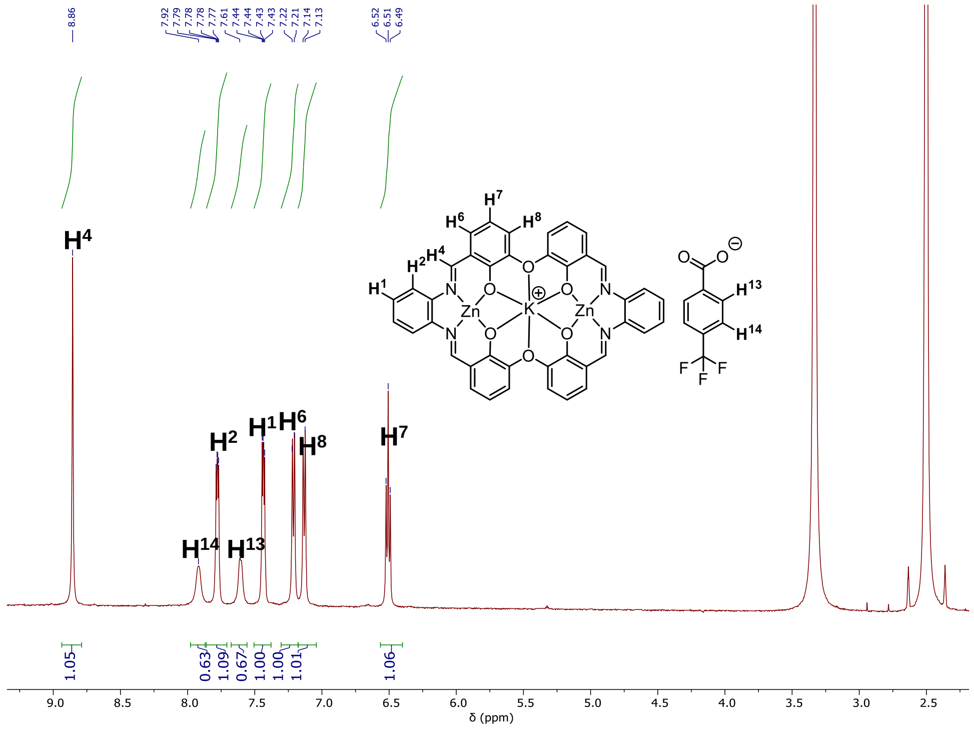

Figure S 64: ^{1}H NMR spectrum (500 MHz, d_6-DMSO, 25 °C) of **Zn_2K**.

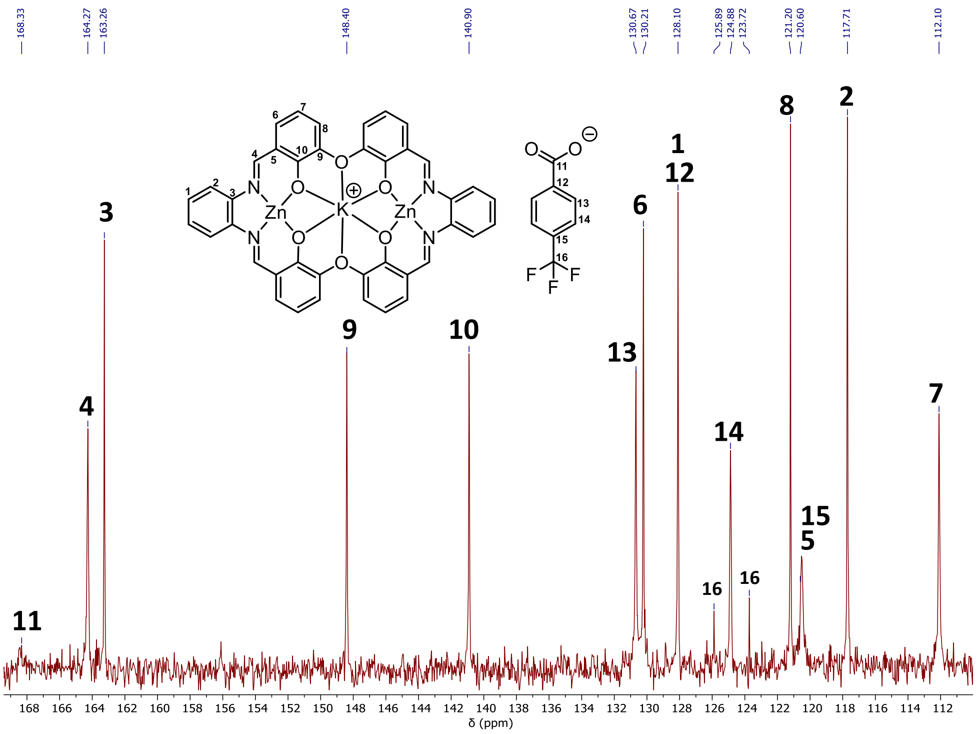

Figure S 65: $^{13}C\{^{1}H\}$ NMR spectrum (126 MHz, d_6-DMSO, 25°C) of **Zn_2K**.

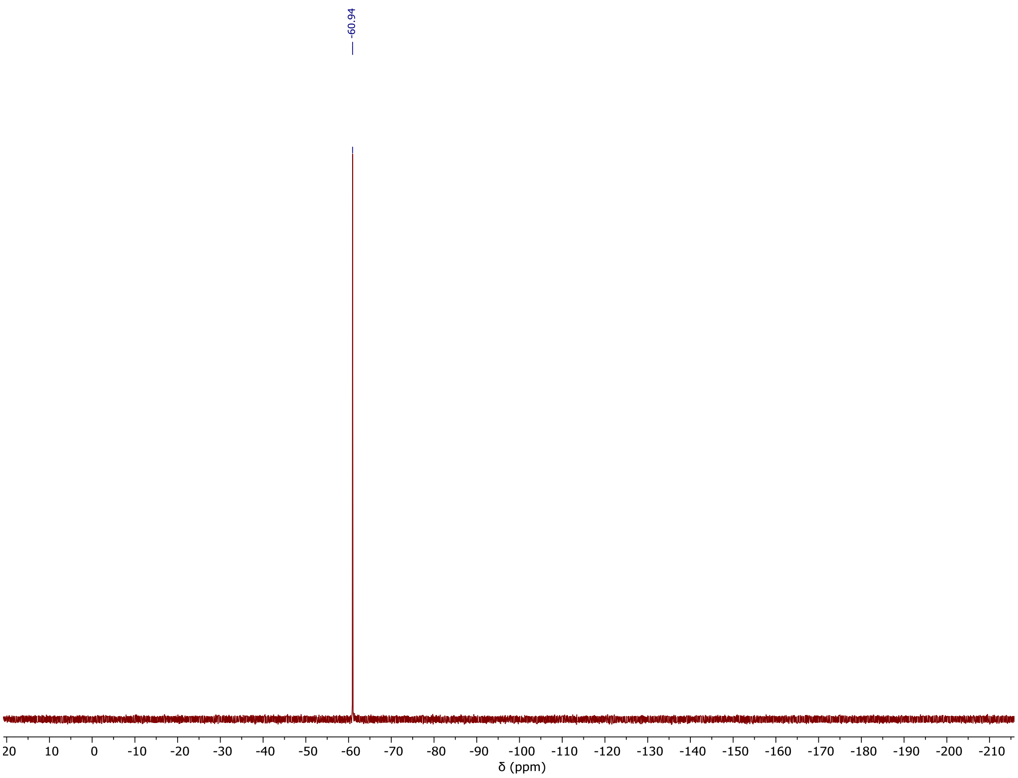

Figure S 66: ^{19}F{^{1}H} NMR spectrum (470 MHz, d6-DMSO, 25°C) of **Zn_2K**.

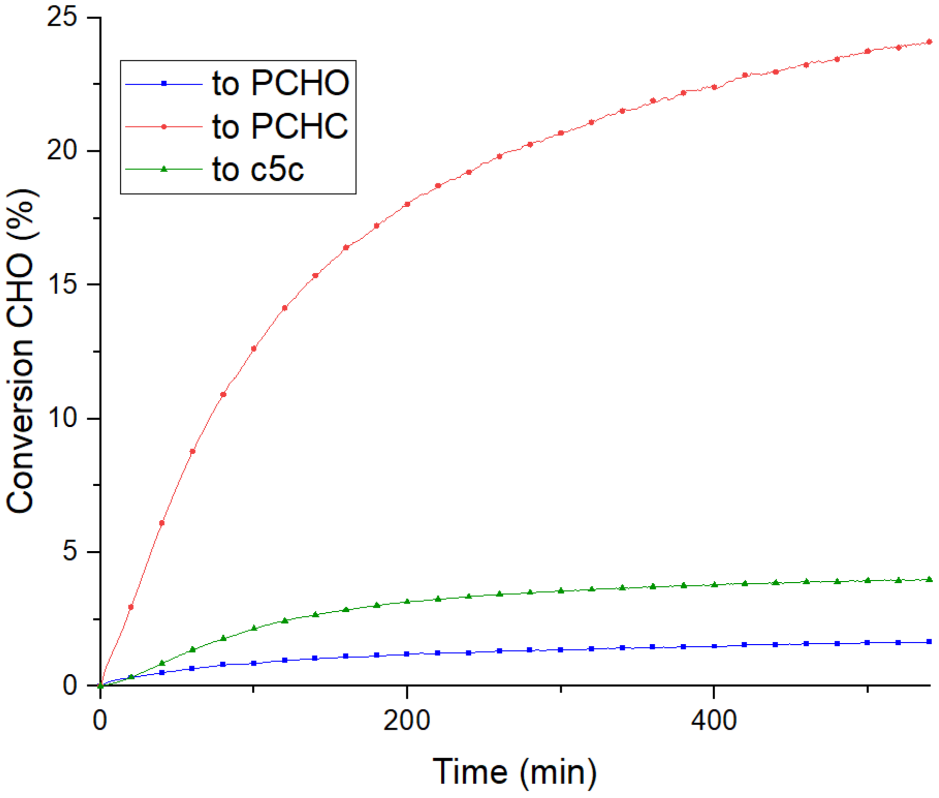

Figure S 67: Conversion vs. time plot for CHO/CO_2 ROCOP. Polymerization conditions: 1 bar CO_2, 1 equiv. **Zn_2K**, 20 equiv. CHD and 4000 equiv. CHO at 100 °C.

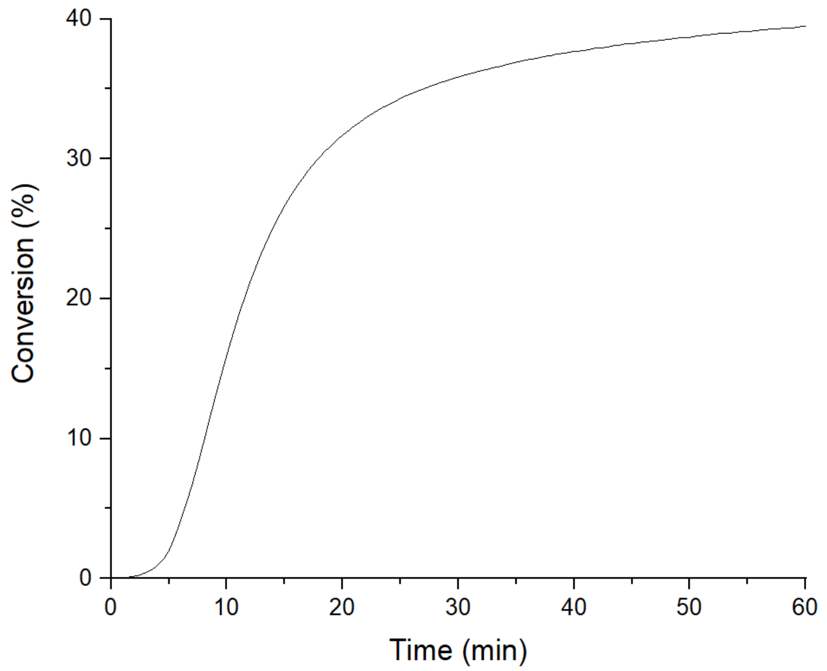

Figure S 68: Conversion vs. time plot for CHO ROP. Polymerization conditions: 1 bar N_2, 1 equiv. **Zn_2K**, 20 equiv. CHD and 4000 equiv. CHO at 100 °C. TOF = 7379 h^{-1}.

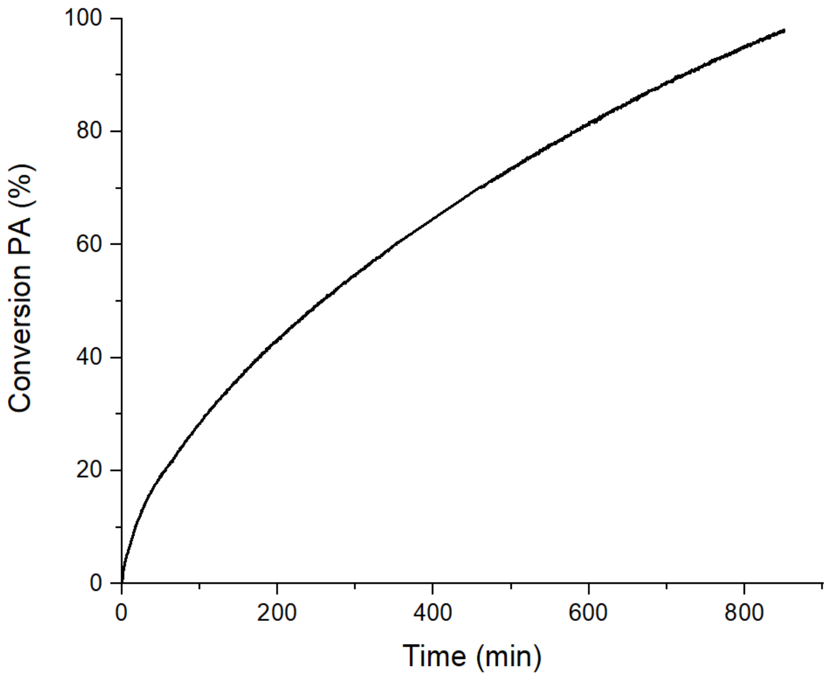

Figure S 69: Conversion vs. time plot for PA/CHO ROCOP. Polymerization conditions: 1 bar N_2, 1 equiv. **Zn_2K**, 20 equiv. CHD, 200 equiv. PA and 4000 equiv. CHO at 100 °C. TOF = 14 h^{-1}.

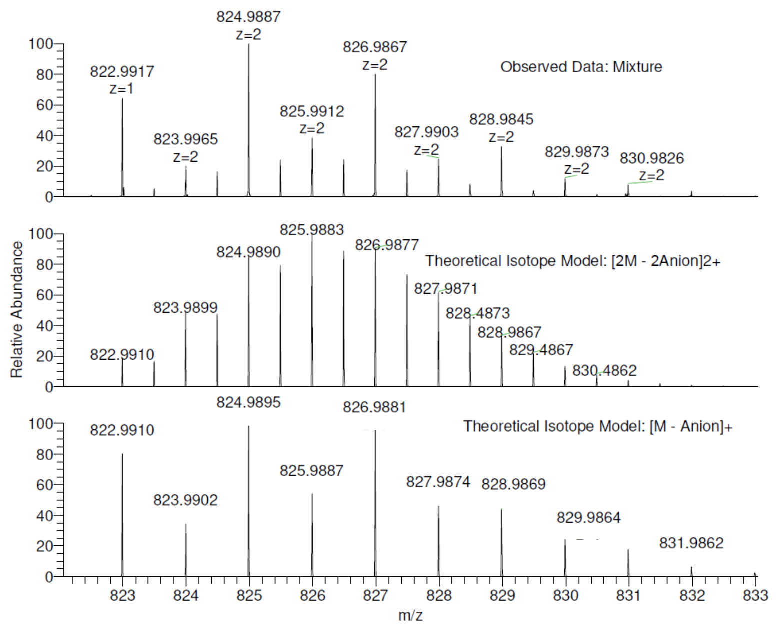

Figure S 70: High resolution ESI mass spectrum of **Zn_2K**. The molecular ion (**M** - $CF_3C_6F_4CO_2$)$^+$ results from loss of a $CF_3C_6F_4CO_2$ co-ligand during ionization.

Section S10: Synthesis of Zn_2Li and its Polymerisation Kinetics

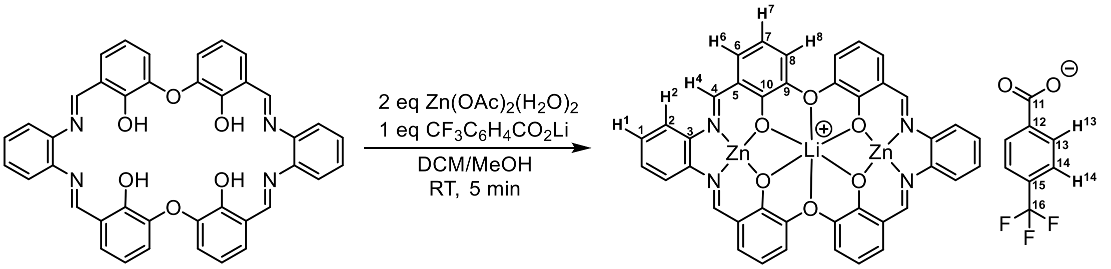

Scheme S 6: Synthesis of **Zn_2Li** and NMR assignment numbering.

Synthesis of Zn_2Li: A solution of $Zn(OAc)_2{\cdot}(H_2O)_2$ (33.3 mg, 151 µmol) and $LiCO_2C_6H_4CF_3$ (14.6 mg, 75 µmol) in MeOH (5 mL) was added to a solution of H_2**L** (50.0 mg, 75 µmol) in DCM (5 mL). The resulting solution was left unperturbed for 5 min during which a bright yellow precipitate formed which was isolated by centrifugation, washed with Et_2O (20 mL) and dried, in vacuo, to yield **Zn_2Li** $3H_2O$ as a yellow powder (66 mg, 68 µmol, 90%).

^{1}H NMR (400 MHz, DMSO) δ 8.74 (s, 1H, H4), 7.97 (d, *J* = 8.2 Hz, 0.5H, H14), 7.68 (dd, *J* = 6.3, 3.5 Hz, 1H, H2), 7.58 (d, *J* = 9.0 Hz, 0.5H, H1), 7.40 (dd, *J* = 5.8, 3.5 Hz, 1H, H6), 7.11 (dd, *J* = 7.7, 1.6 Hz, 1H, H8), 7.02 – 6.64 (m, 2H, H8), 6.41 (t, *J* = 7.6 Hz, 1H, H7).

No **^{13}C {^{1}H} NMR** could be obtainted due to the low solubility of Zn_2Li

^{19}F {^{1}H} NMR (470 MHz, d_6-DMSO): δ -60.59**.**

^{7}Li NMR (156 MHz, DMSO): δ -1.00.

HRESI-MS (positive ionization mode) m/z = (**M** - $LiCO_2C_6H_4CF_3$+ Na)$^+$ calculated 809.0141 found 809.0144

Elemental Analysis (**Zn_2Li** $3H_2O$; $C_{48}H_{34}F_3LiN_4O_{11}Zn_2$) calculated C 55.6%, H 3.3%, N 5.4% found C 55.4%, H 3.0%, N 5.3%

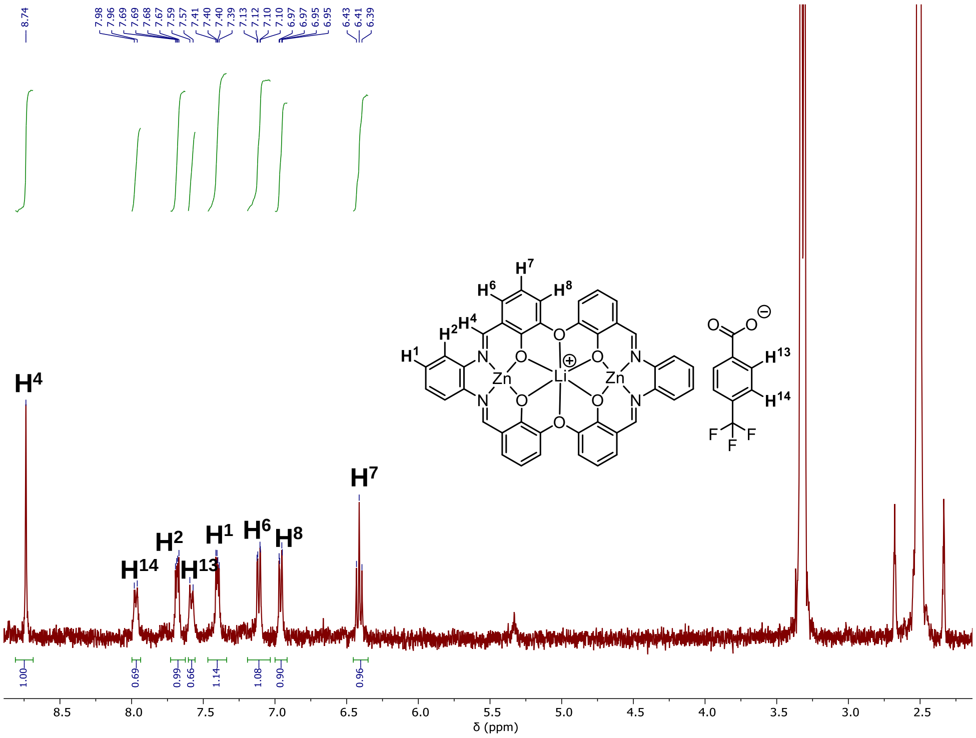

Figure S 71: ^{1}H NMR spectrum (500 MHz, d_6-DMSO, 25°C) of **Zn_2Li**.

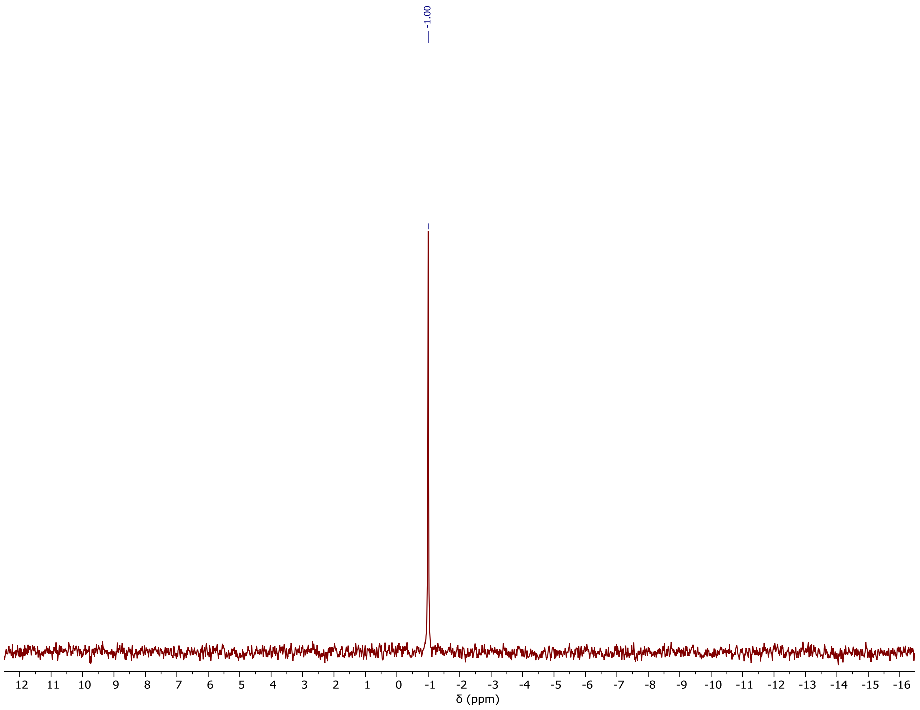

Figure S 72: $^{7}Li\{^{1}H\}$ NMR spectrum (156 MHz, DMSO, 25°C) of **Zn_2Li**.

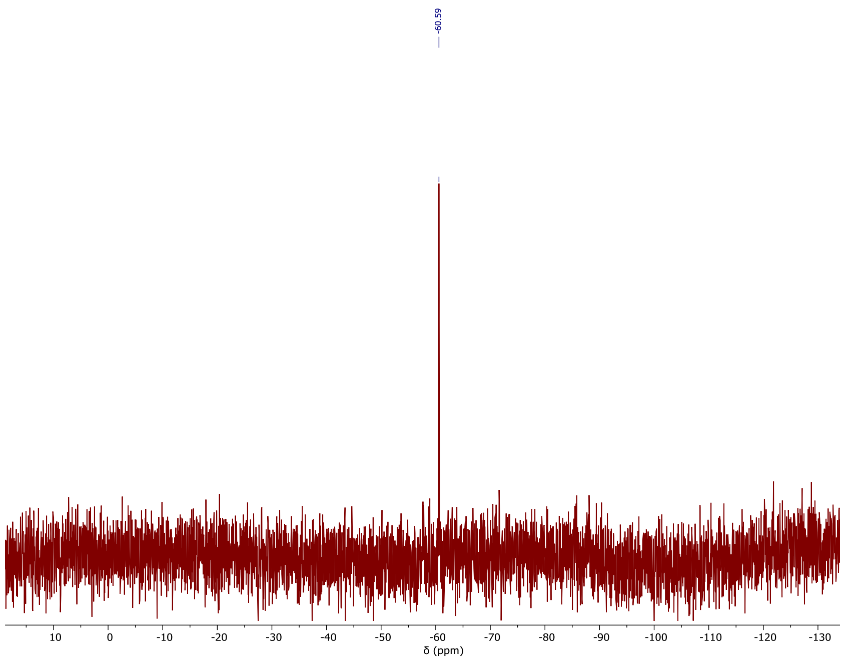

Figure S 73: $^{19}F\{^{1}H\}$ NMR spectrum (470 MHz, d_6-DMSO, 25°C) of **Zn_2Li**.

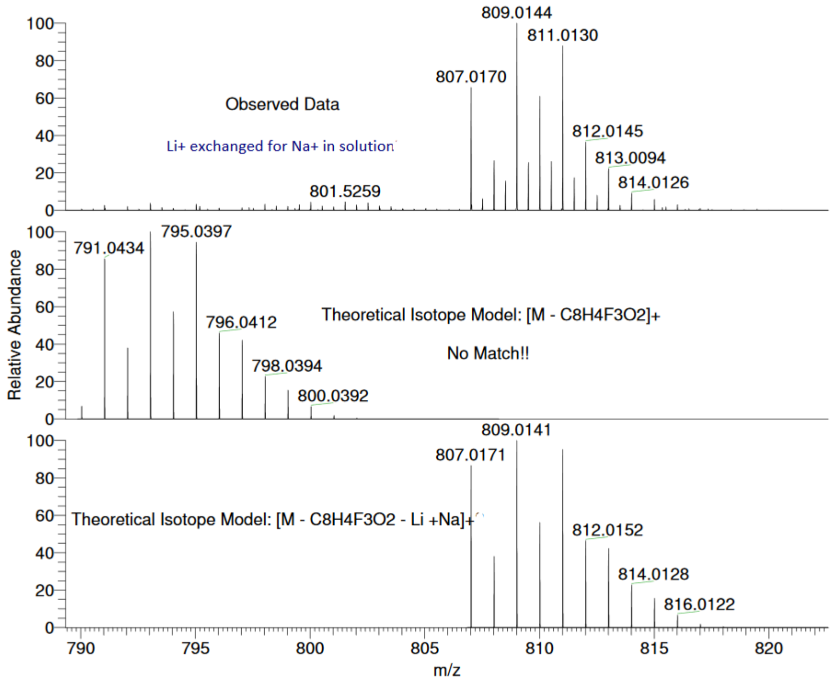

Figure S 74: High resolution ESI mass spectrum of **Zn_2Li.** The molecular ion (**M** - $LiCO_2C_6H_4CF_3$+ Na)$^+$ arises from loss of the $CO_2C_6H_4CF_3$ co-ligand and exchange of the central Li(I) ion for a Na(I) ion during ionization. It is proposed that this exchange arises due to the low selectivity of this ancillary ligand for lithium (over sodium) ion coordination.

Section S11: Synthesis of Zn_2Ca and its Polymerisation Kinetics

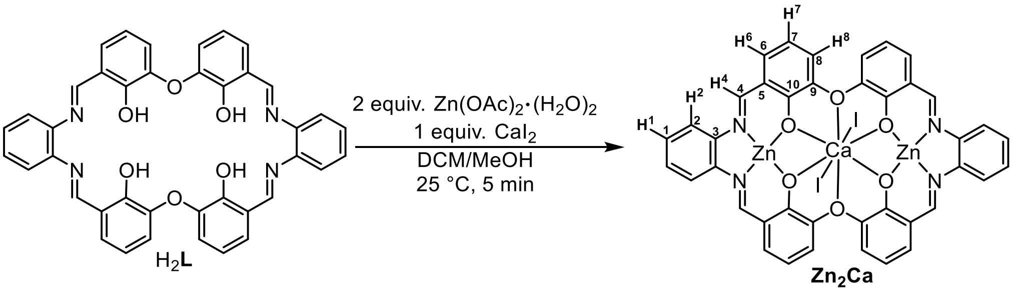

Scheme S 7: Synthesis of **Zn_2Ca** and NMR assignment numbering.

Synthesis of Zn_2Ca: A solution of $Zn(OAc)_2{\cdot}(H_2O)_2$ (33.3 mg, 151 µmol) and CaI_2 (22.3 mg, 75 µmol) in MeOH (3 mL) was added to a solution of H_2**L** (50.0 mg, 75 µmol) in DCM (2 mL). The resulting solution was left unperturbed for 5 min during which an orange precipitate appeared which was isolated by centrifugation, washed with Et_2O (20 mL) and dried in vacuo to yield **Zn_2Ca** as an orange solid (64 mg, 59 µmol, 79%).

^{1}H NMR (500 MHz, d$_6$-DMSO) δ 9.12 (s, 1H, H4), 7.97 (s, 1H, H2), 7.58 (s, 1H, H1), 7.49 (d, J = 7.8 Hz, 1H, H6), 7.46 (d, J = 7.9 Hz, 1H, H8), 6.80 (t, J = 7.8 Hz, 1H, H7).

^{13}C {^{1}H} NMR (126 MHz, d$_6$-DMSO) δ 165.28, 159.47, 146.53, 139.91, 131.51, 129.26, 120.71, 120.16, 119.73, 118.22, 114.79.

HRESI-MS (positive ionization mode) m/z = $(M\text{-}2I)^{2+}$ calculated 412.9931 found 412.9931

Elemental Analysis (**Zn_2Ca**; $C_{40}H_{30}CaI_2N_4O_9Zn_2$) calculated C 42.3%, H 2.7%, N 4.9% found C 42.6%, H 2.6%, N 4.9%

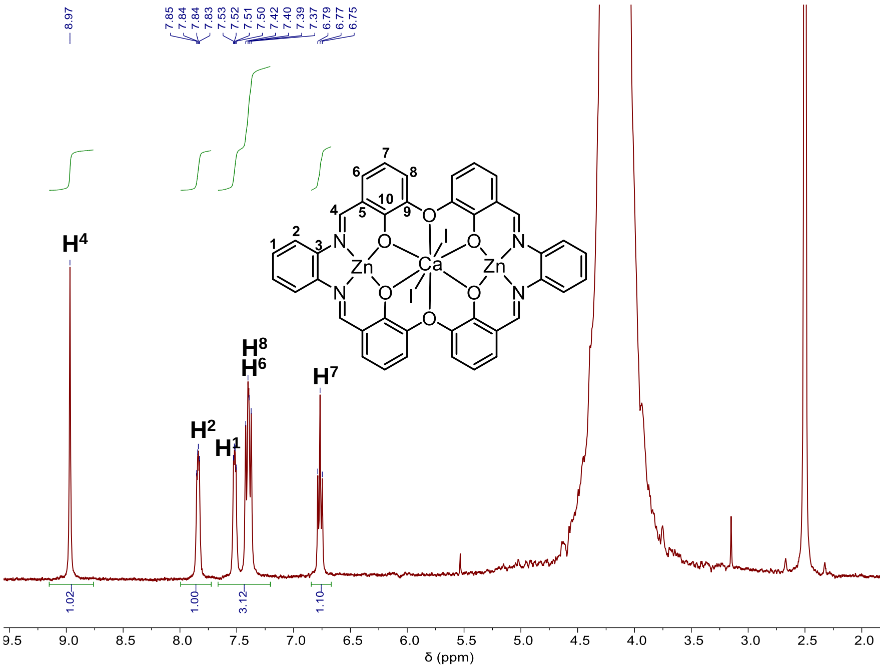

Figure S 75: ^{1}H NMR spectrum (500 MHz, d$_6$-DMSO, 25°C) of **Zn_2Ca**.

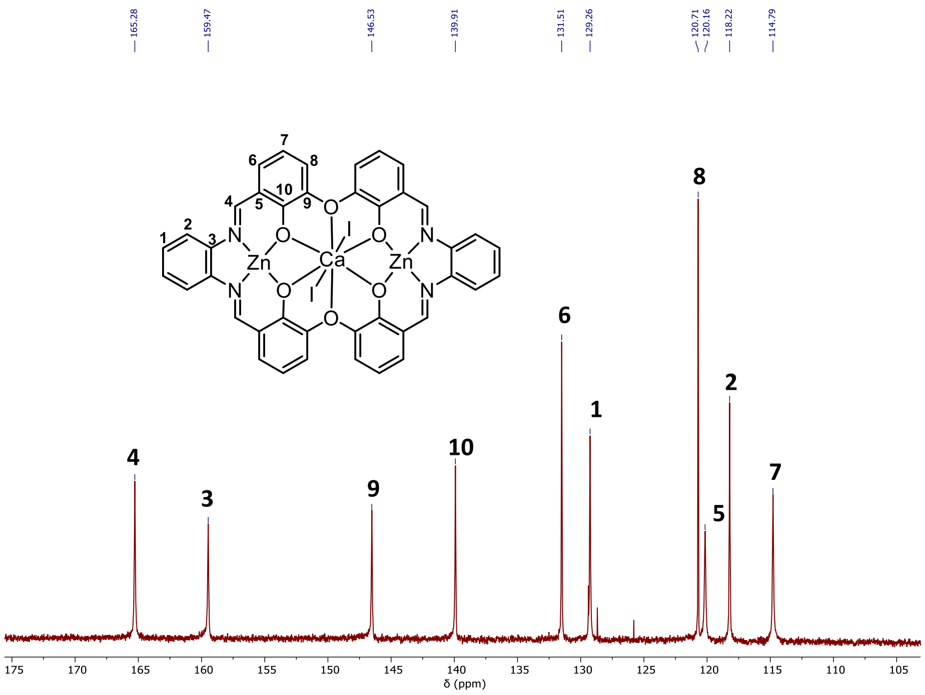

Figure S 76: ^{13}C NMR spectrum (136 MHz, d_6-DMSO, 25°C) of **Zn_2Ca**.

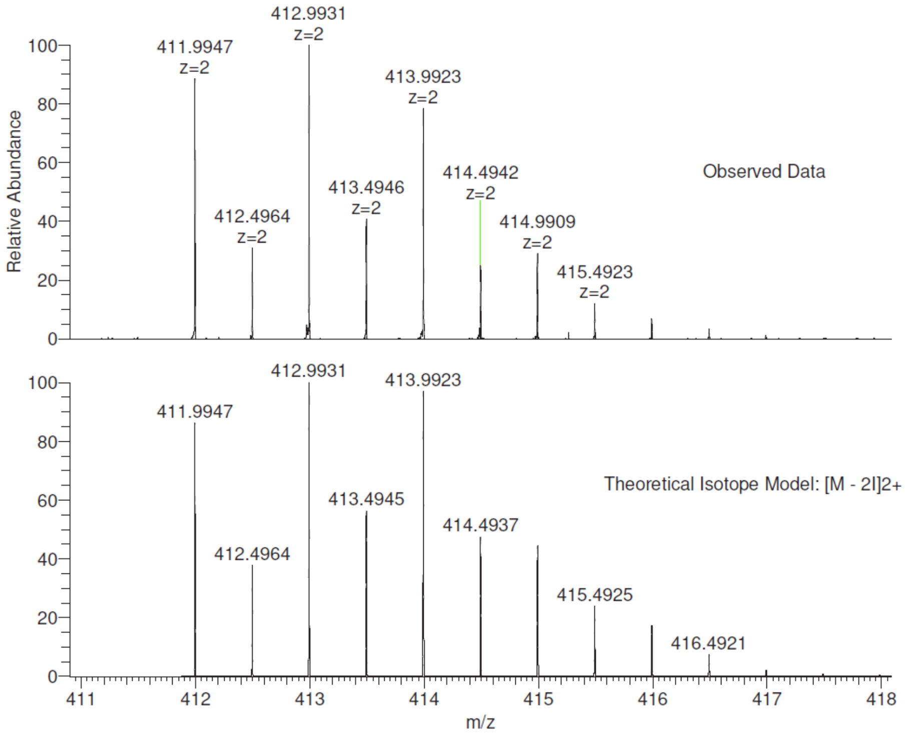

Figure S 77: High resolution ESI mass spectrum of **Zn_2Ca**. The molecular ion $(M\text{-}2I)^{2+}$ arises from loss of two iodide co-ligands during ionization.

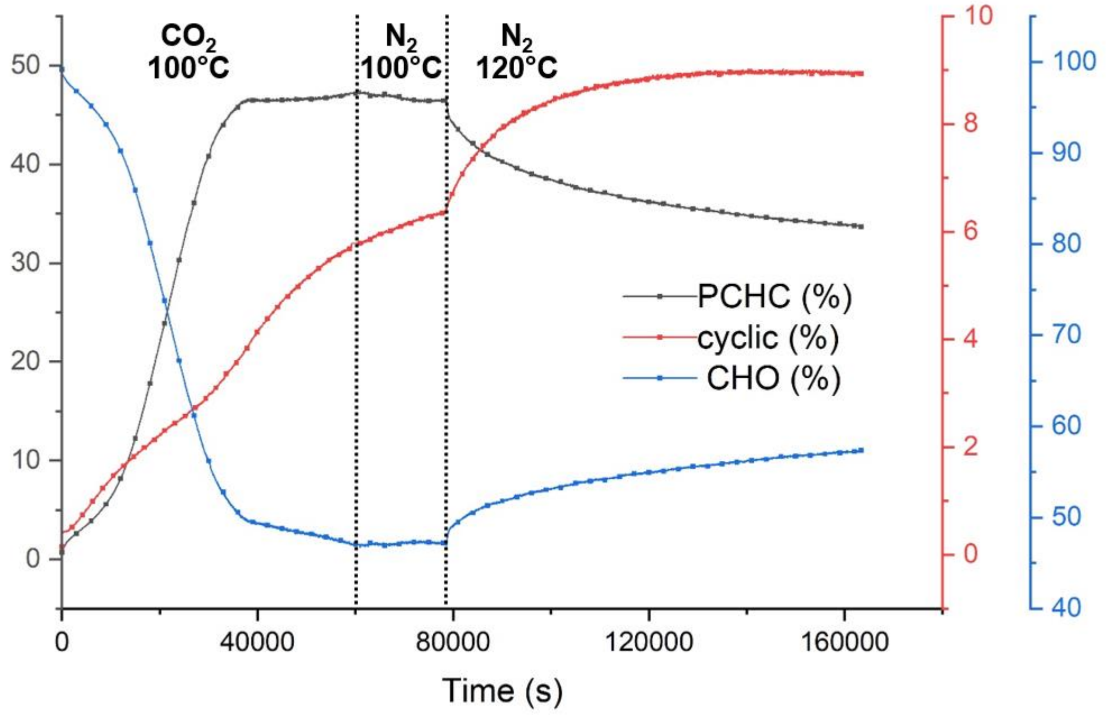

Figure S 78: Plot showing conversion vs. time for CHO/CO_2 ROCOP. Polymerization conditions: 1 bar CO_2, 1 equiv. **Zn_2Ca**, 20 equiv. CHD, 4000 equiv. CHO at 100 °C. After 60,000 s the gas was switched to N_2 and after 78,000 s the temperature was increased to 120 °C.

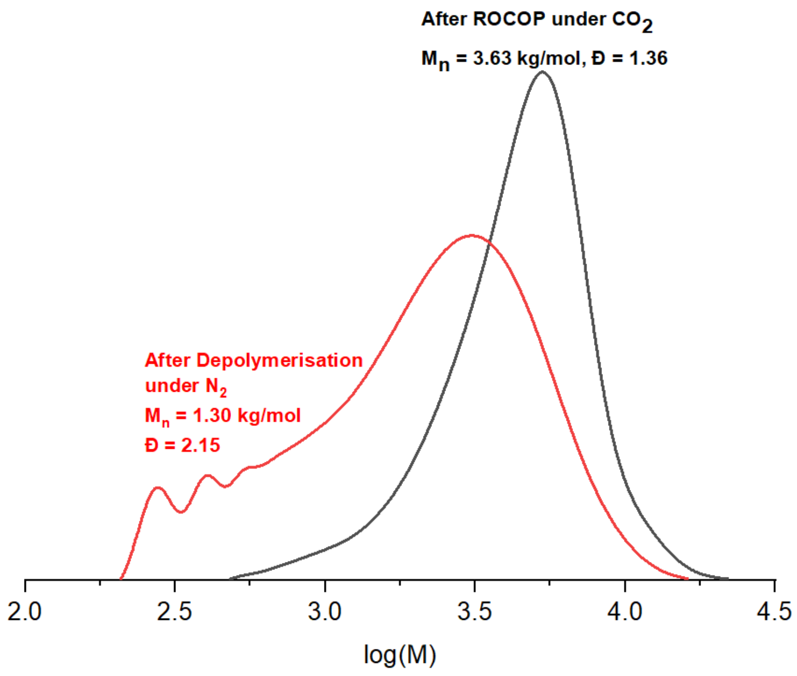

Figure S 79: GPC traces of aliquots taken from the reaction conducted under CO_2 (black) and at the end of the reaction after the gas atmosphere was switched to nitrogen and temperature was increased to 120°C (red) (**Figure S 78**).

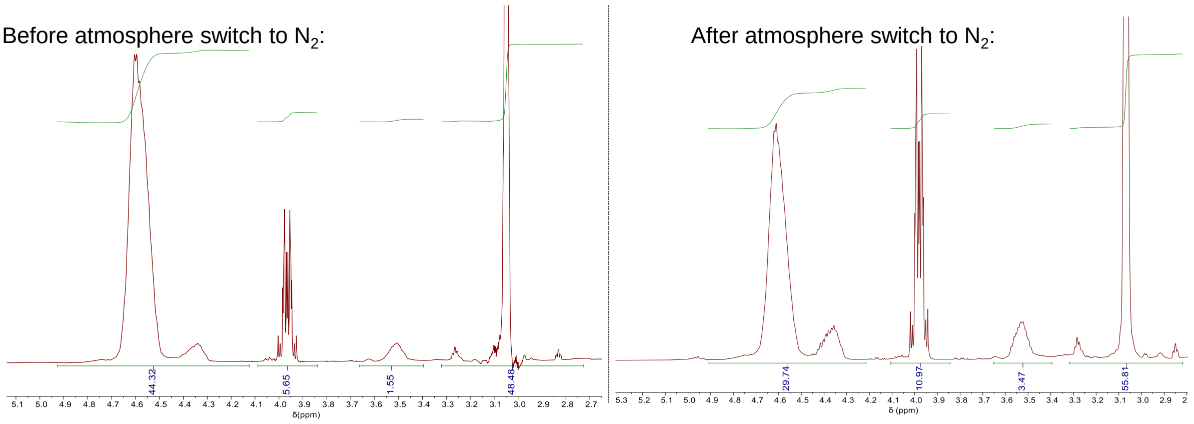

Figure S 80*:* ^{1}H NMR spectrum (400 MHz, $CDCl_3$, 25°C) of polymer produced using **Zn_2Ca**. The spectrum on the left shows the product after CHO/CO_2 ROCOP; the spectrum on the right shows the product after the gas was switched to N_2 and indicates depolymerization occurred (**Figure S 78**).

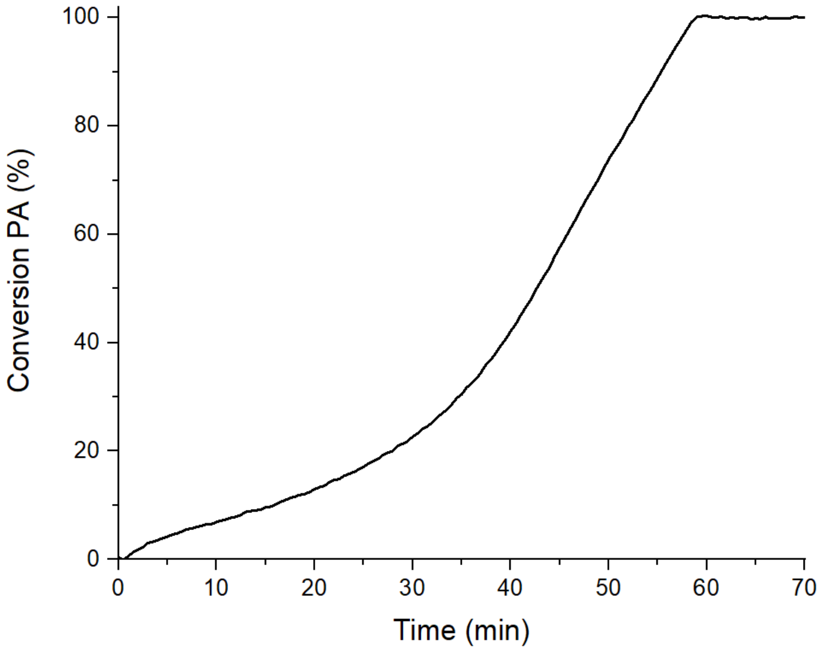

Figure S 81: Conversion vs. time plot for PA/CHO ROCOP. Polymerization conditions: 1 bar N_2, 1 equiv. **Zn_2Ca**, 20 equiv. CHD, 200 equiv. PA and 4000 equiv. CHO at 100 °C. TOF of linear section 853 h^{-1} which equates to 427 h^{-1} per initiator.

Section S12: Synthesis of Zn_2Sr

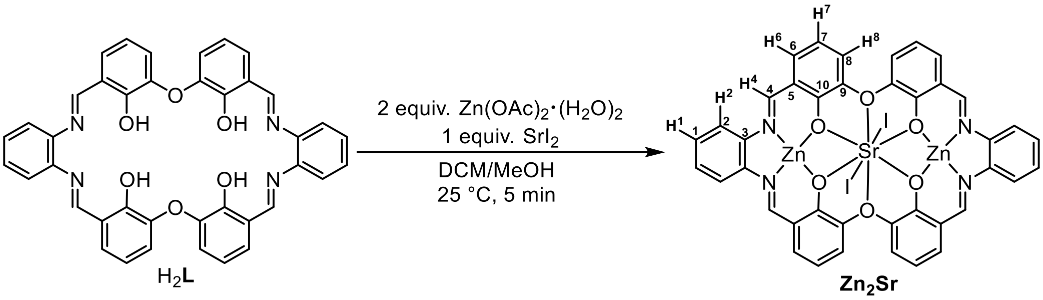

Scheme S 7: Synthesis of **Zn_2Sr** and NMR assignment numbering.

Synthesis of Zn_2Sr: A solution of $Zn(OAc)_2{\cdot}(H_2O)_2$ (33.3 mg, 151 µmol) and SrI_2 (25.6 mg, 75 µmol) in MeOH (3 mL) was added to a solution of H_2**L** (50.0 mg, 75 µmol) in DCM (2 mL). The resulting solution was left unperturbed for 5 min, during which an orange precipitate appeared which was isolated by centrifugation, washed with Et_2O (20 mL) and dried in vacuo to yield **Zn_2Sr**$\cdot 2H_2O$ as an orange solid (92.5 mg, 69 µmol, 92%).

^{1}H NMR (500 MHz, d$_6$-DMSO) δ 9.12 (s, 1H, H4), 7.98 (dd, *J* = 6.1, 3.4 Hz, 1H, H2), 7.57 (dd, *J* = 6.0, 3.3 Hz, 1H, H1), 7.30-7.50 (m, 2H, H6/8), 6.77 (t, *J* = 7.9 Hz, 1H,H7).

^{13}C {^{1}H} NMR (126 MHz, d$_6$-DMSO) δ 165.17, 160.51, 146.99, 139.89, 131.26, 129.13, 120.99, 119.82, 118.17, 114.39.

HRESI-MS (positive ionization mode) m/z = (M-2I)$^{2+}$ calculated 436.9651 found 436.9653

Elemental Analysis (**Zn_2Sr**; $C_{40}H_{28}SrI_2N_4O_9Zn_2$) calculated C 41.2%, H 2.4%, N 4.6% found C 41.4%, H 2.4%, N 4.8%

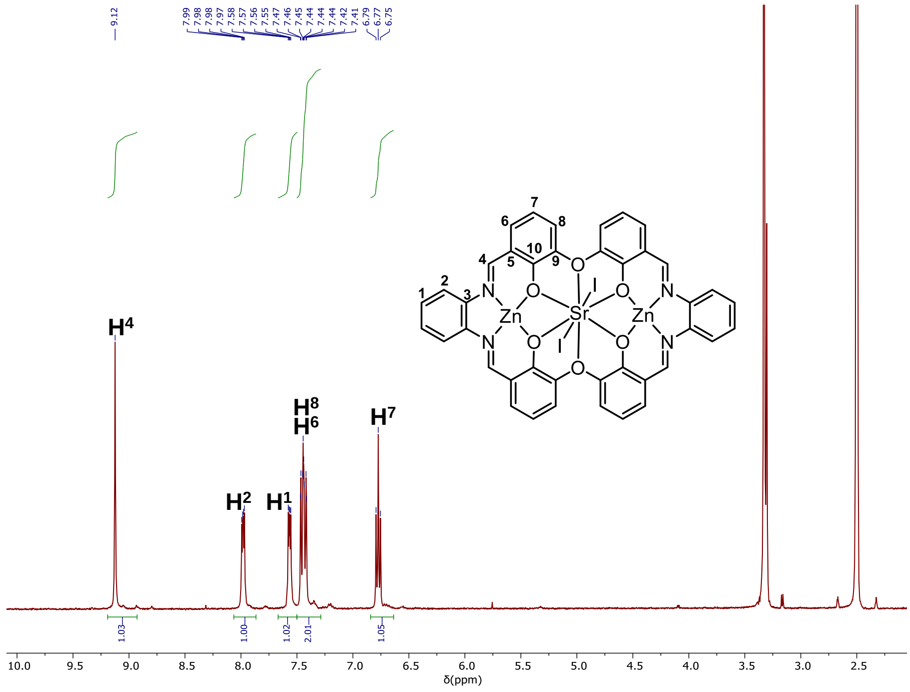

Figure S 82: ^{1}H NMR spectrum (500 MHz, d$_6$-DMSO, 25°C) of **Zn_2Sr**.

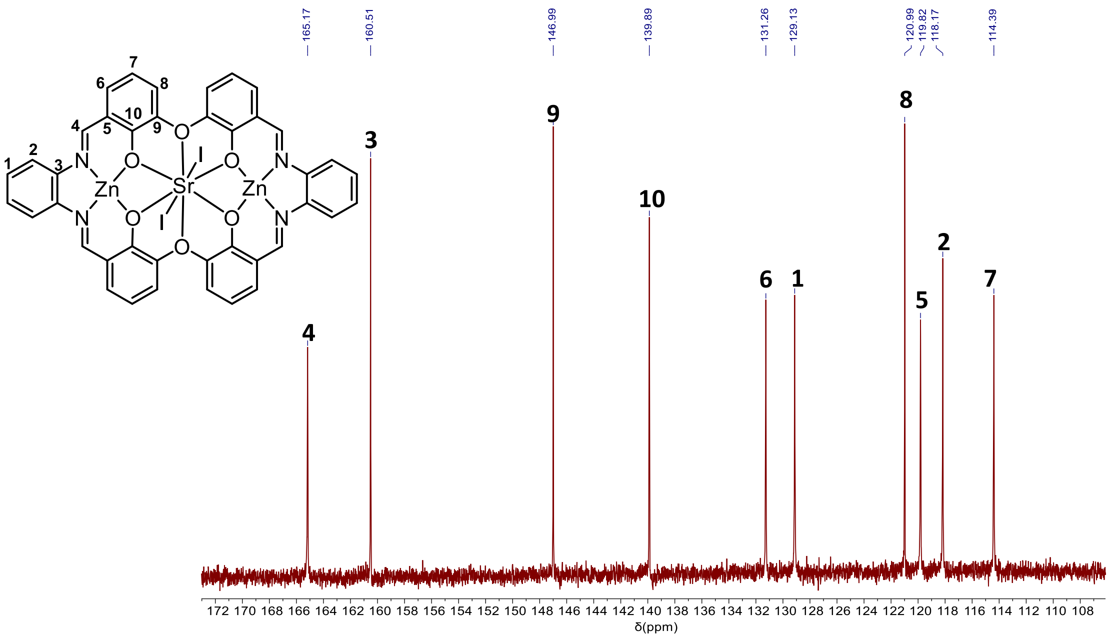

Figure S 83: $^{13}C\ \{^{1}H\}$ NMR spectrum (136 MHz, d_6-DMSO, 25°C) of **Zn_2Sr**.

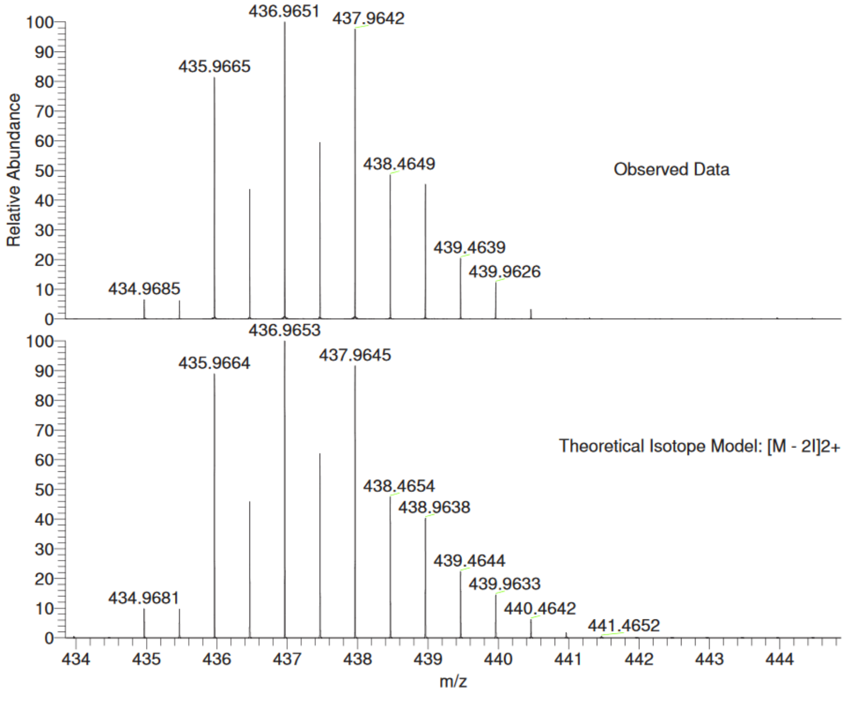

Figure S 84: High resolution ESI mass spectrum of **Zn_2Sr**. The molecular ion $(M\text{-}2I)^{2+}$ arises from the loss of two iodide co-ligands during ionization.

Section S13: Synthesis of Zn_2La and its Polymerisation Kinetics

Synthesis of $(CF_3C_6F_4CO_2)_3La$

4-(Trifluoromethyl)benzoic acid (500.0 mg, 2.6 mmol, 1 equiv.) was suspended in deionized water (100 mL). Next, NaOH (105.0 mg, 2.6 mmol, 1 equiv.) was added and the mixture was stirred for 1 h, at room temperature. The mixture was filtered and $LaCl_3$ (150.0 mg, 0.6 mmol, 0.2 equiv.), dissolved in deionized water (10 mL), was added resulting in the immediate formation of a thick white precipitate. The precipitate was isolated by filtration, washed with deionized water (200 mL) and Et_2O (200 mL) and dried under vacuum yielding crude $(CF_3C_6F_4CO_2)_3La$ as a colourless powder (494.2 mg, 0.4 mmol, 70% yield). The material was used without further purification.

^{1}H NMR (400 MHz, MeOD) δ 8.08 (d, *J* = 7.9 Hz, 1H, H3), 7.72 (d, *J* = 7.9 Hz, 1H, H4).

^{19}F {^{1}H} NMR (376 MHz, MeOD) δ -59.71.

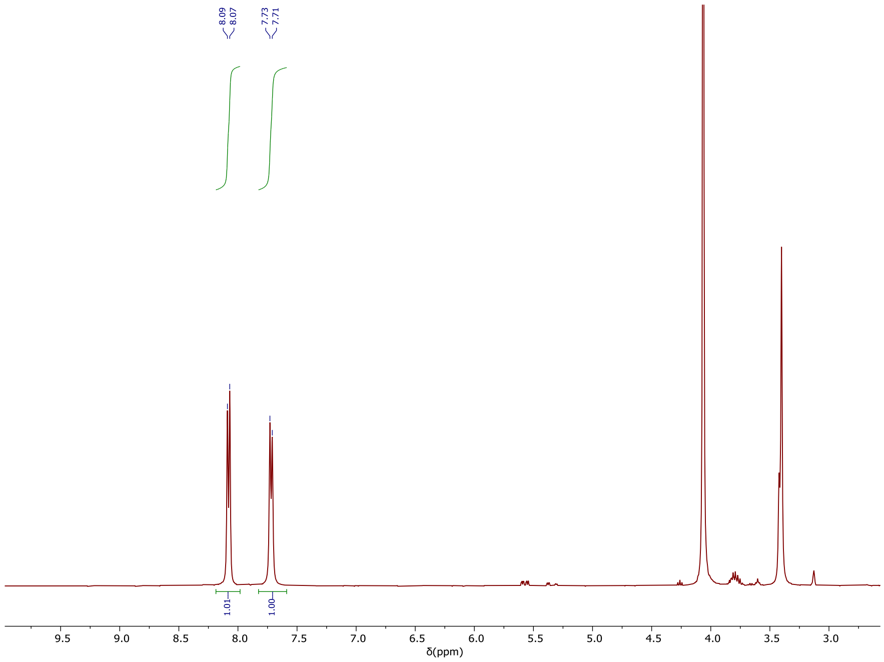

Figure S 85: ^{1}H NMR spectrum (400 MHz, MeOD, 25°C) of **$(CF_3C_6F_4CO_2)_3La$**.

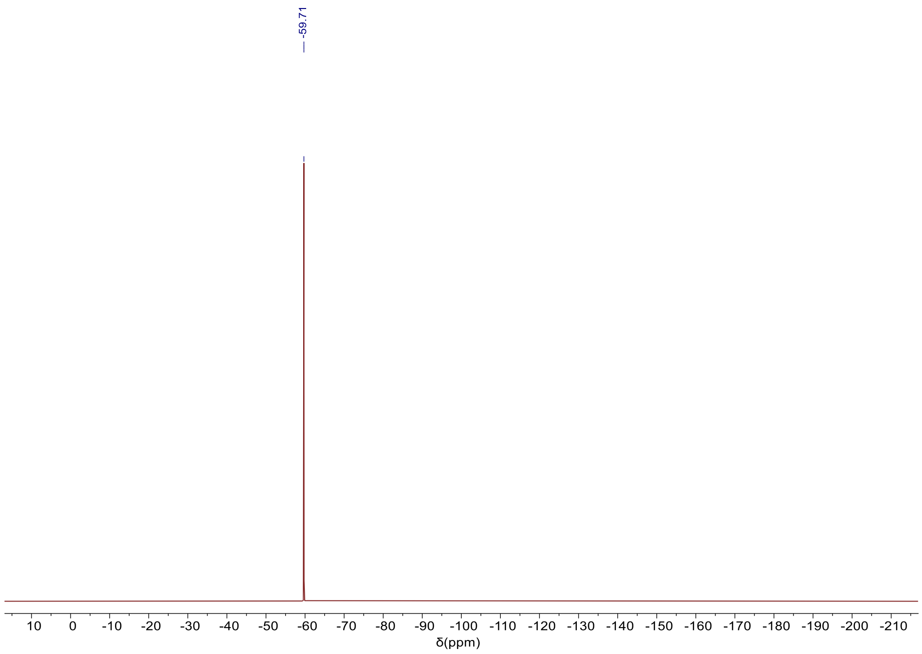

Figure S 86: ^{19}F {^1H} NMR spectrum (376 MHz, MeOD, 25°C) of **($CF_3C_6F_4CO_2$)$_3$La**.

Synthesis of Zn_2La

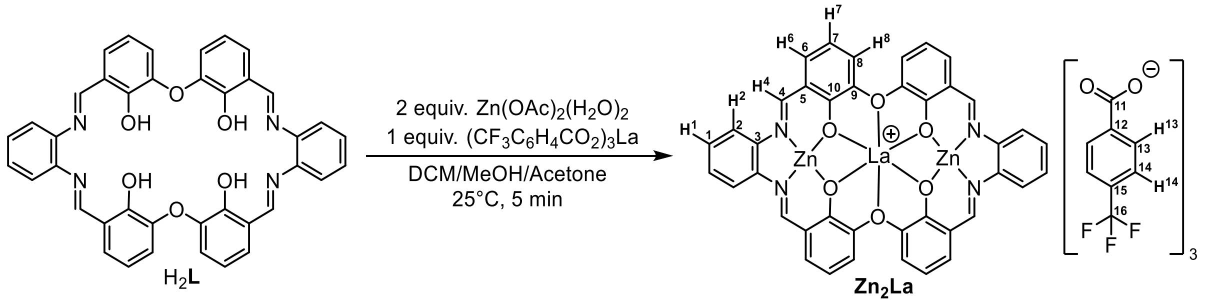

Scheme S 7: Synthesis of **Zn_2La** and NMR assignment numbering.

Synthesis of Zn_2La: A solution of $Zn(OAc)_2{\cdot}(H_2O)_2$ (33.3 mg, 151 µmol), in MeOH (5 mL), and a solution ($CF_3C_6F_4CO_2$)$_3$La (53.5 mg, 75 µmol), in acetone (5 mL), were sequentially added to a solution of H_2**L** (50.0 mg, 75 µmol), in DCM (5 mL). The resulting solution was left unperturbed for 5 min. Afterwards all volatiles were removed, in vacuo, yielding a semi-solid which was washed with Et_2O (20 mL). In order to remove residual AcOH by-product, the crude material was suspended in toluene (20 mL) which was afterwards removed in vacuo. This process was repeated yielding **Zn_2La**·$4H_2O$ as a yellow powder (109 mg, 73 µmol, 96%).

^1H NMR (500 MHz, d_6-DMSO) δ 9.05 (s, 1H, H4), 7.92 (m, 2.5H, H13/H2), 7.67 – 7.47 (m, 3.5H, H14/H1/H6), 7.39 (d, J = 7.4 Hz, 1H, H8), 6.76 (t, J = 8.0 Hz, 1H, H7).

^{13}C{^1H} NMR (126 MHz, d_6-DMSO) δ 169.73, 164.99, 159.73, 147.03, 140.58, 131.20-129.81 (m), 129.07, 124.83, 123.26 (q, J = 271 Hz), 120.89, 119.30, 118.02, 114.53.

^{19}F{^1H} NMR (470 MHz, d_6-DMSO) δ -61.15.

HRESI-MS (positive ionization mode) m/z = (M - $CF_3C_6F_4CO_2$)$^+$ calculated 1304.9624 found 13094.9624

Elemental Analysis (**Zn_2La·**$4H_2O$; $C_{64}H_{44}F_9LaN_4O_{16}Zn_2$) calculated C 49.1% H 2.8% N 3.6% found C 49.0% H 2.4% N 3.3%

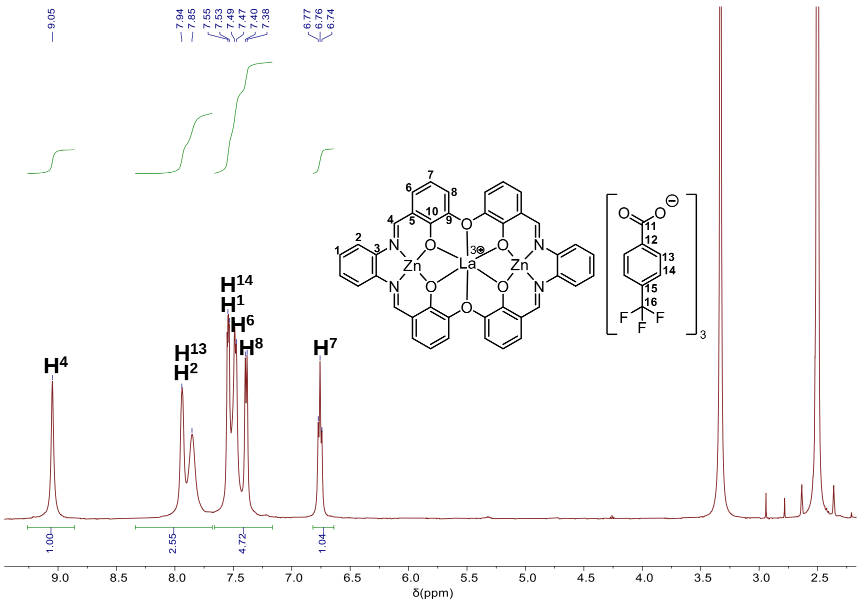

Figure S 87: ^{1}H NMR spectrum of **Zn_2La** (500 MHz, d_6-DMSO, 25°C).

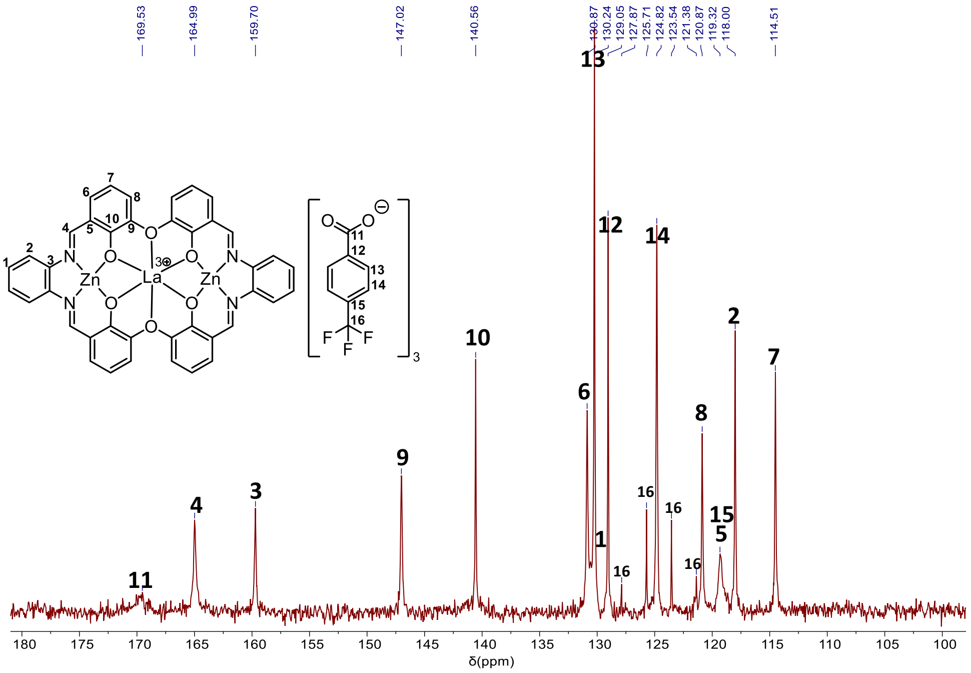

Figure S 88: ^{13}C{^{1}H} NMR spectrum of **Zn_2La** (500 MHz, d_6-DMSO, 25°C).

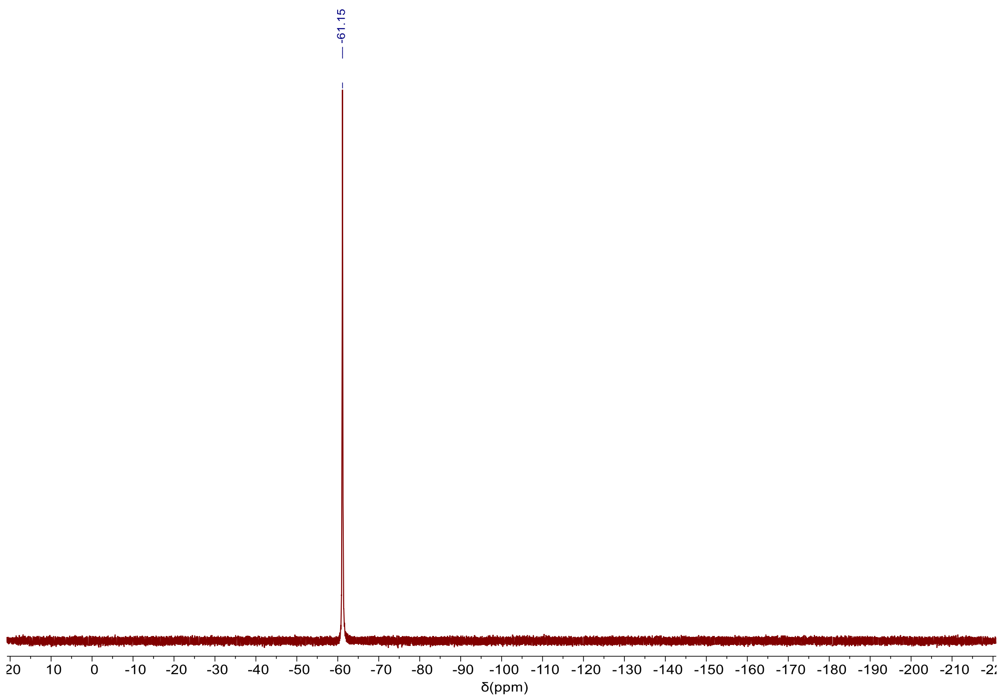

Figure S 89: $^{19}F\{^1H\}$ NMR spectrum of **Zn_2La** (470 MHz, d_6-DMSO, 25°C).

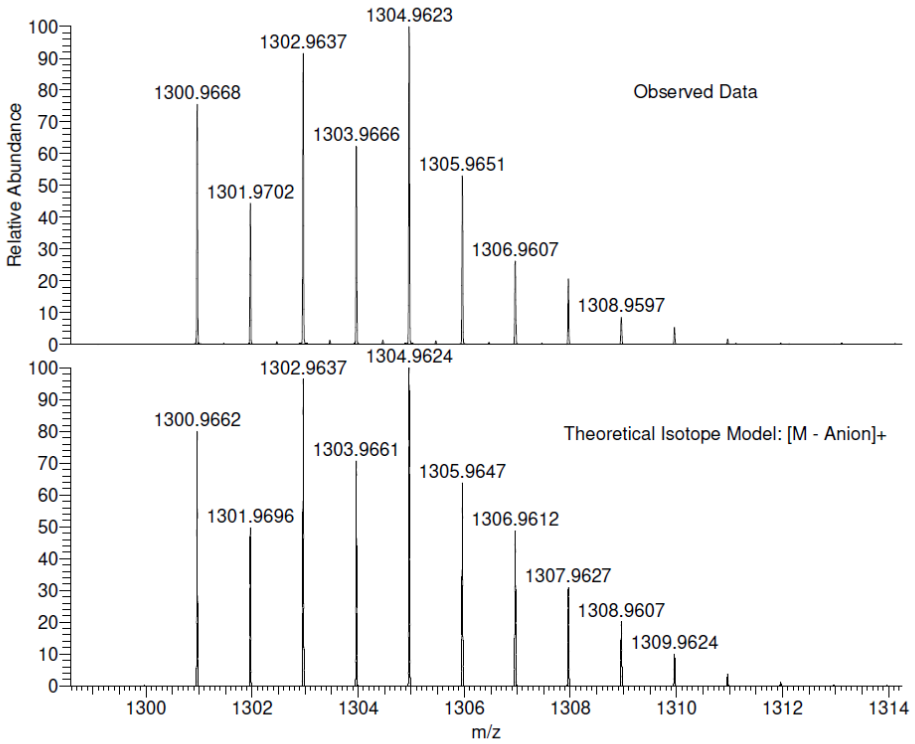

Figure S 90: High resolution ESI mass spectrum of **Zn_2La**. The molecular ion $(M - CF_3C_6F_4CO_2)^+$ arises from loss of one 4-CF_3-benzoate co-ligand during ionization.

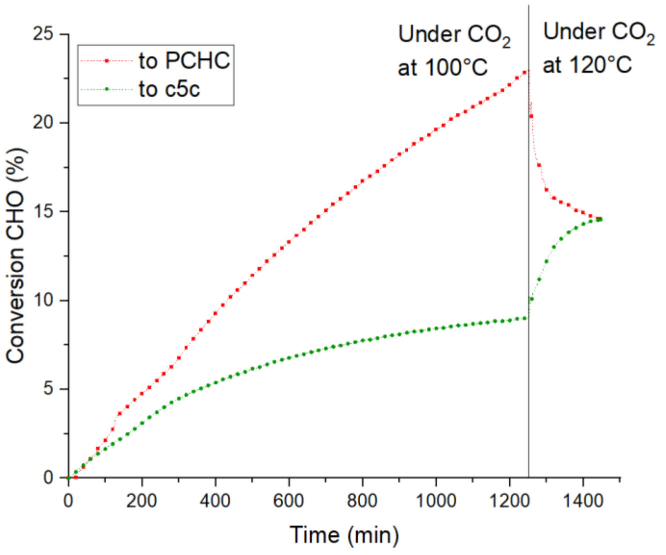

Figure S 91: Plot showing conversion vs. time for CHO/CO_2 ROCOP. Polymerization conditions: 1 bar CO_2, 1 equiv. **Zn_2La**, 20 equiv. CHD, 4000 equiv. CHO at 100 °C. After 1270 min the gas was switched to N_2 and the temperature was increased to 120 °C resulting in depolymerization of PCHC.

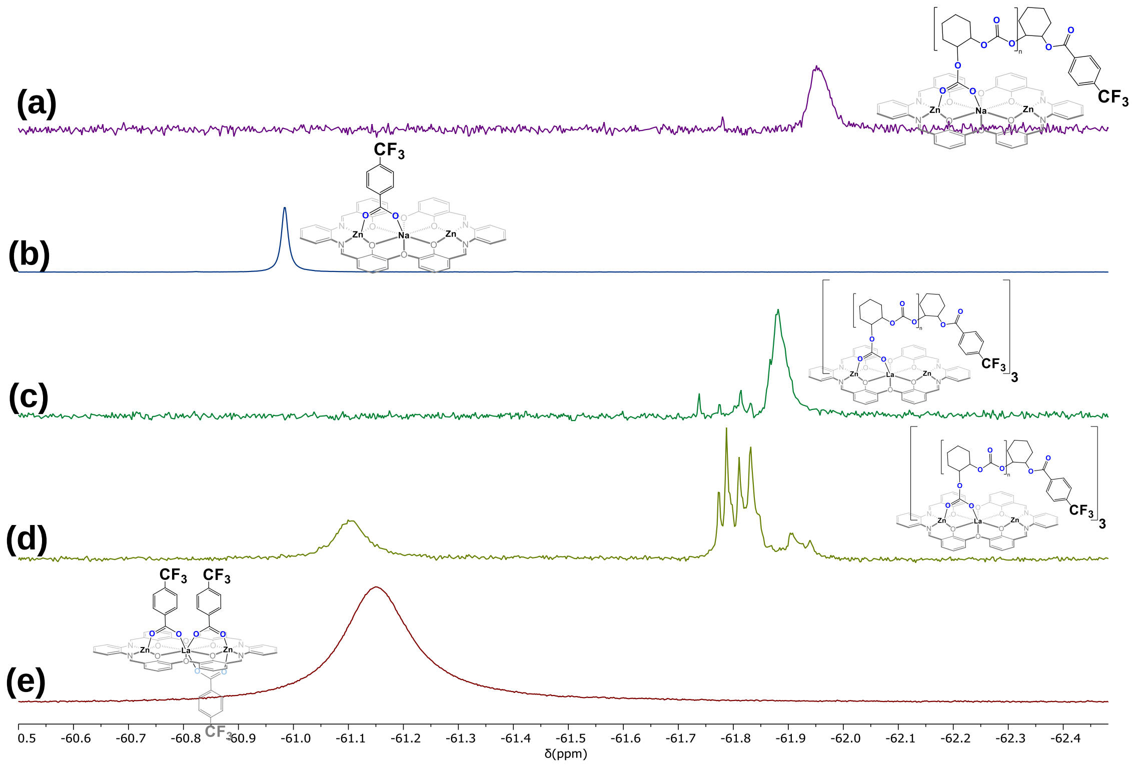

Figure S 92: Comparison of the ^{19}F{^{1}H} NMR spectra (376 MHz, d_6-DMSO , 25°C) of: (a) **Zn_2Na** at the end of CO_2/CHO ROCOP, (b) **Zn_2Na,** (c) **Zn_2La** at the end of CO_2/CHO ROCOP, (d) **Zn_2La** immediately after initiation with an additional equivalent of **Zn_2La** added to show different signals are observable (note that the **Zn_2La** signal is not present unless it is deliberately added), (e) **Zn_2La**.

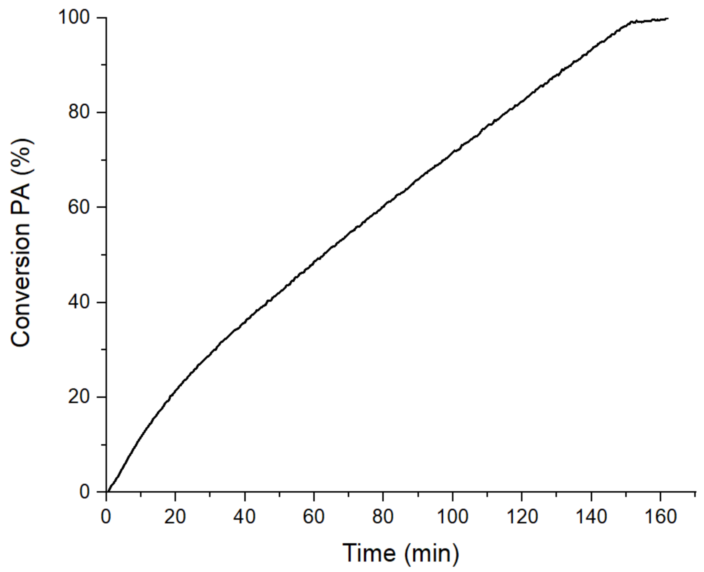

Figure S 93: Conversion vs. time plot for PA/CHO ROCOP. Polymerization conditions: 1 bar N_2, 1 equiv. **Zn_2La**, 20 equiv. CHD, 200 equiv. PA and 4000 equiv. CHO at 100 °C. TOF of linear section is 68 h^{-1} which equates to 23 h^{-1} per initiator.

Section S14: Synthesis of Zn_2Y

Synthesis of $(CF_3C_6F_4CO_2)_3Y$

4-(Trifluoromethyl)benzoic acid (500.0 mg, 2.6 mmol, 1 equiv.) was suspended in of deionized water (100mL). Next, NaOH (10.0 mg, 2.6 mmol, 1 equiv.) was added and the mixture was stirred for 1 h at room temperature. The mixture was filtered and YCl_3 (119mg, 0.61 mmol, 0.23 eq), dissolved in deionized water (10 mL), was added resulting in the immediate formation of a thick white precipitate. The precipitate was isolated by filtration, washed with deionized water (200 mL) and Et_2O (200 mL). The resulting colourless powder was extracted into THF and filtered. Removal of all volatiles in vacuo yielded crude $(CF_3C_6F_4CO_2)_3Y$·THF as a colourless powder (356.2 mg, 0.5 mmol, 81% yield). The material was used without further purification.

^{1}H NMR (400 MHz, d_6-DMSO) δ 8.08 (d, *J* = 8.2 Hz, 6H), 7.68 (d, *J* = 8.3 Hz, 6H), 3.93 – 3.51 (m, 4H), 1.85 – 1.45 (m, 4H).

^{19}F {^{1}H} NMR (376 MHz, d_6-DMSO) δ -59.71.

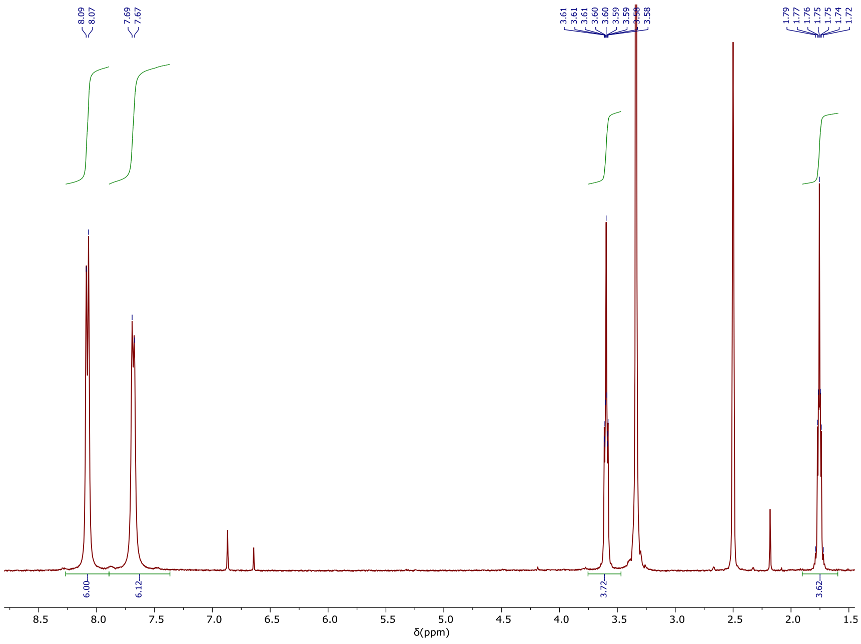

Figure S 94: ^{1}H NMR spectrum of **$(CF_3C_6F_4CO_2)_3Y$** (400 MHz, d_6-DMSO, 25°C).

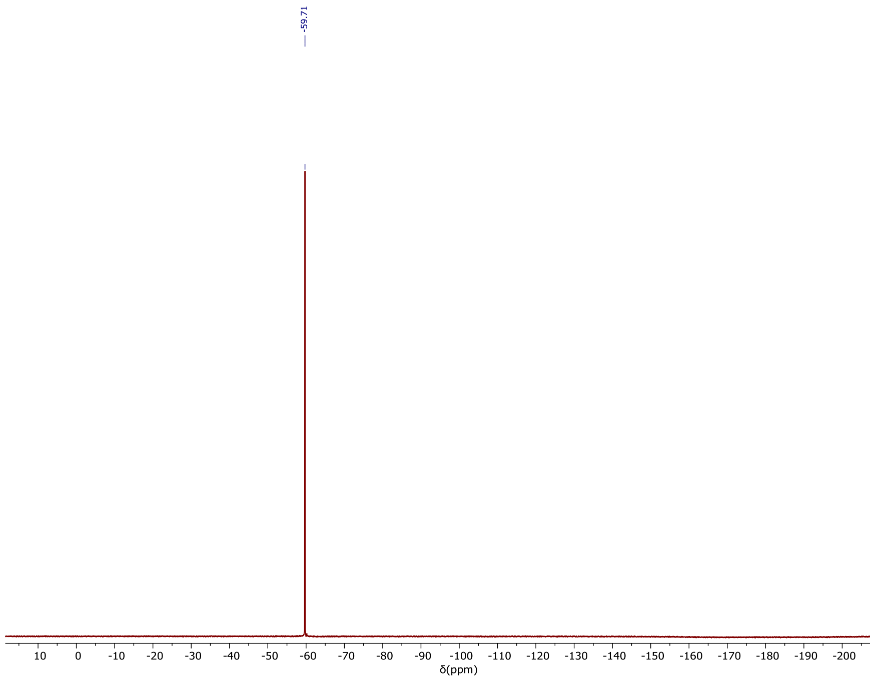

Figure S 95: $^{19}F\{^{1}H\}$ NMR spectrum of **($CF_3C_6F_4CO_2$)$_3$Y**·THF (376 MHz, d_6-DMSO, 25°C).

Synthesis of Zn_2Y

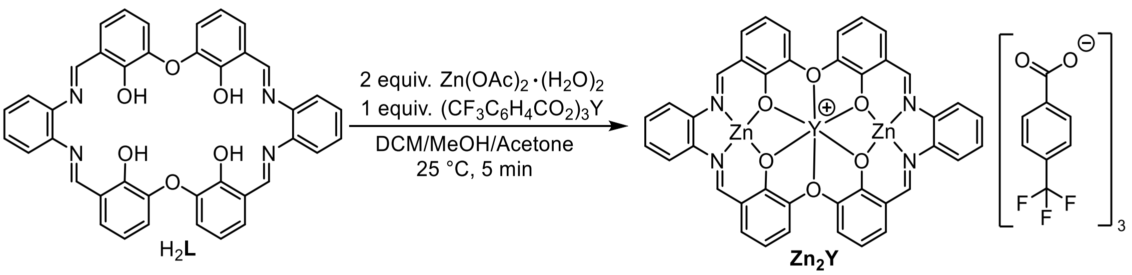

Synthesis of Zn_2Y: A solution of $Zn(OAc)_2 \cdot (H_2O)_2$ (33.3 mg, 151.0 µmol) in MeOH (5 mL) and a solution ($CF_3C_6F_4CO_2$)$_3$Y·THF (54.5 mg, 75.0 µmol) in acetone (5 mL) were sequentially added to a solution of H_2**L** (50.0 mg, 75.0 µmol) in DCM (5mL). The resulting solution was left unperturbed for 5 min. Afterwards all volatiles were removed in vacuum yielding a semi-solid which was washed with Et_2O (20mL). In order to remove residual AcOH byproduct, the crude material was suspended in toluene (20 mL) which was afterwards removed in vacuum. This process was repeated yielding **Zn_2Y·**$2H_2O$ as a yellow powder (104mg, 73 µmol, 96%).

Conclusive NMR analysis was prevented by the broadness of the spectrum.

HRESI-MS (positive ionization mode) m/z = (M - $CF_3C_6F_4CO_2$)$^+$ calculated 1252.9631 found 1252.9638

Elemental Analysis (**Zn_2Y·**$2H_2O$; $C_{64}H_{40}F_9N_4O_{14}YZn_2$) calculated C 51.9% H 2.7% N 3.8% found C 51.9% H 2.5% N 4.0%

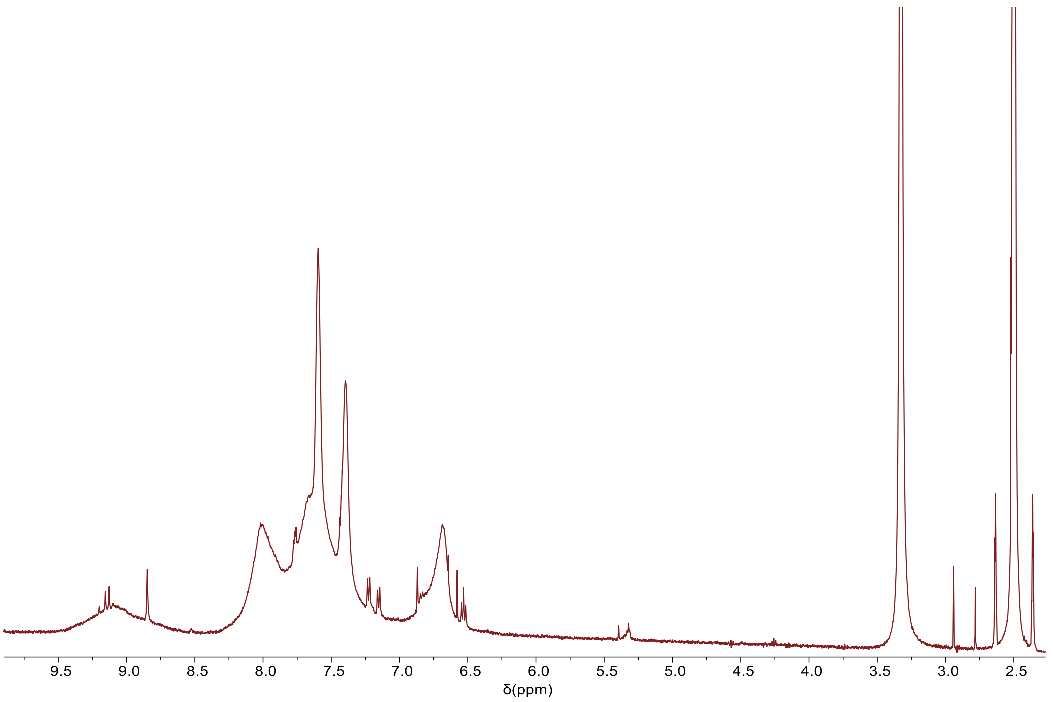

Figure S 96: ^{1}H NMR spectrum of **Zn$_2$Y** (500 MHz, d_6-DMSO, 25°C).

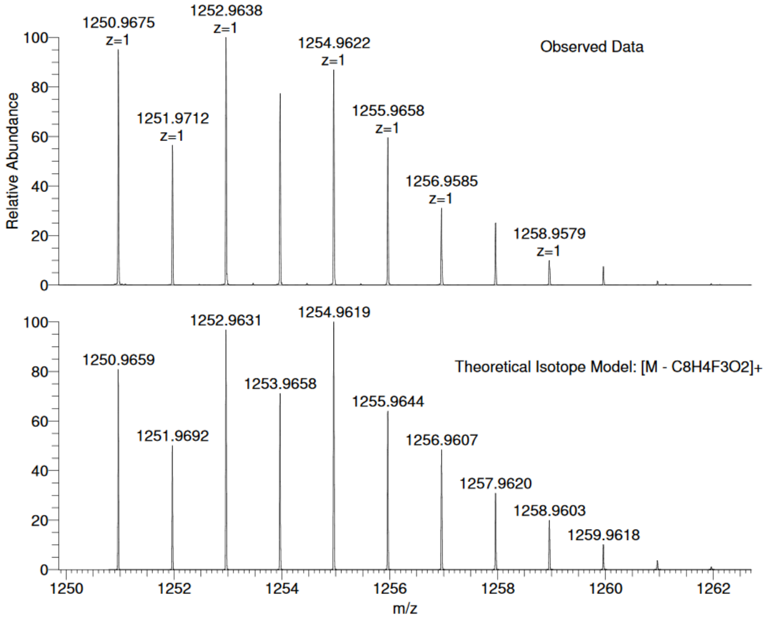

Figure S 97: High resolution ESI mass spectrum of **Zn$_2$Y**. The molecular ion (M - $CF_3C_6F_4CO_2$)$^+$ corresponds to the loss of a $CF_3C_6F_4CO_2$ co-ligand during ionization.

Section S15: Polymerisation Kinetics of Zn_2+PPNCl

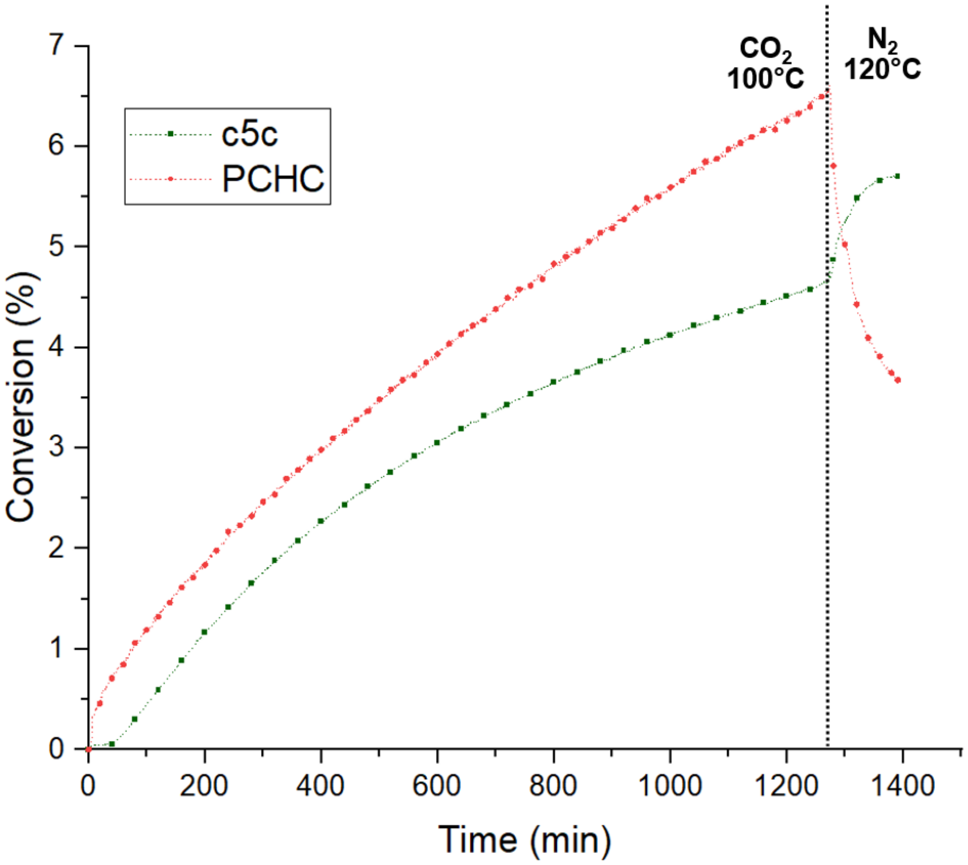

Figure S 98: Plot showing conversion vs. time for CHO/CO_2 ROCOP. Polymerization conditions: 1 bar CO_2, 1 equiv. $\mathbf{Zn_2}$, 1 equiv. PPNCl, 20 equiv. CHD, 4000 equiv. CHO at 100 °C. After 1280 min the gas was switched to N_2 and the temperature was increased to 120 °C resulting in depolymerization of PCHC.

References

[1] Akine, S., et al., *Inorg. Chem.* **55**, 810–821 (2016)

[2] Anouti, M., et al., *J. Chem. Thermodynamics* **50**, 71–79 (2012)
